# Thermal Safety Frontiers in Electrochemical Energy Storage: Thermal Runaway Mechanisms, Intelligent Sensing, and Multiscale Suppression Strategies

**DOI:** 10.1007/s40820-026-02340-3

**Published:** 2026-11-02

**Authors:** Liangyu Li, Zhiying Lai, Xianyong Wu, Zhongxue Chen

**Affiliations:** 1 Key Laboratory of Hydraulic Machinery Transients, Ministry of Education, School of Power and Mechanical Engineering, Wuhan University, Wuhan, 430072 People’s Republic of China; 2 Department of Chemistry, University of Puerto Rico, Río Piedras Campus, San Juan, PR 00925-2537 USA

**Keywords:** Thermal runaway, Battery safety, Intelligent sensing, Active-response materials, Multiscale protection

## Abstract

With the continuous pursuit of high energy density in electrochemical energy storage systems, the thermal safety of lithium-ion batteries has emerged as a critical bottleneck restricting their large-scale application. This article systematically reviews the frontier advances in the field of lithium-ion battery thermal safety, innovatively establishing a comprehensive knowledge framework spanning from microscopic mechanisms to macroscopic system-level protection. First, it provides an in-depth analysis of the triggering mechanisms of thermal runaway and the cross-scale chain reaction evolution pathways, summarizing mechanism-based and data-driven modeling and prediction methods. Second, it focuses on real-time intelligent monitoring and early warning technologies for thermal runaway, constructing a full-process technical system covering “sensing-identification-warning”. In terms of cell-level protection, it systematically outlines the paradigm evolution of cell materials from traditional passive protection toward active-response approaches. Finally, it discusses thermal management and engineering protection strategies at the system integration level, summarizing the critical pathways for enhancing system safety design and failure response capabilities. This review aims to provide a systematic theoretical perspective for resolving the inherent trade-off between energy and safety in high-energy-density batteries and to offer comprehensive theoretical insights and innovative technological pathways for the next generation of energy storage systems.

## Highlights

- A cross-scale “mechanism-sensing-protection” full-chain knowledge framework has been established, providing a panoramic theoretical perspective for battery thermal safety research.
- The paradigm evolution of intelligent materials from “passive protection to active response” is delineated, revealing its underlying evolutionary trajectory.
- A system-level thermal safety design philosophy characterized by “internal-external synergy and multi-level defense” is proposed, offering engineering guidance for achieving the unification of high energy density and high safety.

## Graphical abstract

**Figure:**
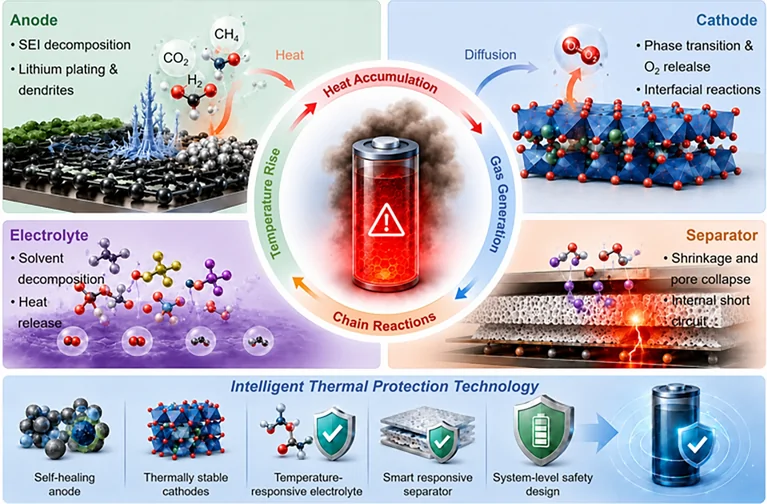

## Introduction

Driven by the global energy transition and the ambitious goal of carbon neutrality, electric vehicles (EVs) and large-scale electrochemical energy storage have experienced explosive growth [1–3]. To alleviate range anxiety and reduce system costs, lithium-ion batteries (LIBs) have achieved leapfrog development over the past decade, with energy densities increasing from 150 Wh kg^−1^ to over 300 Wh kg^−1^ [4, 5]. This advancement is primarily attributed to the introduction of advanced, highly reactive material systems such as nickel-rich cathodes [6, 7] and silicon-carbon anodes. However, this increase in energy density inevitably comes at the cost of reduced thermodynamic and kinetic stability, leading to a sharp narrowing of the thermal safety boundaries of battery systems [8]. In recent years, frequent fire and explosion accidents involving electric vehicles have not only caused significant loss of life and property but have also raised widespread public concern regarding battery safety. Achieving ultimate energy density while ensuring robust safety has thus become a critical bottleneck faced by both academia and industry.

Tracing the root cause, the fundamental reason for lithium-ion battery fires and explosions is thermal runaway (TR) induced by internal faults. Thermal runaway is not an instantaneous event but a catastrophic chain reaction process consisting of a series of spontaneous and mutually reinforcing exothermic side reactions under extreme abuse conditions (thermal, electrical, and mechanical coupling) [9–12]. Macroscopically, it manifests as an exponential surge in temperature, ultimately culminating in oxygen release from the cathode [13–15]. These reactions occur sequentially, with each step adding to the heat accumulation. Critically, these core components do not fail in isolation but are coupled through chemical crosstalk between electrodes. Active oxygen and transition-metal ions released from the cathode migrate to the anode, catalyzing solid-electrolyte interphase (SEI) decomposition and inducing lithium dendrite growth [16], conversely reducing gases generated at the anode diffuse to the cathode, accelerating the solvation of transition-metal ions. This bidirectional crosstalk constitutes a cross-scale accelerating failure network [17, 18]. Once a single cell undergoes thermal runaway, it can easily trigger a domino-effect system-level thermal propagation through heat conduction and jet impact, leading to the complete destruction of the entire energy storage unit. The chain reaction of thermal runaway involves the synergistic failure of multiple core components within the battery. The hazards are multilayered and progressive: direct hazards include violent physical explosion and jetting; secondary hazards include the release of large quantities of toxic and flammable gases (e.g., CO, HF) and submicron toxic particles, posing significant long-term health and environmental risks [19]. Moreover, the major safety threat of thermal runaway lies not only in its destructiveness upon occurrence but also in the stealthiness of its triggers and the difficulty of post-event investigation. Traditional passive protection often only delays the disaster, and firefighting operations can easily destroy critical evidence, rendering conventional post hoc investigation methods extremely challenging. Therefore, establishing a full-chain thermal safety technology system encompassing “mechanism tracing,” “pre-warning,” and “propagation blocking” is of urgent practical significance [7].

In response to this highly destructive thermal runaway, researchers are pursuing systematic countermeasures across multiple dimensions, following a core logic that advances progressively through mechanistic understanding, risk perception, and active protection. First, a deep understanding of thermal runaway mechanisms forms the cornerstone for constructing a safety technology system. Based on this understanding, researchers have developed a range of modeling and prediction methods, from reaction kinetic models [20], thermal models [21], and electrical models [22] to multiphysics coupled models [23], aiming to achieve accurate simulation and risk quantification of the thermal runaway evolution process, thereby providing decision support for proactive protection [24]. However, even the most precise models cannot replace the real-time perception of the battery’s actual state. During the incubation period of thermal runaway, traditional threshold-based voltage and temperature warnings often fail due to signal lag [25], necessitating the development of more sensitive and earlier-stage monitoring technologies. Consequently, researchers have turned their attention to external non-invasive monitoring techniques [26] and internal embedded sensors [27]. By fusing multi-dimensional signals such as temperature, pressure, strain, acoustic emission, and characteristic gases, combined with model-driven and data-driven intelligent diagnostic algorithms, the goal is to capture early abnormal signals before the thermal runaway chain reaction is fully established, thereby gaining a crucial time window for active safety responses [26, 28, 29].

Once an early warning signal is triggered, the final line of defense lies in material and system-level protection. At the cell material level, safety design is undergoing a profound evolution from traditional passive protection toward active-response intelligent materials. Passive protection strategies primarily enhance thermal stability through intrinsic material modifications, such as single-crystallization and doping/coating of cathodes [30], carbon coating and three-dimensional structural design of anodes [31], fluorination modification and flame-retardant additives in electrolytes [32, 33], and composite reinforcement of separators [34, 35]. These approaches aim to delay the onset of thermal runaway from the root cause. However, passive protection cannot implement active intervention at the critical point of thermal runaway. Therefore, researchers have further developed active-response intelligent materials triggered by temperature, voltage, or stress signals. These include temperature-responsive materials [36, 37], voltage-responsive materials [38, 39], mechanically responsive materials [40, 41], self-healing materials [42, 43], and microencapsulated internal chemical fuse designs [44, 45]. These active-response materials can spontaneously initiate protection mechanisms upon sensing abnormal signals, enabling functions such as thermal shutdown, overcharge protection, and mechanical damage self-healing, thereby achieving precise intervention at the critical threshold of thermal runaway-representing the evolution from passive protection to active response.

At the system integration level, protection strategies have expanded from enhanced heat dissipation and thermal propagation blocking to a comprehensive layout encompassing system-level safety design and failure response. In terms of thermal management, a “heat dissipation-thermal insulation-flame retardancy” integrated thermal protection system is constructed through the synergistic application of active and passive cooling technologies such as liquid cooling, immersion cooling, phase change materials, and heat pipes, combined with thermal propagation blocking measures like aerogels, fire-retardant coatings, and structural isolation. Regarding system-level safety design, the safety logic of the battery management system (BMS) is evolving from monitoring and decision-making to active intervention warning, achieving precise protection through dynamic thresholding, hierarchical strategies, and coordinated responses [46, 47]. Mechanical designs for pressure relief, fire, and explosion prevention constitute critical lines of defense in system safety. Researchers are effectively reducing explosion risk and flame propagation during thermal runaway by optimizing pressure relief valve layout and venting capacity, designing directional flow-guiding and flame-retardant structures, and applying casing reinforcement technologies such as carbon-fiber-reinforced polymers [48, 49]. Furthermore, the influence of electrical connections and topological structures on thermal safety is receiving increasing attention. Parallel electrical topologies can significantly exacerbate thermal propagation risk due to electrothermal coupling effects, whereas thinner connectors can fuse under excessive current, interrupting hazardous energy transfer [50, 51]. Additionally, particles ejected during thermal runaway can induce arc faults, imposing new quantitative requirements on the engineering design and safety assessment of electrical clearances [52]. Through the organic integration of these multi-level, multi-dimensional protection strategies, a system-level thermal safety protection framework characterized by “internal-external synergy and multi-level defense” is gradually taking shape.

**Fig. 1:**
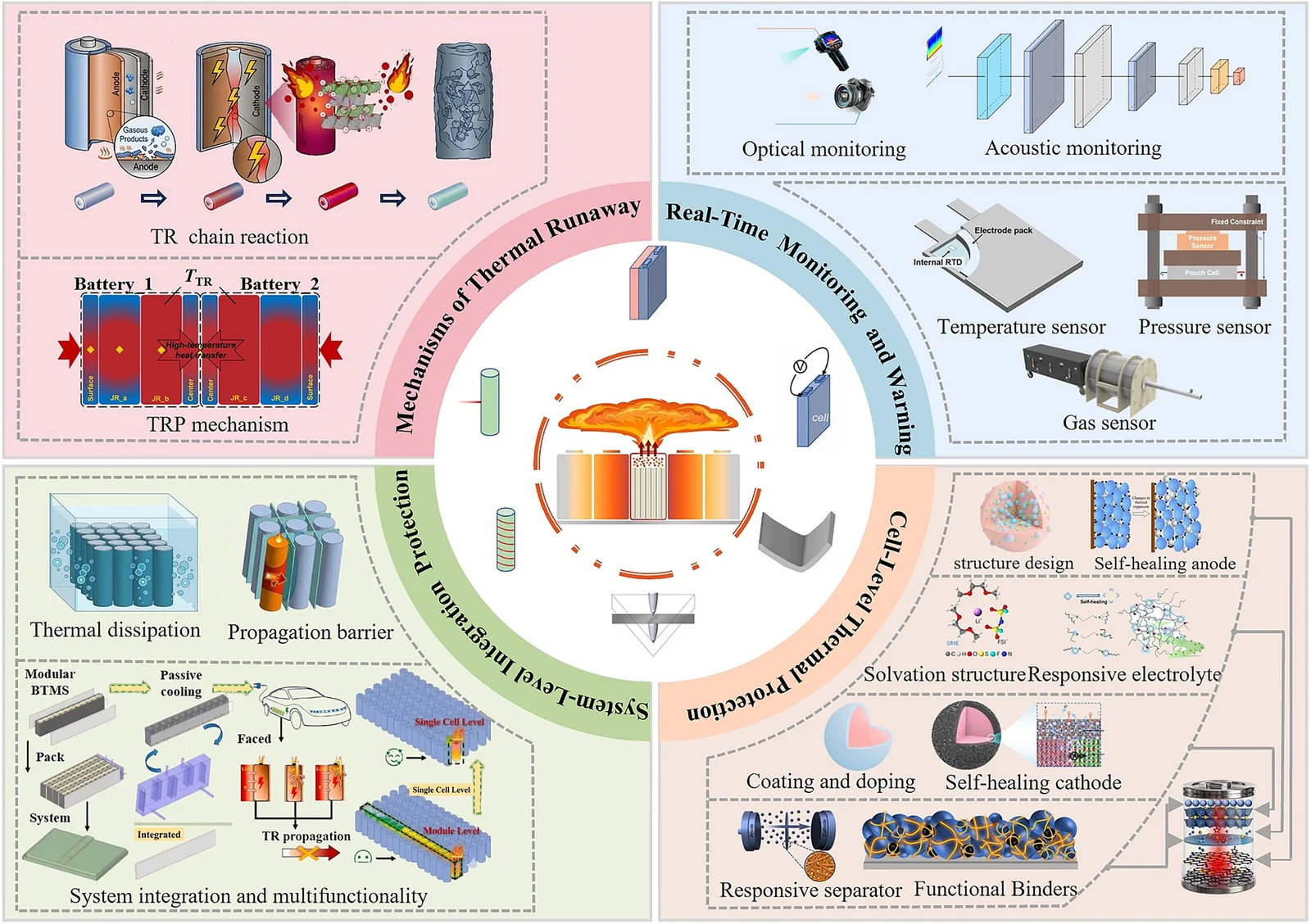
Summary schematic diagram of the battery thermal safety protection

Although significant progress has been made in existing research. Through a systematic review of the existing literature, most reviews focus on a single dimension such as thermal management, monitoring. Protection strategies remain predominantly passive, and research at the material, cell, and system levels stays fragmented. Furthermore, high-rate charging reducing thermal stability [10, 53] and thermal runaway behavior differs fundamentally across chemical systems [54]. However, few reviews propose an engineering framework capable of unifying high energy density with high safety. Consequently, what remains largely missing is a cross-scale, full-chain knowledge framework that integrates mechanism understanding, real-time sensing, and protective strategies. Additionally, few reviews have systematically revealed the paradigm evolution of intelligent materials from passive protection to active response, nor have they proposed a system-level thermal safety design philosophy that emphasizes internal-external synergy and multi-level defense. To fill these critical gaps, this review establishes a holistic “mechanism-monitoring-protection” framework, delineates the evolutionary trajectory toward active safety materials, and introduces a synergistic design philosophy for unifying high energy density with high safety. These innovations collectively move beyond component-level summaries to offer a panoramic and forward-looking perspective on battery thermal safety. Specifically, as conceptualized in Fig. 1, the content of this paper is structured across four interconnected sections that operate on a dynamic, closed-loop control logic: moving from the forward thermochemical reactions to real-time monitoring windows, which sequentially trigger cell-level material protections and system-level mitigation barriers to actively feedback and interrupt the thermal runaway cascade. This comprehensive framework is detailed throughout the following sections.

## Mechanisms of Thermal Runaway Initiation and Chain Reaction

### Triggering Conditions

Thermal runaway can be initiated by both external abuse conditions (mainly including thermal, electrical, and mechanical abuse) and spontaneous internal defects inherent to the cell. In either case, internal short circuit (ISC) serves as a critical common pathway leading to the ultimate battery failure. While external kinetic, electrical, or thermal insults represent major triggers that induce severe internal abnormalities and macro-scale ISC, spontaneous ISC frequently arises under nominal operating regimes due to latent manufacturing defects (e.g., electrode burrs and metallic contaminants) or operational degradation like lithium dendrite growth piercing the separator. To provide a structured understanding of these thermal failure mechanisms, the following sections first describe the fundamental principles of each individual abuse condition.

#### Thermal Abuse

Thermal abuse is one of the typical scenarios that trigger thermal runaway. It is primarily characterized by external heating causing the battery temperature to exceed the safe operating window. Based on the nature of the heat source and the duration of exposure, thermal abuse can be categorized into two types: instantaneous high-intensity thermal shock and chronic cumulative thermal damage. Although these two types manifest differently, both ultimately lead to thermal runaway by disrupting the thermal stability of internal materials and interfaces.

**Fig. 2:**
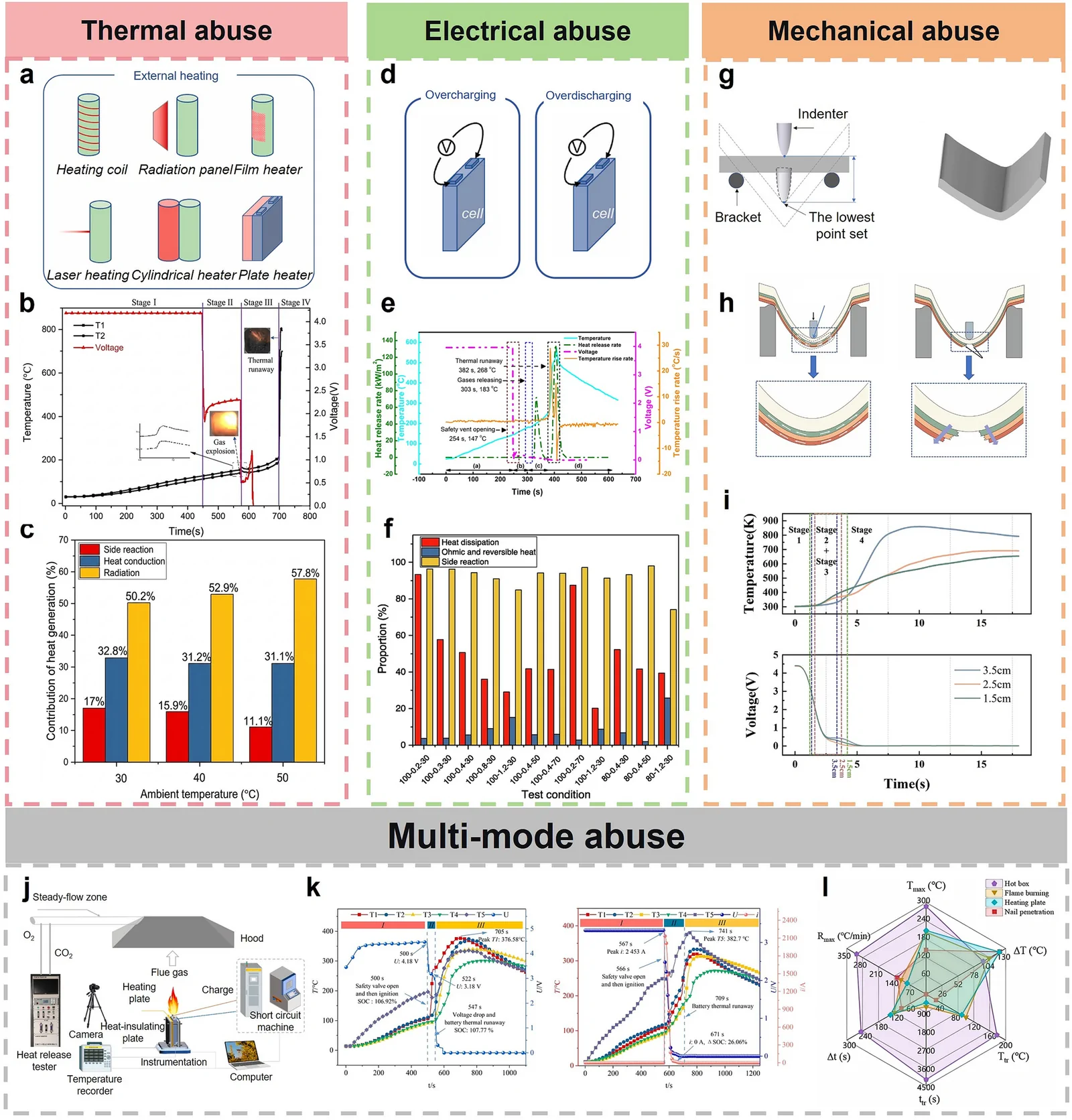
**a** Thermal abuse triggering methods. **b** Temperature and voltage changes during thermal runaway. **c** Contribution of each heat to total heat of batteries at different ambient temperature. **d** Electrical abuse triggering methods. **e** Typical curves of cell surface temperature, heat release rate, voltage, and temperature rise rate during the thermal runaway process for the cell with 75% SOC and in the absence of charging. **f** Contribution of each heat to the total heat generation. **g** Mechanical abuse triggering methods. **h** Schematic illustration of the mechanical-to-thermal failure process. **i** Corresponding curves of temperature and voltage of the battery thermal runaway under three displacement conditions. **j** Schematic view of experimental setup. **k** Temperature, voltage, and current evolution under combined thermal-overcharge abuse and short-circuit-induced safety valve opening. **l** Radar map of LFP battery TR parameters triggered by different abuse situations. Elsevier reproduced with permission from **a**, **d** Ref. [55], Elsevier; **b**, **c** Ref. [9] The Electrochemical Society; **e** Ref. [10], Elsevier; **f** Ref. [53] Elsevier; **g**–**i** Ref. [11], Elsevier; **j**, **k** Ref. [12], Elsevier; **l** Ref. [61]

Instantaneous high-intensity thermal shock is primarily caused by concentrated external heat sources [55] (Fig. 2a). Liu et al. [9] investigated the thermal runaway behavior of lithium-ion batteries in confined spaces under external heating conditions. This process can be divided into four typical stages (Fig. 2b, c): the first stage is characterized by stable surface temperature rise and voltage; in the second stage, voltage drops sharply as side reactions begin; the third stage involves gas ejection and explosion; and the fourth stage is the occurrence of thermal runaway, with temperature decreasing after reaching its peak. Thermal radiation and thermal conduction are the primary heat sources under external heating, while heat release from internal side reactions mainly occurs after thermal runaway. The heating power affects the trigger time and intensity of thermal runaway. Preheating effects are also a critical factor influencing propagation rates. Although high-power heating can rapidly trigger thermal runaway, it may weaken preheating of subsequent cells and thus slow propagation rates. In contrast, low-power heating, while prolonging trigger time, may enhance preheating and thereby promote propagation. There exists a critical heating power for thermal runaway [56]. When the heating power exceeds this threshold, the onset temperature of thermal runaway increases with higher heating power.

Laser-induced thermal runaway serves as a precise, remote, and non-invasive triggering method [57], enabling more realistic simulation of localized thermal runaway caused by internal or external hot spots in batteries. Research indicates that as laser power increases, the trigger time for thermal runaway decreases. The critical values determined through laser-induced methods are more accurate for assessing thermal hazards associated with localized hot-spot faults compared to traditional oven tests. Furthermore, the onset temperature of thermal runaway and the associated mass loss are not affected by variations in laser power. However, laser-induced methods have limitations, including surface reflection affecting efficiency and potential damage to battery structures under high power. Induction heating-based non-contact thermal abuse methods [58, 59] generate localized high temperatures by inducing eddy currents in the internal conductive layers of the battery through alternating magnetic fields, enabling high-energy-density thermal abuse within an extremely short time. The abuse mechanism involves localized current collector overheating due to induced currents, leading to separator melting and internal short circuits, ultimately evolving into thermal runaway. This method is suitable for simulating localized overheating scenarios caused by internal defects or external thermal shocks. However, the distribution of the electromagnetic field within the battery is significantly influenced by material and structural factors, making precise control of the energy distribution during thermal abuse challenging.

It is worth noting that thermal abuse is not limited to instantaneous extreme high temperatures; cumulative thermal damage resulting from long-term exposure to moderately elevated temperatures represents a common and insidious form of chronic thermal abuse [60]. Although this type of damage does not immediately trigger thermal runaway, it significantly reduces the thermal stability and mechanical safety boundaries of the battery by continuously promoting mechanisms such as SEI decomposition and regeneration, electrolyte consumption, cathode phase transitions, and microcrack propagation, thereby greatly increasing safety risks under subsequent abuse conditions. As thermal damage accumulates, elevated temperatures accelerate various side reactions in lithium-ion batteries, including loss of active materials and increased internal resistance during aging. Thermal damage leads to continuous thickening of the SEI layer on the anode and accumulation of deposits on the cathode side, resulting in increased impedance. It also reduces separator ductility and increases the likelihood of micro-short circuits, making the separator more prone to rupture and thereby increasing the risk of thermal runaway. On the cathode side, thermal damage causes particle cracking and increased surface deposits on the cathode surface, leading to active material loss and crystal structure deterioration [25].

Thermally damaged batteries exhibit a higher thermal runaway onset temperature (T_1_), indicating improved thermal stability, but a shorter thermal runaway incubation time, suggesting that although thermally damaged batteries can withstand higher temperatures, they are more prone to rapid thermal runaway once the stability limit is reached. This imposes higher demands on the design of thermal management systems for electric vehicles, which must account for the shortened reaction incubation period to prevent severe thermal runaway.

#### Electrical Abuse

Electrical abuse refers to typical scenarios in which batteries undergo charge, discharge, or overcharge processes (Fig. 2d), where current, voltage, or power exceeds the design thresholds, inducing internal side reactions and ultimately leading to thermal runaway. The key influencing factors include state of charge (SOC), charge rate, degree of overcharge, and battery aging state. These four factors are coupled with one another and collectively determine the pathway and severity of electrical abuse-induced failure.

SOC is a fundamental factor influencing thermal runaway behavior. Studies have shown that the higher the SOC, the lower the onset temperature of thermal runaway. Notably, when SOC exceeds 80%, the termination temperature of thermal runaway does not increase but rather decreases, accompanied by significant mass loss. This phenomenon indicates that high-SOC batteries undergo more intense reactions during thermal runaway; however, localized cooling due to electrolyte ejection and material expulsion may lead to a decrease in measured temperature. This reflects the inherent complexity of the thermal runaway process [56].

The charging state further amplifies the risk effect of SOC [10]. Compared with batteries in a static state, batteries under charging conditions exhibit significantly earlier safety vent opening, first gas release, and thermal runaway occurrence. Moreover, this deteriorating effect becomes more pronounced with increasing charge rates. More importantly, the initial SOC of the battery and the charge rate exhibit a synergistic risk amplification effect. The higher the initial SOC, the more significant the acceleration effect of high-rate charging on thermal runaway behavior (Fig. 2e). This indicates that when a high energy state and high-power input coexist, the thermal runaway risk faced by the battery exhibits nonlinear superposition. The underlying mechanisms are as follows: charging increases the total amount of chemical energy available for release within the battery, while rapid lithium intercalation and deintercalation in the electrode materials reduce the stability of the crystal structure, making the cathode material more susceptible to oxygen release upon decomposition at high temperatures. Additionally, the charging process renders the SEI layer on the anode dynamically unstable, making it more prone to premature decomposition and triggering exothermic chain reactions.

Overcharging is one of the primary electrical abuse modes that induce thermal runaway. Its risk is not determined by a single variable but is the result of the combined effects of charge rate, ambient temperature, and battery aging state. Quantitative studies have shown that the order of influence of these three factors is: charge rate > ambient temperature > aging [53]. High-rate charging and high temperature exacerbate lithium plating and SEI decomposition, respectively, while the stable SEI layer formed during aging may actually suppress side reactions. Figure 2f shows that the heat generated by side reactions accounts for the majority of the total accumulated heat. However, the ratio of ohmic heat to reversible heat of lithium ions increases with increasing charge rate. This can be explained by Joule’s law. As resistance decreases with increasing temperature, the ohmic heat ratio of lithium ions decrease with increasing temperature. However, due to the lower charge rate, the change is small. When the charge rate is 1.2C, the change is evident. After battery aging, internal resistance increases, and the ohmic heat ratio increases. The proportion of heat dissipation decreases with increasing charge rate and temperature. It is noteworthy that even in scenarios where low-current overcharging does not trigger thermal runaway, the battery’s heat dissipation demand is greatest, revealing the hidden thermal management stress under otherwise safe operating conditions.

Battery state of health (SOH) significantly influences the characteristics of thermal runaway triggered by overcharging. High-rate overcharging can induce a complete five-stage disaster chain, including explosion. However, the behavior of aged batteries is not simply more hazardous. Studies have found that under 1C overcharge conditions, the onset temperature of thermal runaway exhibits a non-monotonic trend, first decreasing and then increasing as SOH declines [62], challenging the conventional notion that “aged batteries are more dangerous.” Yuan et al. [63] conducted overcharge experiments on commercial LiMn_2_O_4_||graphite (Gr) batteries with different capacity retention ratios (CRR) and found that thermal runaway trigger time was negatively correlated with CRR. Mechanistic analysis revealed that with increasing aging degree, manganese oxide deposition on the cathode surface and increased lithium plating on the anode surface together leads to a reduction in the amount of reversible lithium within the battery, thereby delaying the occurrence of thermal runaway.

Commercial 18,650 batteries have a built-in overcharge protection mechanism with a clear current threshold. Research indicates that when the charging current exceeds 2.6 A (approximately 1C), thermal runaway can be triggered under heating conditions, and the onset temperature of thermal runaway decreases significantly with increasing charging current. When the charging current exceeds 10.4 A, the internal reactions become so intense that the battery undergoes explosive disintegration [56]. Yan et al. [64] further pointed out that the charge rate is the critical threshold determining the effectiveness of overcharge protection. At low rates, the current interrupt device (CID) can effectively prevent thermal runaway by cutting off the current; however, once the critical rate is exceeded, the protection mechanism fails, and the battery inevitably enters a thermal runaway state.

In summary, electrical abuse-induced thermal runaway is a multi-factor dynamically coupled process. SOC determines the battery’s energy reserve and intrinsic stability; the charge rate governs the reaction rate and interfacial stability; the degree of overcharge directly drives irreversible side reactions; and the aging state modifies material structure and interfacial properties, modulating all the aforementioned factors. Understanding the synergistic and competitive relationships among these factors forms the theoretical foundation for constructing electrical abuse protection strategies and optimizing BMS control logic.

#### Mechanical Abuse

Mechanical abuse refers to scenarios in which a battery undergoes structural deformation or damage under external forces, primarily including nail penetration, crushing, and impact. These mechanical loads induce internal short circuits, either directly or indirectly, by causing separator rupture, electrode misalignment, and current collector fracture, thereby triggering thermal runaway.

Nail penetration, as a classic test for simulating internal short circuits, involves a thermal runaway process determined by the dynamic competition between Joule heat generation and nail body heat dissipation. Studies have shown that under high SOC conditions, nails with high electrical conductivity tend to exacerbate thermal runaway due to the dominance of the Joule heating effect; conversely, under low SOC conditions, the same nail may instead mitigate thermal runaway due to the dominance of the heat dissipation effect. Furthermore, there is a regular interaction between nail diameter and battery SOC. Increasing nail diameter primarily enhances the cooling effect, thereby reducing the intensity of thermal runaway. These findings reveal the paradoxical and complex nature of thermal runaway behavior under mechanical abuse and underscore the urgency of unifying and optimizing safety testing standards [65]. In addition to nail penetration, crushing is also a typical form of mechanical abuse. Zhang et al. [11] systematically investigated the thermal runaway behavior of pouch cells under different degrees of deformation (1.5, 2.5, and 3.5 cm) through three-point bending experiments (Fig. 2g). During the bending process, electrode materials fracture and puncture the separator, leading to direct contact between the positive and negative electrodes, triggering an internal short circuit that releases substantial Joule heat and subsequently initiates exothermic chain reactions. Increased bending displacement results in more severe electrode fracture and internal short circuits (Fig. 2h), leading to higher-temperature rise rates and peak temperatures (Fig. 2i). To quantify energy and material release under such abuse conditions, Walker et al. [66] developed a large-scale fractional thermal runaway calorimetry (L-FTRC) system, providing a new method for standardized assessment of mechanical abuse consequences under semi-confined conditions.

In practical battery systems, however, the aforementioned abuse conditions often do not occur in isolation but are interrelated and coupled [12] forming complex scenarios with hazards far greater than those of individual conditions (Fig. 2j). Thermal abuse causes localized high temperatures within the battery pack, which can significantly reduce the tolerance thresholds for overcharge, external short circuits, and mechanical integrity. Compared with heating or overcharge alone, the coupled “heating + overcharge” mode can shorten thermal runaway trigger time by up to 77%, reduce trigger temperature by approximately 21%, and significantly increase the peak heat release rate, representing the highest hazard level. Similarly, the coupled “heating + short circuit” mode is also more destructive than either condition alone. The underlying reason is that high-temperature environments provide activation energy for electrochemical reactions, accelerating a series of chain reactions including dendrite growth, SEI decomposition, and electrolyte side reactions (Fig. 2k). When thermal abuse and mechanical abuse are coupled, sustained mechanical abuse under localized high-temperature conditions induced by thermal abuse can trigger secondary thermal runaway, significantly intensifying the thermal runaway severity. This is particularly relevant in large-capacity energy storage systems [67]. The thermal runaway behavior of the same battery varies significantly under different abuse conditions. In a comparative study of multi-mode thermal runaway triggering mechanisms, Gong et al. [68] conducted thermal runaway trigger experiments on commercial pouch LiFePO_4_ (LFP) batteries using nail penetration, thermal chamber, flame heating, and heating plate methods. The study showed that nail penetration triggered the most rapid response, inducing localized thermal runaway within 3 s and forming a high-temperature zone exceeding 700 °C, with subsequent internal propagation. In contrast, uniform overall heating required a temperature rise to 170 °C to trigger thermal runaway, while localized intense heating readily created localized high-temperature zones on the heated side, from which thermal runaway originated and propagated outward. Using a radar chart of thermal runaway parameters, the risks of the four abuse modes were quantitatively ranked (Fig. 2l), with the order being: nail penetration > heating plate ≥ flame heating > thermal chamber. This ranking provides important references for thermal safety design under different abuse scenarios.

In summary, thermal, electrical, and mechanical abuse conditions each compromise battery thermal stability and structural integrity through distinct pathways. Thermal abuse drives side reactions through heat accumulation; electrical abuse modulates reactivity and interfacial stability through charge state; and mechanical abuse directly induces internal short circuits through structural deformation. More importantly, in practice, these three types of conditions often occur in coupled forms, with their destructive effects exhibiting nonlinear superposition and significantly elevating the risk of thermal runaway. Recent studies have revealed that particles matter released during the thermal runaway of LIBs constitute a critical factor affecting system safety and also pose environmental concerns, yet they are seldom addressed in existing reviews. These particles primarily originate from the thermal decomposition and molten products of battery components. High-temperature, high-velocity particles can trigger fires, enhance thermal radiation, and release toxic elements, thereby threatening human health and ecological safety. Consequently, integrating these particles into a comprehensive “mechanism-sensing-protection” framework is essential for a more holistic assessment of thermal runaway hazards and for the refinement of safety standards.

### Internal Chain Reaction

**Fig. 3:**
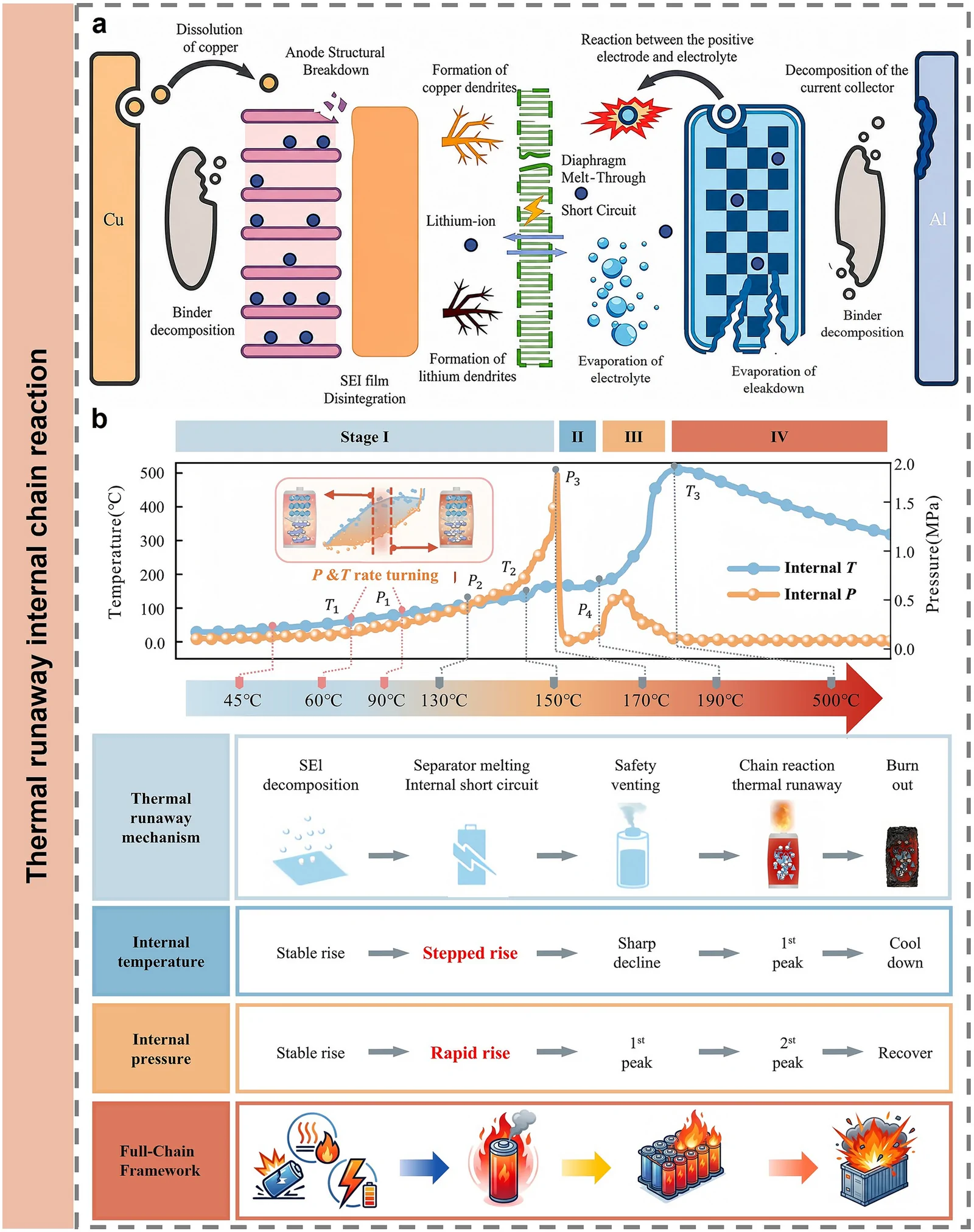
**a** Schematic diagram of thermal runaway reaction mechanism. **b** Behavioral characterization of the thermal runaway process. Elsevier reproduced with permission from **a**, **b** Ref. [8]

Once external abuse breaches the safety boundaries of a battery, a series of spontaneous and mutually reinforcing exothermic side reactions occur within the battery, ultimately leading to an exponential rise in temperature. This process is termed the thermal runaway chain reaction. The essence of thermal runaway lies in the coupling of the chain-triggering of exothermic side reactions and the thermal-temperature positive feedback within the battery. The schematic of the thermal runaway reaction mechanism is shown in Fig. 3a.

Regardless of the electrochemical system, cell capacity, or triggering method, the evolution of multi-physical signals follows a similar temporal pattern, providing a theoretical basis for thermal runaway warning based on multi-parameter fusion of temperature, pressure, voltage, and other signals. Researchers have identified three universally significant characteristic temperatures (T_1_, T_2_, T_3_) [69], which mark key nodes in the thermal runaway evolution process and have become general indicators for evaluating battery thermal safety. T_1_ corresponds to the thermal runaway onset temperature, T_2_ corresponds to thermal runaway trigger temperature, and T_3_ corresponds to the maximum thermal runaway temperature.

The internal chain reaction of thermal runaway follows a well-defined temperature-triggered sequence, with each reaction initiating sequentially and contributing cumulative heat. The energy accumulated through the chain reaction is ultimately released violently through the opening of the safety vent. This release occurs not only in the form of heat but also converts to mechanical energy through intense ejection behavior. During the core ejection phase, unignited jets with velocities up to 8.3 m s^−1^ and temperatures comparable to open flames may occur, accompanied by hazardous phenomena such as jet inclination and spark bursting. Such high-speed, high-temperature jets themselves serve as powerful ignition sources and heat transfer media [70], constituting the direct driving force for the propagation of thermal runaway to the system level. As battery capacity increases, the physical hazards of thermal runaway grow exponentially [71], potentially leading to catastrophic consequences such as the battery casing rupture or even explosion.

The entire thermal runaway process can be divided into four characteristic stages [72], and the correspondence between these stages and the internal chain reaction is illustrated in Fig. 3b. It should be noted that while Fig. 3b illustrates a generalized multi-stage evolution framework of internal pressure during TR, the specific pressure profiles and gas generation kinetics display distinct characteristics between LFP and NCM systems. For NCM batteries, the catastrophic thermal runaway is coupled with severe lattice oxygen release from the high-nickel cathode at elevated temperatures (~ 200 °C). The liberated active oxygen species violently oxidize the carbonate solvents, resulting in an explosive burst of gas (e.g., CO_2_, CO) within an extremely short timeframe. Consequently, NCM systems exhibit a sharp, aggressive peak pressure and a high pressure rise rate. Conversely, owing to the strong P-O covalent bonds within the PO_4_^3−^ tetrahedra, LFP cathodes maintain excellent thermal stability without lattice oxygen evolution. The internal pressure accumulation in LFP cells is primarily dominated by SEI decomposition, anode-electrolyte interfacial reactions, and volatile electrolyte vaporization. As a result, LFP batteries typically present a much more gradual and moderate pressure rise profile, with a substantially lower maximum peak pressure and a milder pressure rise rate compared to their NCM counterparts. Stage I is the initial self-heating phase, corresponding to a temperature range of approximately 80–130 °C. The figure shows the first increase in internal pressure, attributed to electrolyte evaporation and minor gas generation. During this stage, the SEI layer begins to decompose (at approximately 90 °C) [13], exposing fresh Gr surfaces and triggering the initial exothermic reaction between the lithiated anode and the electrolyte [14]. Voltage remains stable, and mass loss has not yet occurred, indicating that the battery is still at a critical stage of reversible damage. Stage II spans from safety vent opening to thermal runaway triggering. The figure presents the first pressure peak, corresponding to the opening of the safety vent, accompanied by the instantaneous release of large quantities of electrolyte vapor and reactive gases. Concurrently, voltage experiences its first sharp drop, signaling the occurrence of a large-scale internal short circuit. The temperature curve shows an accelerating temperature rise rate, exceeding the 130 °C threshold, at which point the separator begins to melt and shrink [15]. This stage represents a critical turning point where thermal runaway transition from an intervenable state to an uncontrollable one. Stage III is the thermal runaway eruption phase. The figure shows the temperature exponentially climbing to above 500 °C, accompanied by a second pressure peak and a sharp decline in mass. This stage corresponds to the dual-path exothermic reaction driven by cathode oxygen release. Part of the oxygen reacts with the electrolyte, releasing heat, while the majority reacts with the lithiated anode, releasing even more heat. Voltage drops to zero, marking complete battery failure. The point of maximum temperature rise rate indicated in the figure corresponds to T_2_, while the peak temperature point corresponds to T_3_. Stage IV is the cooling phase, where reactants are depleted; temperature gradually decreases, pressure subsides, and mass loss stabilizes.

It is noteworthy that the triggering of the aforementioned reaction sequence does not strictly follow a temperature-by-temperature progression. Numerical simulation studies have shown that, in contrast to the staged reactions induced by internal self-heating, when a battery is heated by an external flame, its internal SEI decomposition and side reactions between the anode/cathode and electrolyte are simultaneously triggered at a low temperature of approximately 50 °C, with comparable contributions from both anode and cathode reactions [73]. This results in a significantly lowered thermal runaway trigger temperature threshold and a sharp increase in reaction intensity, revealing the profound influence of external heat flux patterns on internal reaction kinetics. The state of charge, degree of aging, and ambient temperature of the battery significantly affect the triggering and evolution of the chain reaction [9]. Higher SOC leads to shorter thermal runaway trigger times, less total heat required, and higher thermal runaway temperatures. Lower SOH, i.e., aged batteries, exhibit earlier onset of side reactions due to lithium plating and hydrogen accumulation in Gr, leading to earlier thermal runaway. Higher ambient temperatures result in higher initial battery temperatures, faster thermal runaway occurrence, and an increased contribution of radiation heat transfer.

Thermal runaway behavior varies significantly across different electrochemical systems, fundamentally due to differences in the thermal stability and reaction pathways of key materials. Lithium metal batteries are considered next-generation energy storage systems due to their high energy density. Xu et al. [74] revealed the chain reaction sequence of thermal runaway in lithium metal pouch cells. First, an exothermic reaction occurs at approximately 77 °C between inorganic SEI components and PF_5_, a decomposition product of LiPF_6_, initiating self-heating. Subsequently, exposed fresh lithium dendrites react vigorously with the electrolyte, driving the temperature up to T_2_. Finally, oxygen release from the cathode and its reaction with the electrolyte lead to the maximum thermal runaway temperature T_3_. The thermal runaway behavior of LFP batteries exhibits multi-step reaction characteristics. Zhang et al., using three levels of analysis, revealed that the primary heat source for LFP batteries under thermal abuse is the anode-electrolyte reaction [75], which exhibits two exothermic peaks in the 200–350 °C range, corresponding to the reaction of lithiated Gr with electrolyte and the reaction of residual lithium with binder, respectively. Comparing the thermal runaway responses of LFP, NMC, and LTO systems under external heating [76], it was found that NMC batteries have the lowest thermal runaway onset temperature and release gas with a CO concentration as high as 30%, posing both flammability and high toxicity risks. Although LFP exhibits better thermal stability, it releases the highest concentration of H_2_, highlighting significant explosion risks. LTO batteries exhibit relatively lower gas-related hazards.

In-depth analysis of chain reactions heavily relies on advanced thermal analysis techniques. Accelerating rate calorimetry (ARC) enables precise tracking of overall battery temperature rise and heat release rate under adiabatic conditions, allowing reliable determination of key characteristic temperatures such as thermal runaway onset, trigger, and peak temperatures. Differential scanning calorimetry (DSC), through sample preparation that closely simulates actual battery internal compositions, enables analysis of the exothermic characteristics of individual components and their interactions at the material level, thereby revealing the microscopic chemical origins underlying macroscopic ARC curves. By establishing correlations between DSC and ARC curves [77], the micro-macro-correlation mechanisms of thermal runaway can be elucidated.

In thermal runaway research, the comparability of test results between laboratory-scale and industrial-scale batteries is critical for the reliability of mechanistic studies [78]. Comparing the differences in thermal runaway behavior between the two reveals that coin cells, due to their lower active material proportion and higher heat capacity ratio, exhibit self-heating rates insufficient to trigger a complete chain reaction. Their thermal runaway intensity and maximum temperature are significantly lower than those of industrial batteries. The ratio of battery capacity to heat capacity can serve as a key indicator for evaluating scale comparability; batteries with capacities greater than 1 Ah exhibit thermal runaway behavior comparable to that of industrial batteries.

In summary, the internal chain reaction of thermal runaway is a multiscale cascading process spanning from materials to systems. This process is modulated by multiple factors, including the battery’s electrochemical system, state of charge, aging state, and external heat flux pattern, ultimately converting chemical energy into thermal and mechanical energy through high-speed venting, propagating to the system level.

The thermal runaway behavior of a battery is fundamentally determined by the thermal stability of its key materials [69]. The following sections will systematically elucidate the roles and interaction mechanisms of the core materials, specifically the anode, cathode, and electrolyte, in the chain reaction, laying the theoretical foundation for designing intrinsically safe batteries from a material perspective.

#### Anode

Under abuse conditions, the anode side undergoes a series of exothermic processes, including SEI thermal decomposition, reactions between the lithiated anode and electrolyte, and lithium dendrite-induced interfacial side reactions, constituting the initiation phase of the thermal runaway chain reaction.

The anode is the starting point of the thermal runaway chain reaction. The decomposition of the SEI layer on its surface is generally considered the initial source of heat generation in thermal runaway. The SEI is a passivation layer formed on the anode surface through the reductive decomposition of the electrolyte, primarily composed of organic components (e.g., lithium methyl carbonate, LMC; lithium ethylene monocarbonate, LEMC) and inorganic components (e.g., Li_2_CO_3_, LiF). Under normal operating conditions, it serves a protective function but becomes unstable at elevated temperatures. When the battery temperature rises to 90–120 °C, the metastable organic components within the SEI begin to decompose, releasing heat while exposing the fresh Gr anode surface. This exothermic reaction marks the formal initiation of the thermal runaway chain reaction. The thermal stability of the SEI layer directly determines the T_1_ and is a key parameter for evaluating the intrinsic thermal safety of a battery.

The thermal decomposition behavior of the SEI is closely related to its composition. Studies have shown that LMC decomposition initiates with the cleavage of the O-CH_3_ bond, producing CH_3_ radicals, followed by the progressive removal of CH_3_, CO_3_, and other groups, ultimately converting to Li_2_CO_3_; the entire process is endothermic. LEMC decomposition involves proton transfer reactions triggered by O-H bond cleavage, with significant mass loss occurring at approximately 150 °C, generating LiCO_3_H and ethylene glycol (EG), and ultimately also converting to Li_2_CO_3_. Li_2_CO_3_ itself possesses high thermal stability and remains almost undecomposed when present alone below 400 °C.

**Fig. 4:**
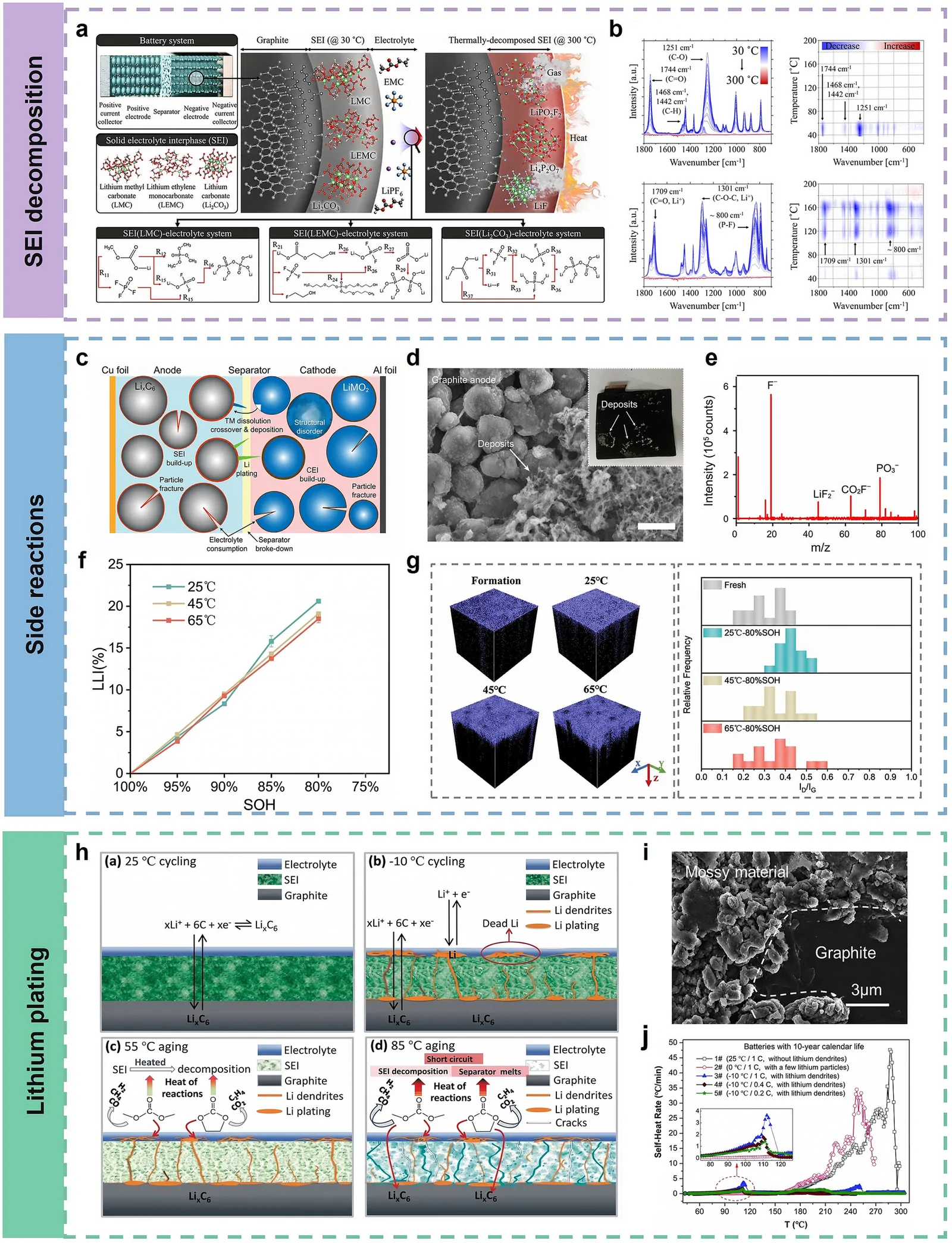
**a** Schematic diagram of the fundamental reaction pathways of key intermediate and final products formed within the anode-electrolyte system. **b** Comparison of evaporation trends between pure EMC and the electrolyte with EMC and 3 M LiPF_6_. **c** An overview of various battery degradation mechanisms. **d** SEM image and photograph of the Gr anode from the disassembled NCA-Gr cell. Deposits were observed at the anode surface. The scale bar is 10 µm. **e** TOF-SIMS spectra collected from the surface of the Gr anode. **f** Quantified loss of lithium inventory (LLI) in LFP ||Gr batteries cycled at 25, 45, and 65 °C. **g** Analysis of spatial inhomogeneity in anodes. **h** Schematic diagram of the mechanism for lithium dendrites promoting gassing and heat generation. **i** Optical photos and SEM images of the anode morphology. **j** Thermal runaway curves of the batteries with 100% SOC after cycling 50 times under different conditions. Elsevier reproduced with permission from **a**, **b** Ref. [79], Wiley; **c**–**e** Ref. [81], Wiley; **f**, **g** Ref. [80], The Royal Society of Chemistry; **h**–**j** Ref. [83]

However, in practical battery systems, SEI components do not exist in isolation but interact with the lithium salt in the electrolyte. Kim et al. [79] revealed the exothermic reaction pathways between the SEI and the electrolyte system during the initial stage of thermal runaway (Fig. 4a). Specifically, SEI components first react with PF_5_, the thermal decomposition product of LiPF_6_, to form POF_3_; POF_3_ further reacts with the SEI to produce lithium fluorophosphates, which ultimately decompose into pyrophosphates. Comparing the evaporation behavior of pure EMC and EMC with 3 M LiPF_6_ revealed that Li^+^ coordination significantly alters the desorption characteristics of EMC (Fig. 4b). Mechanistically, this distinct shift in the thermal desorption profiles provides deep insights into the micro-level interfacial reaction kinetics. In the absence of lithium salt, pure EMC molecules easily vaporize and desorb at relatively low temperatures due to weak intermolecular forces. Upon the introduction of LiPF_6_ strong ion-dipole interactions establish structured Li^+^-EMC coordination spheres, which significantly increases the activation energy barrier for EMC vaporization, thereby shifting the desorption peak toward a higher temperature regime. This physical suppression of premature solvent volatilization directly modulates the early-stage interfacial side reactions. The localized depletion of volatile free linear carbonate solvents at the electrode-electrolyte interface heavily restricts the concentration of highly reactive organic species available for radical-initiated thermal decomposition. Consequently, this solvation locking effect successfully hinders early-stage gas accumulation and decelerates the self-enhancing exothermic parasitic reactions between the volatilized solvents and the reactive electrode sites, effectively buffering the onset of catastrophic thermal runaway. The study shows that when SEI components exist alone, they undergo endothermic decomposition primarily above 150 °C; however, when mixed with the electrolyte, significant exothermic peaks and mass loss occur before 100 °C, with Li_2_CO_3_ exhibiting the highest exothermic magnitude. Paradoxically, although Li_2_CO_3_ is widely recognized as a thermally stable ingredient within the isolated SEI layer, its aggressive exothermic reactivity when blended with the electrolyte stems from a distinct cross-species Lewis acid-base reaction. Specifically, the strong Lewis acid PF_5_, generated from the premature thermal dissociation of LiPF_6_, vigorously attacks the alkaline Li_2_CO_3_ phase. This interfacial chemical crossover facilitates a highly exothermal pathway (Li_2_CO_3_ + PF_5_ → 2LiF + POF_3_ + CO_2_ ↑), bypassing the high thermal activation energy barrier required for the independent decomposition of pure Li_2_CO_3_. Furthermore, the heavily liberated POF_3_ and localized acidic species aggressively propagate the subsequent catalytic ring-opening and exothermic decomposition of the volatile carbonate solvents. Therefore, the prominent chemical basicity of Li_2_CO_3_ intrinsically renders it the most vulnerable site for attacking salt-derived chemical fragments, thereby dominating the catastrophic heat generation below 100 °C. Since a CO_2_ environment promotes the formation of Li_2_CO_3_, exposure to CO_2_ should be avoided during battery manufacturing. Notably, the exothermic interaction between the SEI and the salt to produce POF_3_ plays a crucial role in the early stages of thermal runaway. This work elucidates at the molecular level the mechanism by which the SEI-electrolyte system initiates initial self-heating below 100 °C, providing important guidance for battery safety design. Regarding the electrolyte system, LEMC generated from EC-based electrolytes reacts with PF_5_ at relatively low temperatures, while LMC generated from EMC-based electrolytes releases greater heat, indicating significant differences in thermal runaway risk modes between the two. After SEI decomposition exposes the fresh Gr surface, the lithiated anode (LiC_6_) undergoes a vigorous exothermic reaction with the electrolyte at 120–200 °C, constituting the main exothermic stage of thermal runaway development [80]. This stage marks the critical transition from “slow self-heating” to “uncontrolled escalation” in temperature rise.

In terms of quantitative analysis of SEI growth and evolution, Zhang et al. [80] systematically quantified the aging mechanisms of LFP||Gr batteries at different temperatures, revealing that SEI growth, rupture, and repair processes constitute the primary mechanism leading to battery aging. Elevated temperatures significantly accelerate the aging process, with lithium inventory loss being the main contributor to capacity loss, and its proportion increases with temperature (Fig. 4f). At high temperatures, organic SEI components decompose and transform into more stable inorganic components. Spatial inhomogeneity in SEI growth at high temperatures forms a self-reinforcing cycle, accelerating battery aging (Fig. 4g). Furthermore, depletion of the VC additive shifts electrolyte decomposition behavior from additive-dominated to solvent- and salt-dominated, altering SEI composition and structure and ultimately leading to rollover failure. Regarding the mechanical failure induced by excessive SEI growth during deep aging, Qiao et al. [81] first revealed the failure mechanism in which excessive SEI growth during deep aging leads to battery swelling and rupture. As aging deepens, the SEI on the anode surface continues to thicken. Excessive SEI growth generates significant stress between electrodes, causing cathode densification and thinning, as well as separator compression and porosity reduction. More importantly, SEI components such as F and P penetrate into the separator, blocking ion transport channels. This mechanical stress ultimately leads to stress concentration at the electrode head region, causing the metal casing to rupture along the electrode head direction and triggering short-circuit failures.

Beyond SEI decomposition, active lithium precipitated on the anode surface is another important factor exacerbating thermal runaway risk. Lithium plating and dendrites, owing to their high reactivity and interfacial penetration capability, significantly lower reaction trigger temperatures and accelerate interfacial side reaction processes.

High-rate charging leads to excessively low anode potential, causing lithium ions to deposit in metallic form on the Gr surface, forming moss-like lithium metal [82] (Fig. 4i). This precipitated active lithium undergoes vigorous exothermic reactions with the electrolyte at approximately 110 °C, which can directly trigger thermal runaway without the involvement of internal short circuits. This reaction significantly reduces the T_2_ from 215.5 to 103.9 °C, substantially lowering the thermal safety boundary of the battery. Wang et al. [83] investigated long-calendar-life (10 years) LFP||Gr pouch cells and found that after high-rate cycling at -10 °C, extensive lithium dendrites appeared on the Gr anode surface, with dendrite density increasing significantly with higher current (Fig. 4h). Lithium dendrites reduced the T_1_ from approximately 80 °C to about 50 °C, while the thermal runaway temperature decreased substantially from 170 °C to approximately 100 °C (Fig. 4j). Mechanistic analysis indicated that lithium dendrites physically penetrate the SEI layer, trigger interfacial side reactions, and accelerate electrolyte decomposition and gas generation, forming a vicious cycle of “dendrite growth-interfacial heat release-internal gas generation-thermal runaway.” Wang et al. [81] first revealed the thermodynamic driving mechanism for lithium deposition at high temperatures (Fig. 4c). Dendritic lithium deposits were observed on the anode surface of failed batteries, and TOF-SIMS analysis detected characteristic fragment ions such as F^−^, LiF^−^, CO_2_F^−^, and PO_3_^−^, confirming that these deposits are metallic lithium and its reaction by-products with the electrolyte (Fig. 4d, e). Lithium deposition at high temperatures is triggered by temperature hotspots generated by non-uniform lithium-ion flux; when a temperature difference of approximately 10 K exists on the Gr anode surface, lithium deposition may become thermodynamically more favorable than lithium intercalation into Gr in the high-temperature region. Severe lithium plating on the anode surface is the primary cause of capacity rollover. The inherent lithium dendrite issue in lithium metal batteries (LMBs) makes thermal safety challenges even more severe [74]. The accumulation of lithium dendrites and dead lithium significantly reduces battery thermal stability. Suppressing dendrite growth and reducing the reactivity between the lithium metal anode and electrolyte at high temperatures are effective strategies for improving the safety performance of LMBs.

Wang et al. [84] proposed that the lithium oxidation reaction is the most harmful trigger for battery thermal runaway. The study found that even in the absence of any liquid electrolyte, solid-state batteries still undergo severe thermal runaway under separator cracking conditions, with lower ignition temperatures and faster rates. Inserting an O_2_ blocking layer between the cathode and separator completely suppressed ignition, confirming the critical role of blocking oxygen crosstalk. The study further demonstrated that even with Gr anodes, fully lithiated Gr (LiC_6_) exhibits high reactivity, and the lithium oxidation reaction remains the dominant mechanism of thermal runaway.

Long-term cycling aging profoundly reshapes the thermal response characteristics of the anode at a deeper level. Galushkin et al. [85] revealed that Gr anodes accumulate atomic hydrogen during long-term cycling. When the temperature continues to rise to 30–120 °C, these hydrogen atoms undergo a vigorous recombination reaction to form hydrogen gas, releasing substantial heat. Notably, this reaction is independent of the battery’s state of charge and represents an independent release channel for chemical energy accumulated during cycling aging, serving as the primary heat source in the first stage of thermal runaway. Zhang et al. [86] compared the effects of different aging modes on anode thermal stability and found that lithium plating and dendrite growth induced by low-temperature cycling aging represent the most hazardous aging mode. Such aged batteries exhibited a T_1_ approximately 20 °C lower than that of fresh batteries, with significantly shortened thermal runaway delay times. The root cause is that precipitated active metallic lithium undergoes vigorous exothermic reactions with the electrolyte at relatively low temperatures and is more prone to causing internal short circuits, greatly lowering the thermal runaway trigger threshold. In contrast, long-term room-temperature cycling aging, which primarily leads to SEI thickening, has a relatively minor impact on thermal runaway characteristic temperatures.

Understanding the degradation mechanisms and interfacial behavior of anode materials under different operating conditions provides the theoretical foundation for designing intrinsically safe batteries from a material perspective and for optimizing battery management system control strategies.

#### Cathode

As one of the core components of lithium-ion batteries, the thermal stability of the cathode material is directly related to the overall safety performance of the battery. Under abnormal conditions such as thermal abuse and electrical abuse, the cathode material is prone to decomposition reactions, releasing active oxygen and undergoing vigorous exothermic reactions with the electrolyte, leading to a sharp rise in internal temperature and subsequently triggering thermal runaway. Furthermore, instability at the cathode-electrolyte interface exacerbates side reactions and localized overheating, further increasing the risk of thermal runaway. A deep understanding of the thermal degradation mechanisms of cathode materials provides the theoretical foundation for designing intrinsically safe batteries from a material perspective.

Nickel-rich layered oxides (NCM, NCA, Ni ≥ 80%) have become mainstream for high-energy lithium-ion batteries due to their high specific capacity [87]. However, they also present significant thermal safety risks. Their thermal stability exhibits a negative correlation with nickel content; as nickel content increases, the decomposition temperature, T_1_, and T_2_ of the cathode material all show a decreasing trend [88].

**Fig. 5:**
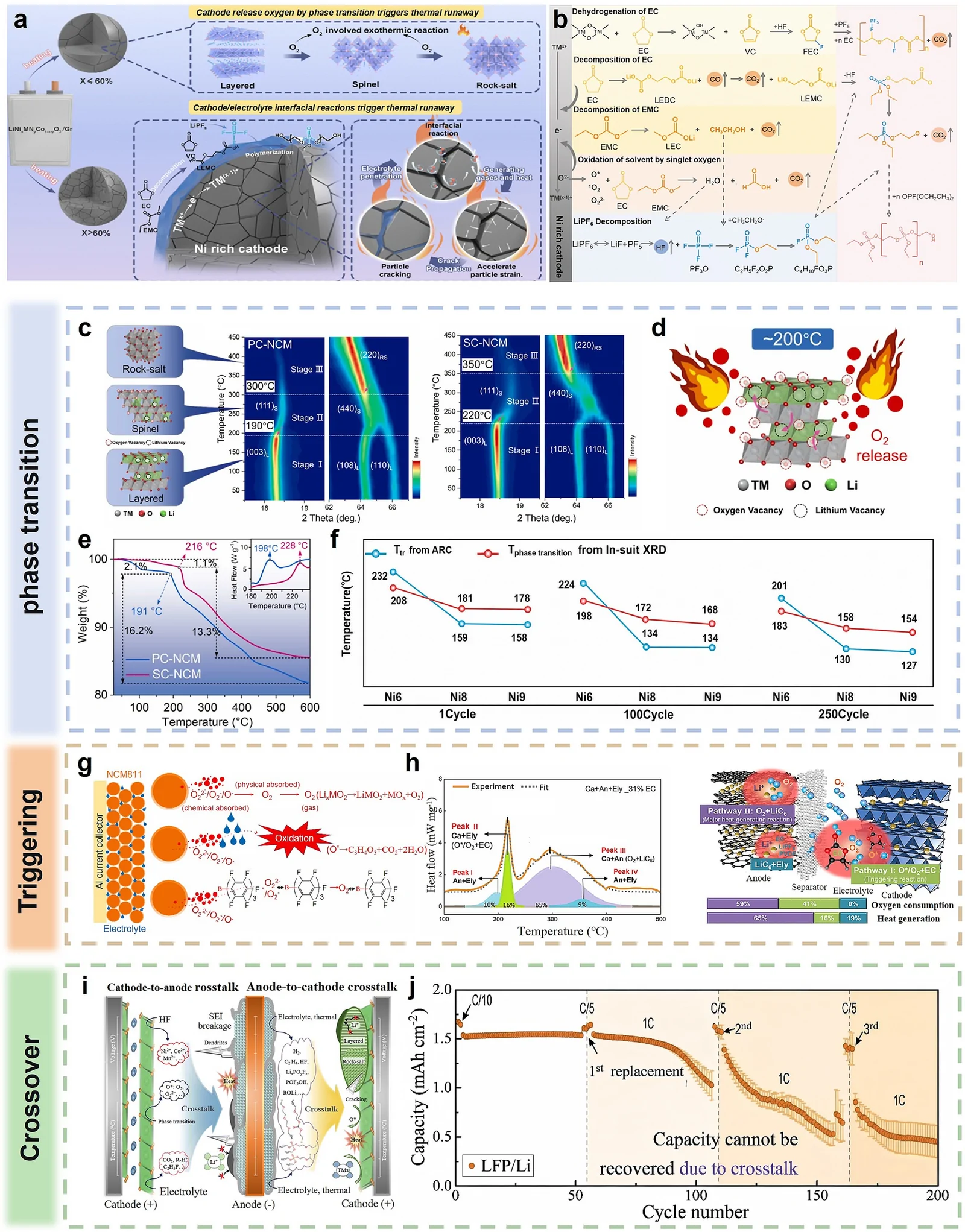
**a** Schematic illustration of thermal runaway triggering mechanisms in NCM materials with different Ni-contents. **b** Interphase chemistry of the NCM cathode and electrolyte at elevated temperatures. The bulk phase transition and oxygen release process. The contour plots of the in situ H-XRD patterns for the (003) and (108), (110) diffraction peak fading during heating of **c** PC-NCM and SC-NCM. **d** Illustration of the dangerous bulk thermal degradation temperature at around 200 °C and **e** TGA curves. **f** Comparison between the phase transition temperatures of cathode materials and thermal runaway temperatures of the NR-NCM batteries. **g** Transformation process of oxygen species into oxygen gas of charged cathode in the presence of electrolyte and in the presence of TPFPB. **h** Two key oxygen evolution reaction pathways during the thermal runaway of the NMC811||Gr cell. **i** Illustration of the origins of bidirectional crosstalk in LIBs. **j** Capacity retention of an LFP cathode when replacing the Li anode every 50 cycles. Adapted with permission. The Royal Society of Chemistry; **a**, **b**, **f** Ref. [90], Wiley; **c**–**e** Ref. [89], Elsevier; **g** Ref. [91], Elsevier; **h** Ref. [94], The Royal Society of Chemistry; **i** Ref. [18], American Chemical Society; **j** Ref. [93]

The phase transition and oxygen release that occur in nickel-rich layered cathodes upon heating are the chemical origins of thermal runaway [89]. The active oxygen released from the cathode material serves as the ignition source for the thermal runaway chain reaction, playing a role throughout all three stages of thermal runaway. T_1_ is primarily dominated by the exothermic reaction between the anode and the electrolyte, accumulating initial heat for thermal runaway. As temperature rises, the reaction between the cathode and electrolyte further accelerates self-heating, becoming a key reaction for triggering thermal runaway at T_2_. In the final thermal runaway stage, the reaction between the cathode and the anode (i.e., the vigorous exothermic reaction between oxygen and the lithiated anode) provides the dominant heat contribution. The cathode material is not merely a participant in thermal runaway but also the igniter and accelerator of the chain reaction. As shown in Fig. 5c, the characteristic peaks of the layered structure in polycrystalline materials begin to significantly decay at approximately 190 °C, indicating a transition from the layered structure to a spinel structure, marking the onset of oxygen release, accompanied by the reduction of Ni^3+^/Ni^4+^ and the formation of oxygen vacancies (Fig. 5d). As temperature increases further, the spinel structure transforms into a disordered rock-salt structure, leading to complete collapse of the crystal structure. The mass loss observed in TGA primarily originates from the release of lattice oxygen, accompanying the layered-to-spinel transition and the cleavage of peroxide bonds to generate oxygen gas (Fig. 5e).

Traditional views generally attribute thermal runaway primarily to bulk lattice oxygen release at high temperatures. However, recent studies through systematic comparisons of systems with different nickel contents have revealed fundamental differences in the dominant mechanisms [90]. For Ni6 (60%) batteries, thermal runaway occurs after cathode phase transition, whereas for Ni8 (80%) and Ni9 (90%) batteries, thermal runaway occurs before cathode phase transition (Fig. 5f). This comparison clearly reveals that for medium- and high-nickel (Ni ≤ 60%) cathodes, bulk oxygen release is the primary trigger, while for ultrahigh-nickel (Ni ≥ 80%) cathodes, parasitic exothermic reactions at the cathode-electrolyte interface are the dominant mechanism of thermal runaway (Fig. 5a).

Oxygen release from nickel-rich cathodes is not a simple gas evolution but rather a profound electrochemical-structural coupling process [91]. Li et al. [92] demonstrated that the reaction between oxygen species released from the cathode and the flammable electrolyte is the direct trigger for thermal runaway. Charged cathodes release highly active oxygen species at approximately 200 °C [93], and the thermally driven oxygen species undergo vigorous exothermic reactions with the surrounding flammable electrolyte (Fig. 5g). Jiang et al. [90] systematically revealed the complete chemical picture of the cathode-electrolyte interface reaction (Fig. 5b). High-valence Ni^4+^ activates lattice O^2−^ into reactive oxygen, which directly oxidizes EC/EMC to generate CO_2_ and H_2_O. EC simultaneously undergoes dehydrogenation to form VC/FEC or ring-opening reduction to form LEDC/LEMC with release of CO/CO_2_ H_2_O triggers LiPF_6_ hydrolysis, producing LiF deposits and the strong Lewis acid PF_3_, which catalyzes the formation of organophosphate oligomers and fluorinated polymers. This work deconstructs the interfacial reaction into clear pathways, providing targets for interface regulation.

Hou et al. [94] quantitatively resolved the two key reaction pathways of endogenous oxygen during thermal runaway and their thermal contributions through experimental design. In NMC811||Gr batteries, approximately 41% of oxygen reacts with ethylene carbonate (EC) in the electrolyte, contributing 16% of the total heat. This reaction is key to triggering and accelerating the self-heating process, and EC in turn accelerates the phase transition of the cathode, forming a positive feedback loop. Approximately 59% of oxygen diffuses to the anode and reacts with Gr (LiC_6_), contributing 65% of the total heat (Fig. 5h). This reaction is the source of the peak temperature and primary destructive force of thermal runaway. This mechanism clearly explains why thermal runaway, once triggered, is difficult to contain-because it is an endogenous self-sustaining reaction; isolating the battery from the external environment cannot cut off the exothermic chemical reactions within the battery. The intensity of thermal runaway in nickel-rich batteries increases with nickel content precisely because higher nickel content lowers the oxygen release temperature, making these two exothermic reactions more prone to coupling and superposition. Researchers have compared the thermal degradation mechanisms and oxygen release kinetics of single-crystal and polycrystalline nickel-rich cathode materials, finding that grain boundaries serve as key pathways for rapid oxygen release in polycrystalline materials. Song et al. [89] performed localized structural diagnostics on samples heat-treated at 200 °C using FIB-STEM and EELS, revealing the microscopic pathways of oxygen release. Strain analysis showed that grain boundary regions in polycrystalline materials are in a tensile strain state, with the strain direction aligned with the direction of oxygen release, providing a sustained driving force for rapid oxygen escape through grain boundaries. In contrast, single-crystal materials exhibited a beneficial strain gradient, with internal compressive stress suppressing bulk oxygen diffusion. This finding reveals the correspondence between mechanical stress and oxygen release during thermal decomposition, offering new insights for regulating cathode thermal stability through morphology engineering.

The pathway and intensity of the thermal runaway chain reaction strongly depend on the characteristics of the cathode material and exhibit a trade-off between internal thermal runaway and external combustion/explosion. For nickel-rich ternary cathodes, their strong oxidizing power leads to vigorous oxygen release during thermal runaway, causing energy to be violently released within the battery, but simultaneously consuming the electrolyte and generating less combustible gas, thus resulting in lower external combustion risk. Compared with layered transition-metal oxides, LFP exhibits a distinctly altered thermochemical chain reaction profile, which strictly aligns with the evolutionary sequence. Due to the intrinsic thermodynamic stability of the strong P-O covalent bonds within the olivine framework [95], the structural catastrophic collapse of LFP is significantly postponed to elevated temperature regimes (typically exceeding 250 °C), accompanied by minimal lattice oxygen evolution. Consequently, the global thermal runaway propagation in LFP-based cells is primarily propelled by the persistent accumulation of anode interfacial side reactions and the subsequent bulk electrolyte decomposition, bypassing the aggressive cathode-mediated solvent oxidation stage that dominates high-nickel systems. This distinctive sequence explains the significantly prolonged induction period and lower peak heat-release rate observed during LFP thermal failure. Therefore, the evaluation and protection of battery thermal safety require precise, differentiated design of electrochemical systems based on an understanding of the cathode chemistry [54].

Traditional thermal runaway chain reaction models have focused on internal decay or internal short circuits of a single electrode. However, in high-energy-density batteries, electrode crosstalk tightly couples the failure pathways of the cathode and anode, forming a cross-electrode accelerating failure network [17, 18]. This crosstalk is bidirectional. Transition-metal ions, reactive oxygen species, and electrolyte oxidation products released from the cathode migrate to the anode surface, catalyzing SEI decomposition and inducing lithium dendrite growth. Conversely, gases and soluble organic species generated from electrolyte reduction at the anode diffuse to the cathode, potentially accelerating transition-metal dissolution and, upon encountering oxidizing species released from the cathode at high temperatures, triggering vigorous exothermic reactions. Even in the absence of a significant internal short circuit, crosstalk can directly trigger thermal runaway (Fig. 5i). Lee et al. [93] investigated LFP cathode failure induced by crosstalk from the lithium anode. The study found that after replacing the cycled lithium anode with a fresh lithium anode, capacity fading could not be fully recovered (Fig. 5j). This phenomenon is closely related to changes in electrolyte composition due to reduction at the lithium anode and the appearance of soluble fluorinated by-products (HF, CH_x_F_y_, and POF_x_(OH)_y_) and carboxyl compounds. These soluble species crosstalk with the cathode side, leading to severe deterioration. Therefore, inhibiting crosstalk can be approached from two directions: suppressing the generation of crosstalk species through electrode material modification, and blocking the migration of crosstalk species through electrolyte additives and functional separators.

In summary, the thermal stability of the cathode material is the core factor determining the overall thermal safety of the battery. Thermal runaway in nickel-rich cathodes is a multi-stage process originating from oxygen release through electrochemical-structural coupling, developing via dual-path exothermic reactions, accelerated by grain boundary engineering and interfacial parasitic reactions, and ultimately forming a system-level failure network through crosstalk. Optimizing cathode materials and regulating their interfacial compatibility are key aspects for improving the thermal safety of lithium-ion batteries.

#### Electrolyte

In lithium-ion batteries, the chemical and electrochemical behavior of the electrolyte plays a critical role in triggering and exacerbating the thermal runaway chain reaction. Conventional organic liquid electrolytes are prone to decomposition, vaporization, and even combustion under abuse conditions such as high temperature or short circuit, releasing substantial heat and generating flammable gases, directly driving temperature rise and pressure accumulation. Concurrently, the repeated rupture and reconstruction of the unstable solid-electrolyte interphase/cathode-electrolyte interphase (SEI/CEI) layers at the electrode-electrolyte interfaces not only cause capacity fade but also continuously release reaction heat, forming a positive feedback loop coupling “thermal-chemical-electrical” processes within the battery and significantly accelerating the thermal runaway process. In recent years, with the development of solid-state batteries, the thermal runaway mechanisms of solid electrolytes have also garnered increasing attention.

**Fig. 6:**
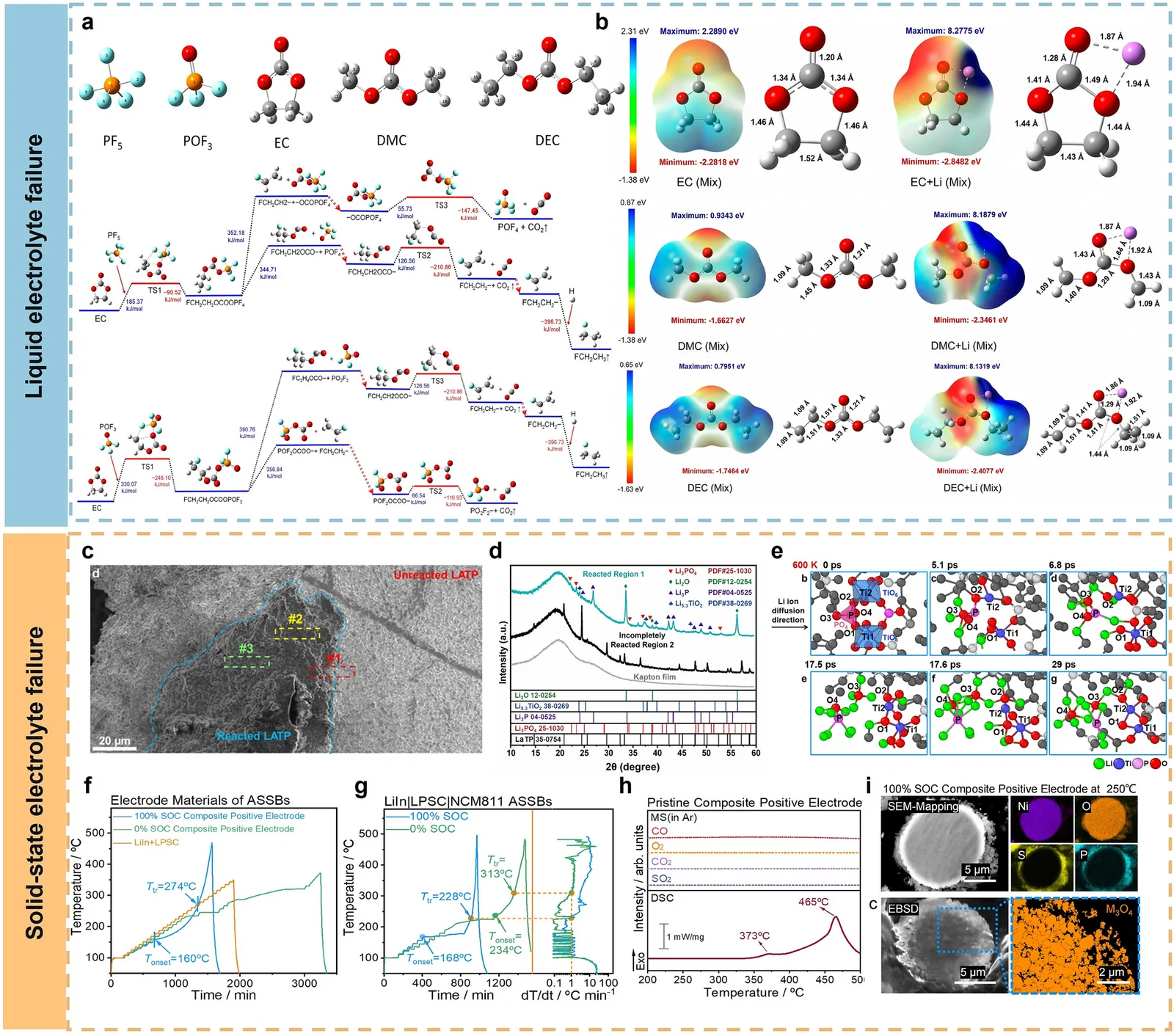
**a** Configurations of main constituents of the electrolyte and the reaction pathways of EC with PF_5_ and POF_3_ in the electrolyte environment. **b** Changes in electrostatic potential distribution and molecular structure before and after binding EC, dimethyl carbonate (DMC), and DEC with Li^+^. **c** SEM image of the interface between the reacted and unreacted regions. **d** XRD patterns of different regions after reaction between metallic Li and LATP. **e** Time-sequential snapshots of DBMD simulations on the interface structure of Li and LATP, respectively. Thermal runaway characteristics of ASSBs and thermal analysis of the pristine composite positive electrode. **f** HWS curves of ASSBs at 100% and 0% SOC; **g** HWS curves of the electrolyte with electrodes collected from ASSBs at 100% and 0% SOC; **h** DSC and MS curves of the pristine composite positive electrode (NMC811, LPSC, and VGCF), with inset showing SEM and elemental mapping images at 25, 400, and 500 °C. **i** Cross-sectional SEM with elemental mapping and EBSD maps of the 100% SOC composite positive electrode at 250 °C. Reproduced under the Creative Commons CC BY license, © 2025 by the authors from **a** Ref. [32], © 2024 by the authors; **b** Ref. [96], American Chemical Society; **c**–**e** Ref. [97], © 2026 by the authors; **f**–**i** Ref. [98]

The decomposition behavior of the electrolyte during thermal runaway is extremely complex, influenced not only by its own components but also closely related to lithium salt decomposition products and ionic environments. In recent years, quantum chemical calculations have provided powerful tools for revealing the underlying microscopic mechanisms. Tian et al. [32] computationally demonstrated that PF_5_ and POF_5_, as Lewis acids generated from the thermal decomposition of LiPF_6_, can significantly catalyze the decomposition of carbonate solvents (EC, gaseous DMC, DEC). The mechanism involves the binding of P atoms in PF_5_ and POF_3_ with ether oxygen atoms, while F atoms bind with adjacent C atoms, promoting ring-opening or chain cleavage of solvent molecules (Fig. 6a). Furthermore, another study by the same team further revealed the catalytic effect of Li^+^ on electrolyte decomposition [96]. Upon binding with EC and DMC, Li^+^ can alter the molecular electrostatic potential distribution, lowering the energy barrier for decomposition reactions and accelerating the generation of flammable gases (Fig. 6b). This discovery reveals the catalytic role of cations in electrolyte thermal decomposition, providing a new perspective for understanding the thermal stability of electrolytes.

In solid electrolyte systems, the thermal runaway mechanism fundamentally differs from that of liquid systems, primarily manifesting as severe solid-solid interfacial reactions dominated by structural collapse. Yan et al. [97] employed multiscale in situ characterization to reveal the failure process at the interface between the NASICON-type solid electrolyte Li_1.3_Al_0.3_Ti_1.7_P_3_O_12_ (LATP) and metallic lithium. The study showed that under high-temperature conditions, lithium diffuses into the LATP lattice, causing the collapse of its corner-sharing PO_4_-TiO_6_ structure, leading to amorphization and the gradual precipitation of nanocrystalline phases such as Li_3_PO_4_, LiP, and Li_0.5_TiO_2_ (Fig. 6c, d). These interfacial reactions release substantial heat, triggering severe thermal runaway. Combined with density functional theory molecular dynamics (DFT-MD) simulations, the complete evolution process of ion diffusion, bond breaking, and structural collapse was revealed at the atomic scale (Fig. 6e). This finding challenges the simplistic notion of solid electrolytes being intrinsically safe, demonstrating that severe reactions at solid-solid interfaces at elevated temperatures can also lead to catastrophic thermal runaway.

Wu et al. [98] systematically investigated the two-stage thermal runaway mechanism of the NCM811||LPSC system using the sulfide electrolyte LPSC (Li_6_PS_5_Cl). The study found that both fully charged and fully discharged cells underwent severe thermal runaway (Fig. 6f), indicating that the intrinsic thermal stability of materials does not equate to overall battery safety. Thermal analysis of disassembled components confirmed that the interfacial reaction between the cathode and the electrolyte is the primary trigger for thermal runaway (Fig. 6g). Even without electrochemical cycling, NCM811 and LPSC still undergo vigorous reactions under thermal driving, demonstrating intrinsic thermodynamic incompatibility between them (Fig. 6h). The reaction pathway was elucidated at the microscopic scale: at 250 °C, S does not enter the NCM811 bulk phase, only forming a spinel phase on the surface; at 400 °C, substantial S enters the NCM811 particles, forming interfacial products such as NiS, Li_2_SO_4_, and Li_3_PO_4_. By synthesizing electrochemical decomposition products and mixing them with delithiated NCM811, it was confirmed that species such as -S-S-, -P-S-P-, and Li_3_PS_4_ are the root causes of early-stage heat release and SO_2_ generation (Fig. 6i).

During thermal runaway, electrolyte decomposition not only causes fire and explosion but also generates complex chemical toxic secondary hazards. Cutting-edge research at the vehicle level has shown that thermal runaway releases submicron inhalable particulate matter at concentrations as high as 1.41 × 10^6^ particles/L^−1^ [19]. Of these particles, 78.1% originate from electrolyte decomposition, with the remainder derived from electrode materials, and they contain highly toxic components such as Li^+^ and CN^−^. Inhalation poses significant long-term health risks.

In summary, liquid electrolytes accelerate solvent decomposition through the catalytic effects of LiPF_6_ decomposition products and Li^+^, releasing large amounts of heat and flammable gases. Although solid electrolytes do not present liquid leakage risks, structural collapse at solid-solid interfaces can also trigger vigorous exothermic reactions. Understanding the decomposition mechanisms and catalytic pathways of electrolytes in different systems provides the theoretical foundation for enhancing intrinsic battery safety through electrolyte material design and also offers scientific insights for early thermal runaway warning and post-accident management.

To establish a clear diagnostic perspective on battery safety design, deciphering the quantitative proportions of heat contributions from individual components is highly essential. Although the precise enthalpy values are inherently variable depending on the specific cell chemistries, unified benchmarks from micro-calorimetry investigations (e.g., DSC and ARC) on high-energy ternary lithium-ion systems reveal a distinct heat-distribution paradigm. Typically, the early-stage anode-related interfacial side reactions-encompassing SEI structural degradation and the subsequent parasitic reactions of lithiated graphite (LiC_6_) with the electrolyte—contribute approximately 15%–30% of the total internal chemical heat [99]. Despite their relatively moderate heat enthalpy, these reactions govern the initial self-heating stage and serve as the thermal trigger for subsequent aggressive pathways. Progressing into elevated temperature regimes (> 180 °C), the catastrophic structural collapse of the cathode coupled with severe lattice oxygen evolution emerges as the dominant internal heat source, yielding roughly 30%–50% of the total thermal budget [100]. Simultaneously, the core thermal decomposition of bulk electrolyte ingredients, including LiPF_6_ dissociation and solvent radical-mediated polymerization, accounts for 25–40% of the internal heat generation [101]. It is worth noting that if gas venting occurs and triggers external solvent combustion with ambient oxygen, the external flaming heat output can exponentially surpass the internal reaction heat, shifting the global thermal hazard dominance to the external environment.

#### Separator

**Fig. 7:**
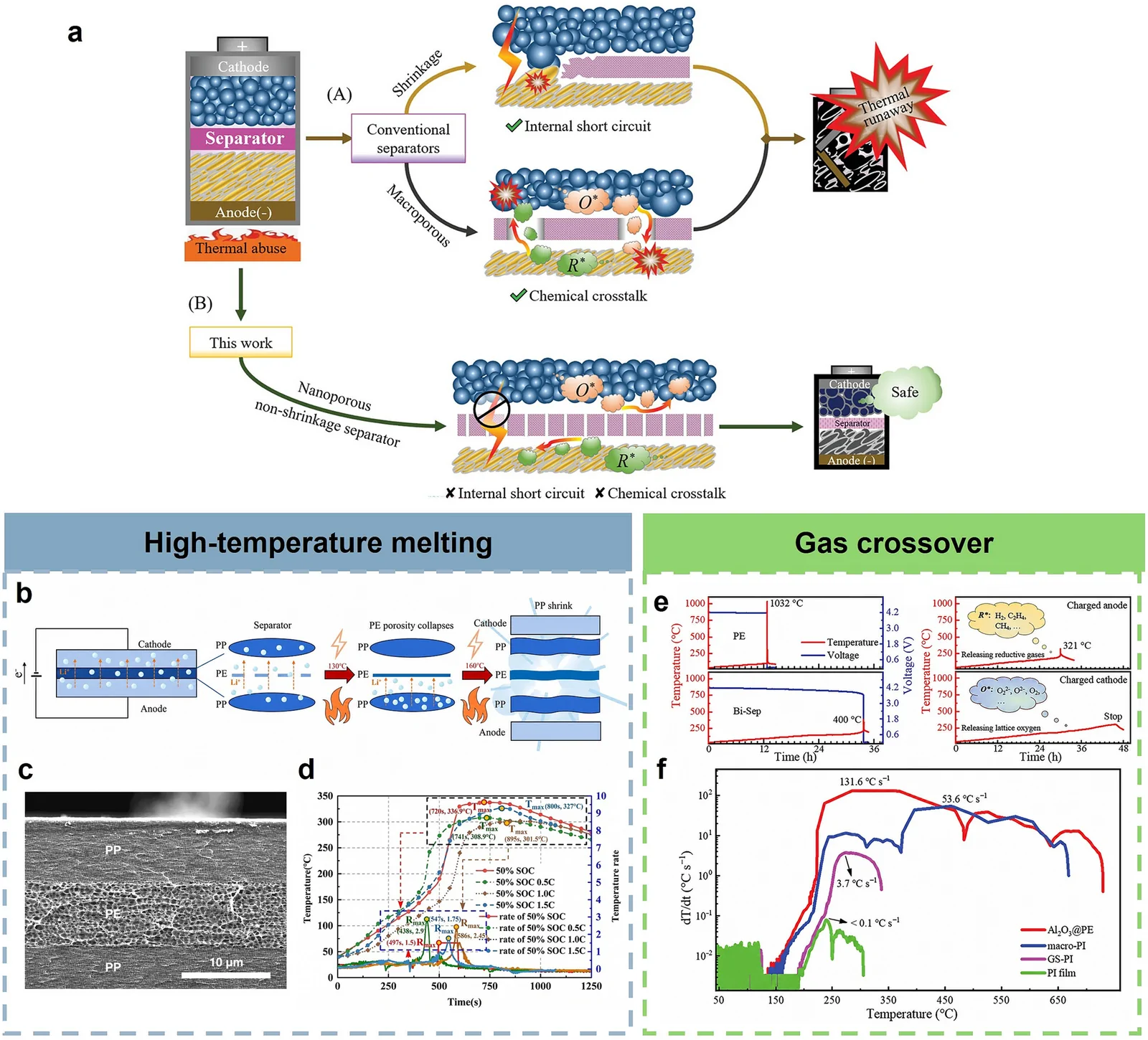
**a** (A) Thermal safety challenges of conventional separators (thermal shrinkage and chemical crosstalk) and the mitigation strategy using a non-shrinkage, crosstalk-free nanoporous separator. **a** (B) Microscopic changes in diaphragm closed pore and TR within the battery in this study. **b** Coupled mechanism of TR in batteries under charging and heating conditions. **c** Cross-sectional scanning electron micrographs of PP-PE-PP three-layer membrane. **d** Battery TR temperature and temperature rise rate comparison chart. **e** Temperature/voltage profiles of the cell using different separators and thermal degradation of the charged anode and cathode in the presence of electrolyte. **f** Temperature dependence of dT/dt profiles of the fully charged 1 Ah cells with different separators based on ARC. Wiley reproduced with permission from **a**, **f** Ref. [103], Elsevier; **b**–**d**, Ref. [102], Wiley; **e** Ref. [80]

As the physical barrier between the positive and negative electrodes and the ion transport channel within the battery, separator failure is a critical link in the thermal runaway chain reaction. Polyolefin microporous membranes, owing to their low cost, ease of production, and strong processing adaptability, are currently the most widely used separator materials. A typical structure consists of a three-layer PP-PE-PP configuration [102] (Fig. 7c). The low-melting-point PE layer can melt and close pores below the thermal runaway temperature, increasing the resistance of the electrolyte filled in the separator, while the high-melting-point PP layers maintain mechanical integrity to prevent short circuits between the electrodes.

Separator failure leads to thermal runaway through two key pathways [103], first, internal short circuits induced by thermal shrinkage, generating enormous Joule heat and triggering vigorous reactions between the positive and negative electrodes; second, the inability of the macroporous structure to block chemical crosstalk between reactive oxygen released from the cathode and reducing gases generated from the anode. This framework provides an intuitive understanding of the central role of the separator in thermal runaway (Fig. 7a).

Conventional understanding has focused on large-area internal short circuits caused by overall melting or shrinkage of the separator at high temperatures. However, recent studies [102] have revealed that under coupled thermal abuse and charging conditions, the separator may undergo a more complex progressive failure process of “pore closure-blocking-breakdown”. When external heating is applied under charging conditions, the low-melting-point PE layer in the PP/PE/PP composite membrane melts first, closing micropores and blocking lithium-ion transport channels. With continued external charging current, battery impedance surges and voltage rise sharply, exacerbating side reactions such as electrolyte decomposition and further accelerating temperature rise. Ultimately, when the temperature exceeds the melting point of the PP layers, the mechanical integrity of the separator is destroyed, triggering a violent internal short circuit and thermal runaway (Fig. 7b). Notably, the timing of separator pore closure is significantly influenced by the initial state of charge of the battery. At 50% SOC, pore closure occurs before thermal runaway, with voltage exhibiting a single sudden rise. At 75% SOC, pore closure intertwines with localized thermal runaway, resulting in a characteristic double voltage drop. At 100% SOC, intense reactions cause pore closure and internal short circuit to occur almost simultaneously (Fig. 7d). This finding directly correlates abnormal voltage signals with the microscopic failure process of the separator.

Addressing thermal shrinkage alone is insufficient to prevent thermal runaway. Studies have shown [104] that even with a Bi-Sep separator that does not shrink at high temperatures, thermal runaway can still occur if the separator pore size is too large to block gas crosstalk (Fig. 7e). The root cause is that oxygen released from the cathode at high temperatures passes through the separator and undergoes vigorous redox reactions with lithiated Gr at the anode, releasing substantial heat. This finding clearly confirms that both thermal shrinkage and chemical crosstalk must be blocked to fundamentally cut off the propagation path of thermal runaway.

Comparative studies of thermal behavior of separators with different pore sizes have revealed the pore-size dependence of chemical crosstalk (Fig. 7f). A battery using an Al_2_O_3_@PE separator exhibited a temperature rise rate as high as 131.6 °C s^−1^ at 287 °C. A macroporous PI separator with a pore size of 600 nm, despite lacking thermal shrinkage, still underwent thermal runaway. In contrast, a GS-PI separator with an average pore size of 46.7 nm reduced the temperature rise rate to only 3.7 °C s^−1^, while a non-porous PI membrane completely suppressed thermal runaway. This pore-size-dependent effect indicates the existence of a critical pore size; when the separator pore size is smaller than this critical value, active gas crosstalk can be effectively blocked.

Thermal shrinkage and chemical crosstalk do not occur independently but are coupled, synergistically accelerating the thermal runaway process. First, internal short circuits caused by localized separator shrinkage release substantial Joule heat, sharply raising battery temperature. This temperature increase accelerates oxygen release from the cathode and gas generation from the anode, increasing the concentration of crosstalk species. Heat released by crosstalk reactions further exacerbates separator shrinkage, forming a self-sustaining vicious cycle of “thermal shrinkage-internal short circuit-crosstalk heat release-further shrinkage.”

In large-format batteries in particular, thermal runaway does not occur instantaneously throughout the entire cell but propagates spatially in the form of a flame front [105]. Localized separator failure often serves as the starting point for thermal runaway propagation. When the separator in one region of the battery undergoes pore closure or breakdown due to localized high temperature, an internal short circuit occurs in that region, releasing Joule heat. This heat conducts to surrounding areas, triggering cascading separator failure in adjacent regions and forming a propagating thermal runaway process. Understanding the physical mechanisms of this propagation process is crucial for assessing the overall thermal safety risk of battery systems. The material properties and structural design of the separator, along with the cell geometry and thermal boundary conditions, collectively determine the speed and pathway of thermal runaway propagation.

In summary, separator failure in thermal runaway results from the combined effects of two mechanisms: internal short circuits induced by thermal shrinkage and the inability of the porous structure to block chemical crosstalk. Understanding these failure mechanisms provides important guidance for designing novel separators with both thermal shrinkage resistance and crosstalk blocking functionality.

#### Thermal Safety Characteristics of Next-Generation Battery Systems

To provide a comprehensive blueprint for next-generation energy storage safety, Table 1 visually benchmarks the critical thermal safety parameters and chemical failure hallmarks across LIBs, sodium-ion batteries (SIBs), and all-solid-state batteries (ASSBs). Derived from a consolidated synthesis of recent multiscale calorimetric metrics and in situ spectroscopic profiles, these electrochemical paradigms exhibit vastly divergent thermochemical evolutionary trajectories.

**Table 1:** Comparison of key thermal safety parameters and failure mechanisms of LIBs, SIBs, and ASSBs

Comparison dimension	Liquid lithium-ion batteries (LIBs)	Sodium-ion batteries (SIBs)	All-solid-state batteries (ASSBs)
Thermal runaway onset temperature (T_1_)	~ 90 °C (NCM system)	94–120 °C (SOC-dependent)	175–302 °C (electrolyte-dependent)
Thermal runaway trigger temperature (T_2_)	~ 205 °C	~ 192 °C	150–350 °C
Peak temperature (T_3_)	~ 695–800 °C	~ 236–480 °C	~ 400–750 °C
Maximum self-heating rate	Relatively low	Moderate (~ 114 °C s^−1^)	Up to 19,652 °C min^−1^
Primary trigger mechanism	SEI decomposition → anode-electrolyte reaction	Anode sodium cluster-driven early exotherm → cathode oxygen release crosstalk	Solid-solid interfacial thermolysis + oxygen release (oxide) or self-decomposition (sulfide)
Key failure characteristics	Separator thermal shrinkage, cathode oxygen release	Quasi-metallic sodium clusters, cathode oxygen release, electrode crosstalk	Interphase nature, moisture sensitivity, dendrite penetration
Major heat source contribution	SEI decomposition + anode-electrolyte reaction	Anode sodium cluster exotherm (early) + cathode oxygen release crosstalk (major)	SE/Li interface decomposition + cathode oxygen release + electrode crosstalk
Gas evolution characteristics	H_2_, CO, CO_2_, hydrocarbons	High gas generation (~ 5.3 L/Ah), high CO/CO_2_ ratio, lower H_2_ proportion	H_2_S, SO_2_, CO_2_ (sulfide system)
Explosion risk	Moderate	Wide explosion limit range, expands with SOC	Highly toxic gases, requires directional venting
Environmental sensitivity	Moisture-sensitive (HF generation)	Humidity-sensitive, layered cathodes hygroscopic	Sulfides extremely moisture-sensitive (< 0.5% RH requirement)
Major safety challenges	Flammable electrolyte, rapid thermal propagation	Low early exotherm temperature, surge in gas generation	Interfacial thermochemical instability, moisture-induced thermal runaway
Core protection strategies	Separator optimization, flame-retardant additives, thermal management	Sodium cluster stabilization, inorganic-rich SEI, ether-based electrolytes, gas management	Interfacial engineering (high-entropy SEI/dual-conductive coating), stack pressure regulation, humidity control

##### Mechanism Origin of Thermal Runaway in Sodium-Ion Batteries

Deviating from conventional LIBs whose TR is primarily initiated by SEI decomposition, SIBs demonstrate a unique crosstalk failure pathway characterized by “anode intrinsic triggering, acute cathode acceleration, and an interfacial gas surge.”

The anode serves as the early-stage inception site for thermal runaway in SIBs. Through multiscale equivalent analysis, Niu et al. [106] unraveled that the “quasi-metallic sodium clusters” localized within the closed nanopores of hard carbon (HC) under routine sodiation act as the predominant trigger. At 100% SOC, the T_1_ of NaV_3_(PO_4_)_3_ (NVP)/HC pouch cells is as low as 94 °C. Among the three distinctive environments of sodium residing in hard carbon (ionic sodium, sodium clusters, and metallic sodium), the quasi-metallic sodium clusters exclusively materialize at the low-potential plateau region under high SOC states, presenting an electronic density of states at the Fermi level substantially higher than that of bulk metallic sodium. This exceptionally high chemical reactivity enables them to bypass the confinement of the SEI film and prematurely trigger the decomposition of linear carbonates at approximately 92 °C, generating oligomers and dominating the initial exothermic stage of TR.

Furthermore, the carbonization temperature of hard carbon exerts a deterministic influence on its thermal stability. Low-temperature carbonized HC (~ 1000 °C) abounds with structural defects and dangling bonds, which catalytically accelerate electrolyte decomposition. Conversely, high-temperature carbonized HC (~ 1900 °C), despite suppressing interfacial degradation via defect elimination, promotes the segregation of free metallic sodium, which is highly prone to triggering violent exothermic explosions under moisture-leakage or other abuse conditions [107]. In comparison, alloy-type anodes experience massive volumetric swelling during sodiation, yielding repetitive fracture and regeneration of the interfacial passivation films, thereby posing a cumulative thermal risk significantly exceeding that of intercalation-type HC counterparts.

When the internal cell temperature escalates beyond approximately 180 °C, cathode lattice failure becomes the core driving force accelerating the catastrophic deterioration of TR [107]. Owing to the larger ionic radius of Na^+^, the transition-metal-oxygen (TM-O) bond energy in sodium-based layered oxides is intrinsically weaker than the Li-O coordination in conventional LIBs. Consequently, the oxygen-release onset temperature of sodium layered cathodes (~ 180 °C) shifts significantly lower than that of lithium-based configurations (~ 230 °C). Under elevated temperatures or deep desodiation states, layered cathodes [108] are highly susceptible to severe phase transitions accompanied by lattice oxygen outgassing. The released highly active oxygen species rapidly diffuse via crosstalk to the anode site, engaging in severe chain-propagating exothermic reactions with the residual sodiated species or sodium clusters.

The thermal failure dynamics of diverse cathode systems display pronounced structural dependency [109]. Layered oxides, driven by the severe coupling of “phase transformation and oxygen evolution,” manifest the most violent thermal events. Tunnel-type oxides are primarily governed by the Jahn-Teller effect of Mn^3+^ and redox disproportionation reactions. Prussian blue analogs, despite possessing rapid ion-conducting channels, suffer from structural defects and interstitial water, rendering them prone to thermal decomposition at 150–300 °C with the release of toxic hydrogen cyanide (HCN) gas. In sharp contrast, polyanion compounds deliver the most robust thermal stability by virtue of their sturdy strongly covalent polyanionic frameworks, maintaining structural integrity even beyond 500 °C; their major thermal risks stem from the thermal degradation of the CEI rather than bulk structure collapse. Gan et al. [108] further elucidated the microscopic degradation mechanism of layered cathodes under high-voltage cycling, revealing that long-term high-potential operation significantly broadens the lattice spacing and intensifies microcracking. This synergy between cumulative lattice strain and oxygen evolution drastically amplifies the total exothermic enthalpy and prematurely advances the heat-release temperature.

The compositional design of the electrolyte directly governs the thermochemical stability of the electrode-electrolyte interfaces. Investigations indicate that ether-based electrolytes tend to construct thin, uniform, and inorganic-rich (e.g., NaF, PO_x_F_y_) passivation films on the electrode surfaces. This inorganic-enriched interface exhibits exceptional thermal passivation properties, effectively isolating the highly reactive sodiated anodes, thereby significantly retarding the self-heating kinetics of uncycled Sn-based and HC anodes. Thermal safety phase diagrams confirm that a composite anode with approximately 50% HC content delivers the optimal safety window within ether-based systems [110]. On the contrary, conventional carbonate ester-based electrolytes, despite their superior ionic conductivities, generate SEI/CEI films heavily dominated by organic components (e.g., ROCO_2_Na, RCOONa). These organic-rich layers are structurally non-uniform and mechanically fragile, undergoing violent decomposition under low thermal stresses of 100–150 °C. Regarding sodium salts, NaTFSI stands out due to the high oxidative resistance of its anionic skeleton, showing an initial thermal decomposition threshold and thermal stability markedly superior to those of the weakly coordinated NaPF_6_, although it carries an intrinsic pitting corrosion risk toward the aluminum current collector under severe abuse states.

At the full-cell level, SIBs display unique macroscopic heat generation and high-pressure outgassing profiles. Zhang et al. [111] for the first time systematically quantified the heat generation characteristics of P2-Na_0.8_Ni_0.33_Mn_0.67_O_2_//HC full cells, verifying that the cathode reversible heat contribution plays a dominant role. The total heat output escalates with increasing current density and decreases with rising operational temperature. At low temperatures and high currents, irreversible heat dominates the profile, and aged cells—burdened by elevated internal resistance—yield substantially higher thermal power than fresh counterparts. In terms of gas evolution and venting behavior, large-format SIBs exhibit severe multi-dimensional high-pressure outgassing and high-intensity jetting dynamics during TR. At 100% SOC, although the T_3_ of SIBs (~ 236.6 °C) is lower than that of LFP cells (~ 306.3 °C) due to the lower total exothermic enthalpy of the sodium cathode, its T_2_ (~ 167.7 °C) is noticeably lower. Crucially, driven by the violent decomposition of ester/ether solvation shells catalyzed by the hard carbon interface and residual active sodium, the specific gas generation per ampere-hour of SIBs is approximately 3.6-fold higher than that of LFP, expanding nonlinearly with SOC. Under identical SOC conditions, the CO/CO_2_ ratio of vented gas from SIBs is 19.53% higher than that of LFP, and the explosion limit range widens significantly as the SOC increases. This indicates that while emerging SIB architectures mitigate high-intensity flame risks, the high-pressure venting and secondary explosion hazards induced by abrupt gas expansion constitute their unique safety bottlenecks.

In summary, the thermal runaway of sodium-ion batteries follows a distinctive “anode-triggered, cathode-accelerated” trajectory: the hard carbon sodium clusters initiate early-stage exotherms at 92–120 °C, and the cathode lattice oxygen release above 180 °C drives crosstalk reactions that catastrophically worsen the TR process. Consequently, thermal safety design must be concurrently advanced across three levels: anode interfacial regulation, cathode structural stabilization, and electrolyte optimization. Future endeavors should deeply integrate advanced in situ characterization techniques with multiscale thermal analytical databases to map quantitative correlations between material structures and characteristic thermal runaway thresholds, thereby providing a rational theoretical framework for the design of high-safety SIBs.

##### Thermal Safety Challenges of All-Solid-State Batteries

Contradicting the conventional paradigm that “solid-state electrolytes are non-flammable and thus absolutely safe,” recent pioneering studies have unmasked unique and severe thermal failure mechanisms inherent to ASSBs. The safety liabilities have not been eliminated; rather, they have shifted to the thermochemical instability of the rigid solid-solid interfaces.

Hasan et al. [112] systematically scrutinized the thermo-electrochemical stability of Li_3_PS_4_ (LPS) sulfide solid electrolytes under varied relative humidity (RH) conditions. They revealed that even a trace moisture exposure of 0.5% RH severely degrades the ionic conductivity by 13%, while a 5% RH exposure incurs a 39% loss in ionic conductivity. Concurrently, the T_1_ drops abruptly from 241 to 131 °C, and the thermal incubation window (T_1_-T_2_) is drastically compressed from 112 °C to a narrow 26 °C, which is rationalized by the hydrolysis-induced evolution of highly thermally active species such as H_2_S, LiOH, Li_2_CO_3_, and Li_2_SO_4_. In Li|LPS|Li symmetric configurations, the samples subjected to 5% RH display extreme thermal runaway behavior, characterized by a compressed T_1_-T_2_ interval of a mere 1 °C and a surging maximum self-heating rate of 2931 °C min^−1^, compared to a baseline of only 8 °C min^−1^ under pristine dry conditions-representing an escalation of nearly three orders of magnitude.

The intrinsic classification of the interphase plays a deterministic role in governing the TR kinetics of ASSBs. Vishnugopi et al. [113] discovered that LPS and LPSCl construct a kinetically self-limiting interphase against lithium metal, which is ionically conductive but electronically insulating, thereby sustaining its thermal stability post-electrochemical cycling. Conversely, Li_4_SnS_4_ (LSPS) drives the continuous expansion of a mixed conducting interphase (MCI) owing to the dynamic segregation of metallic Sn during cycling. Even uncycled LSPS exhibits a prominent self-heating rate of 196.3 °C min^−1^, and upon cycling, its T_1_ plummets from 291 to 177 °C while the self-heating rate violently surges to 19,652 °C min^−1^, yielding a massive thermal spike near the melting point of lithium. At the full-cell scale, the coupling between cathode oxygen evolution and anode interfacial degradation establishes a severe “electrode crosstalk” effect. Consequently, the total heat release of compiled ASSBs is over threefold higher than that of conventional liquid LIBs, with T_3_ of ~ 750 °C that far surpasses the ~ 323 °C observed in liquid configurations.

The thermal stabilities of diverse solid electrolyte frameworks exhibit sharp disparities. Chen et al. [114] systematically quantified the thermal stabilities of four archetypal oxide solid electrolytes in contact with lithium metal, pinpointing that the root cause of TR lies in the high-temperature oxygen outgassing of the oxide frameworks and their subsequent violent reactions with molten lithium. ARC assessments indicate that Li_1.5_Al_0.5_Ge_1.5_(PO_4_)_3_ (LAGP)/Li and Li_1.3_A_0.3_Ti_1.7_(PO_4_)_3_ (LATP)/Li frameworks undergo catastrophic TR, Li_0.34_La_0.56_TiO_3_ (LLTO)/Li displays mild self-heating, whereas Li_7_La_3_Zr_2_O_12_ (LLZO)/Li exhibits no detectable heat generation, indexing a thermal stability sequence of LLZO > LLTO > LATP > LAGP. Wu et al. [115] evaluated the interfacial thermal stability of five sulfide solid electrolytes against lithium metal, illuminating a critical contradiction between material-level and interface-level thermal stabilities. Despite its superior ambient air stability, Li_4_SnS_4_ reacts violently with lithium at 200–300 °C due to the formation of highly reactive Li-Sn alloys. Conversely, Li_7_P_3_S_11_, despite possessing the lowest material-level thermal stability, releases sulfur that reacts with lithium to yield a dense Li_2_S passivation tier, which effectively impedes the subsequent thermal decomposition kinetics. This research classifies sulfide SE/Li thermal decomposition into three distinct paradigms: thermodynamically stable with a dense passivation interphase (LPSCl), thermodynamically metastable but kinetically hindered (Li_7_P_3_S_11_), and thermodynamically stable yet accelerated by reactive interphase transition (Li_4_SnS_4_). This underscores that interfacial thermal safety benchmarking must simultaneously evaluate the thermodynamic driving forces of both the bulk SE/Li and the SE/interphase boundaries.

Targeting polymer-based solid-state configurations, Zhang et al. [116] fabricated an LFP/SSE/ModLi cell through a dual in situ approach and systematically investigated its thermal characteristics. C80 microcalorimetric analysis reveals that succinonitrile (SN)-based solid electrolytes delay the initial exothermic onset by 34 °C compared to liquid counterpart electrolytes, and the heat output of the ModLi + SSE framework is reduced to a mere 9.9% of the baseline Li + SSE combination. ARC evaluations show that the self-heating onset and TR trigger temperatures for the LFP/SSE/ModLi cell are 175 and 212 °C, respectively, both considerably higher than those of liquid LIBs (145.7 and 180.1 °C). Furthermore, because the modified protective architecture isolates oxygen ingress post-rupture, the peak temperature is confined to 401 °C, vastly lower than the 1102 °C recorded for unmodified SSBs. Under non-uniform thermal abuse, the temporal window spanning from TR triggering to peak temperature for the SSB is 131 s—3.44 times longer than the 38 s of liquid LIBs—thereby offering a significantly expanded window for fire suppression.

Synthesizing the aforementioned multi-dimensional insights, the thermal runaway of ASSBs can be conceptualized as follows: high-temperature oxygen release or autolytic decomposition of the solid electrolyte yields highly active intermediates that undergo severe exothermic reactions with molten lithium; the chemical nature of the compiled interfacial phase fundamentally dictates the kinetic progression of failure. Although ASSBs offer a significantly higher self-heating onset threshold than liquid LIBs, their self-heating kinetics and peak temperatures can be far more extreme once triggered, mandating the reinforcement of stringent thermal isolation strategies at the module level.

### Modeling and Prediction Methods for Thermal Runaway

To achieve accurate prediction and effective protection against thermal runaway, mathematical models serve as powerful quantitative tools. Modeling methodologies for thermal runaway and its propagation have evolved into a spectrum ranging from detailed reaction kinetics-based mechanistic models to simplified models for system design, and integrating cutting-edge directions such as multiphysics coupling, multiscale analysis, and artificial intelligence fusion to enhance model accuracy and efficiency.

#### Models

Based on a deep understanding of thermal runaway mechanisms, constructing accurate and efficient simulation models is key to quantifying risks and predicting evolutionary pathways. In recent years, multiphysics coupling models have become the mainstream framework for describing the complex nonlinearity of thermal runaway. Mathematical modeling of thermal runaway aims to quantitatively describe the entire process from internal chemical reactions to thermal runaway. Depending on the physical processes described and modeling scales, models can be categorized into thermal models, electrical models, mechanical models, fluid dynamics models, thermo-electro-chemical coupling models, propagation theory models, and data-driven models.

##### Reaction Kinetics Models

Reaction kinetics models form the foundation of thermal runaway modeling, describing the rates and heat release of various exothermic side reactions within the battery. Early Arrhenius-type models simplified thermal runaway into a single overall reaction; although computationally simple, they cannot capture the staged characteristics of the chain reaction.

**Fig. 8:**
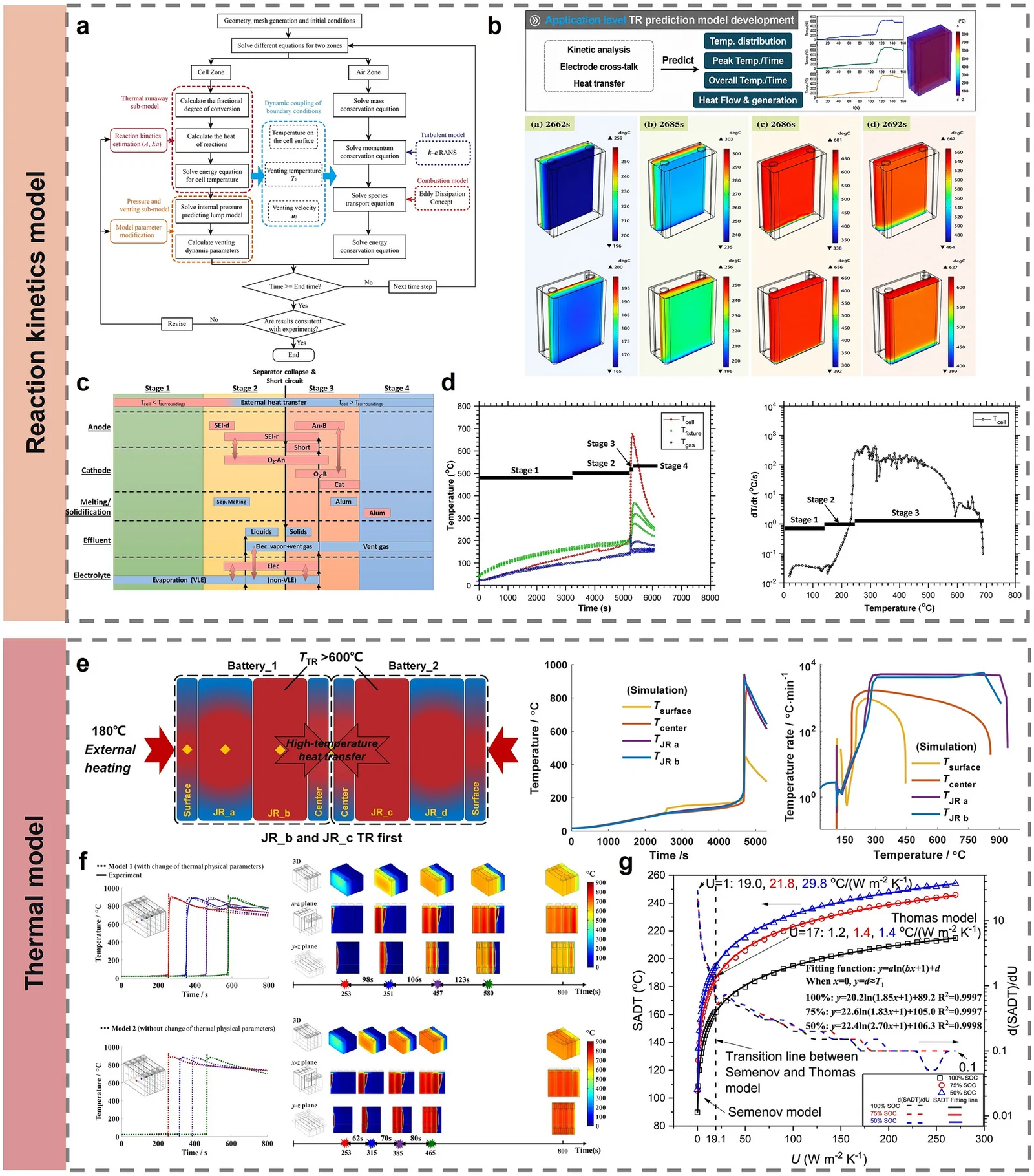
**a** Flow chart of the thermal runaway model. **b** Development, validation and investigation of the TR-prediction model are illustrated and the temperature distribution map of characteristic periods under local overheating. **c** Sequence of events for thermal abuse of MC992 18,650 cell. **d** Temperature versus time and dT/dt versus temperature for 18,650 NMC992 thermal abuse experiments. **e** TR behavior at different positions. **f** Thermal runaway propagation model with/without considering parameter change after thermal runaway. **g** Logarithmic relationship between SADT and U. Elsevier reproduced with permission from **a** Ref. [24], Elsevier; **b** Ref. [75], Elsevier; **c**, **d** Ref. [20], Elsevier; **e** Ref. [117], Elsevier; **f**, **g** Ref. [118]

Ostanek et al. [20] coupled a lumped thermal capacitance with a thermal resistance network to construct a model incorporating the complete chain reaction process, from SEI decomposition and electrolyte evaporation to cathode oxygen release and internal short circuit (Fig. 8c). This model not only accurately predicted temperature evolution and venting time (Fig. 8d) but also revealed the critical role of electrolyte evaporation rate in delaying or even suppressing thermal runaway. Sadeghi et al. [119] employed genetic algorithm-based inverse modeling to establish a multi-step decomposition kinetics model for 18,650 battery components, including SEI, anode, separator, cathode, electrolyte, and binder, accurately predicting mass loss and reaction temperatures at each stage, providing a kinetic tool for quantifying the thermal runaway chain reaction at the material scale. Kong et al. [24] systematically calibrated reaction kinetic parameters in constructing a coupled thermal runaway model. To accurately reflect the influence of different states of charge on thermal stability, a multistage linear-fitting method was employed to extract the thermal kinetic parameters of NCA batteries, assuming that each stage follows first-order reaction kinetics. Linear fitting for each stage yielded the activation energies and pre-exponential factors of individual reactions. This approach accounted for the contributions of different stages and quantified the thermodynamic parameters for each reaction stage at SOCs ranging from 25% to 75%, achieving a detailed stage-wise description of the thermal runaway chain reaction. On this basis, the study established a complete multiphysics coupling framework for thermal runaway, demonstrating a complete logical chain from geometric modeling, initial condition setting, and input of the aforementioned kinetic parameters to calculate conversion rates and reaction heats for each stage, solving the energy equation, and subsequently solving for internal pressure and venting dynamics (Fig. 8a). Zhang et al. [75], based on kinetic parameters extracted from multi-heating-rate DSC experiments, constructed a thermokinetic coupling model for LFP batteries. Using the Kissinger method combined with nonlinear optimization to obtain the reaction kinetic triplet, and coupling it with the heat transfer equation, they achieved accurate prediction of temperature evolution under adiabatic ARC conditions and localized overheating scenarios, revealing internal temperature gradients and propagation processes within the battery (Fig. 8b).

##### Thermal Models

Based on the clear characterization of reaction heat generation, thermal models describe the conduction, accumulation, and dissipation of heat within the battery, serving as a bridge connecting microscopic heat generation and macroscopic temperature rise.

Traditional thermal models for lithium-ion batteries often employ the lumped parameter method, treating the battery as having a uniform temperature. However, as battery dimensions increase toward larger capacities and formats, the non-uniformity of internal heat conduction becomes non-negligible. Zou et al. [88] introduced a distributed parameter method, dividing the battery into several regions by placing multiple thermocouples on its surface, with the measured temperature of each region representing the local temperature. This method yielded more accurate calculations of total heat generation and heat from intense reactions. Chen et al. [117] proposed a one-dimensional thermal resistance network model capable of simulating temperature distribution and characteristic temperatures of batteries under adiabatic and oven test conditions. This model explained the significant influence of test conditions, temperature measurement locations, and battery thickness on thermal runaway characteristics (Fig. 8e) and provided guidance for the rational placement of thermocouples. However, this model suffers from dimensional simplification. Macdonald et al. [21] systematically simulated the short-circuit-triggered thermal runaway process using lumped and segregated models, finding that thermal runaway propagates preferentially along the electrode longitudinal direction, then radially to the battery wall, and finally rapidly along the battery length. The segregated model significantly outperformed the lumped model in predicting thermal runaway trigger events and temperature distribution. Li et al. [120] comprehensively considered the coupled effects of solid-state heat conduction, fluid-solid heat exchange induced by gas jetting, and electrochemical heat generation in their model.

Based on a clear understanding of heat distribution, determining the critical conditions for thermal runaway initiation is crucial for safety design. Huang et al. [121] used modified Semenov and Thomas models to analyze the criticality of battery thermal runaway under uniform and non-uniform temperature distributions. They improved prediction accuracy by treating the key parameters of the overall reaction as weighted averages of the four exothermic reactions. This thermal abuse model can be used to analyze different composite materials through critical criteria and critical temperature distributions. Regarding thermal runaway critical models and experimental validation, Mao et al. [122] obtained the self-heating reaction kinetic parameters of batteries at different SOCs and calculated the safe storage temperature (SADT) based on a thermal explosion model. The study found a strong logarithmic relationship between SADT and the heat transfer coefficient (U) (Fig. 8g), providing quantitative guidance for the heat dissipation design of battery thermal management systems.

Once thermal runaway occurs in a single cell, how heat propagates within the module becomes a core issue in system safety design. He et al. [123] introduced a high-precision reduced-order thermal runaway network (TRN) model capable of rapidly simulating propagation processes within a module with a trigger time error of less than one minute. Simulations showed that thermal propagation within the module exhibits a significant accelerating trend, with the propagation interval between adjacent cells potentially shortening by 72%. Therefore, safety measures must take full effect within a very short time after the first cell undergoes thermal runaway; otherwise, it becomes difficult to prevent the chain reaction.

Physical parameters were based on a model optimization algorithm. Combining experiments and simulations, results showed that thermophysical parameters change significantly before and after thermal runaway, substantially affecting thermal runaway propagation behavior within the battery pack (Fig. 8f). Therefore, changes in thermophysical parameters should be considered in thermal runaway modeling for long-format batteries, Feng et al. [124] established a one-dimensional heat transfer model to quantitatively analyze the contribution of convective heat transfer induced by gas diffusion to the internal thermal runaway propagation speed in blade batteries, with increases of 6.00%–17.06% under laminar flow conditions and 5.46%–6.76% under turbulent flow conditions. The simplified model [125] with its core energy conservation equation couples local short-circuit heat generation for triggering thermal runaway with heat generation from the chain exothermic reactions of the active materials, achieving accurate description of the actual reaction kinetics.

After battery failure, thermophysical parameters undergo significant changes. Material loss due to venting during thermal runaway leads to changes in density, heat capacity, thermal conductivity, and other thermophysical parameters, affecting the safety modeling and design of energy storage battery systems. Feng et al. [118] proposed an automatic identification method for thermal battery pack design.

##### Electrical Models

Electrical models serve as a bridge connecting the external electrical behavior of the battery with the internal thermal runaway process, describing heat generation behavior and failure pathways under electrical abuse conditions such as internal short circuits, overcharge, and high-rate charge/discharge. Based on the level of mechanistic detail, electrical models can be classified into equivalent circuit models and electrochemical mechanism models.

Equivalent circuit models (ECMs) are widely used to describe the dynamic behavior of lithium-ion batteries due to their intuitive circuit representation and low computational complexity. Jeon et al. [22] further developed an integrated ECM to simulate the thermal runaway process under coupled electrical and mechanical abuse. Building on the traditional second-order RC circuit, this model characterizes the side reaction chain triggered by electrical abuse through a series resistance branch and simulates heat generation from internal short circuits induced by mechanical damage through a parallel RC network, achieving unified modeling of multiple abuse scenarios. When simulating coupled overcharge-mechanical damage scenarios, the model achieved 87.1% agreement with experimental data. Yan et al. [126] proposed a 2-RC equivalent circuit modeling and calibration method integrating circuit parameters, capacity degradation, and reaction kinetics based on high-power pulse testing and adiabatic thermal runaway experiments, achieving quantitative prediction of thermal runaway behavior in lithium-ion pouch cells under extreme conditions. Related research, based on accident analysis of thermal runaway triggered by overcharge in actual electric vehicles, introduced for the first time environmental changes and driving behavior randomness as heat source terms [127], making the model more representative of real-world vehicle operating conditions.

**Fig. 9:**
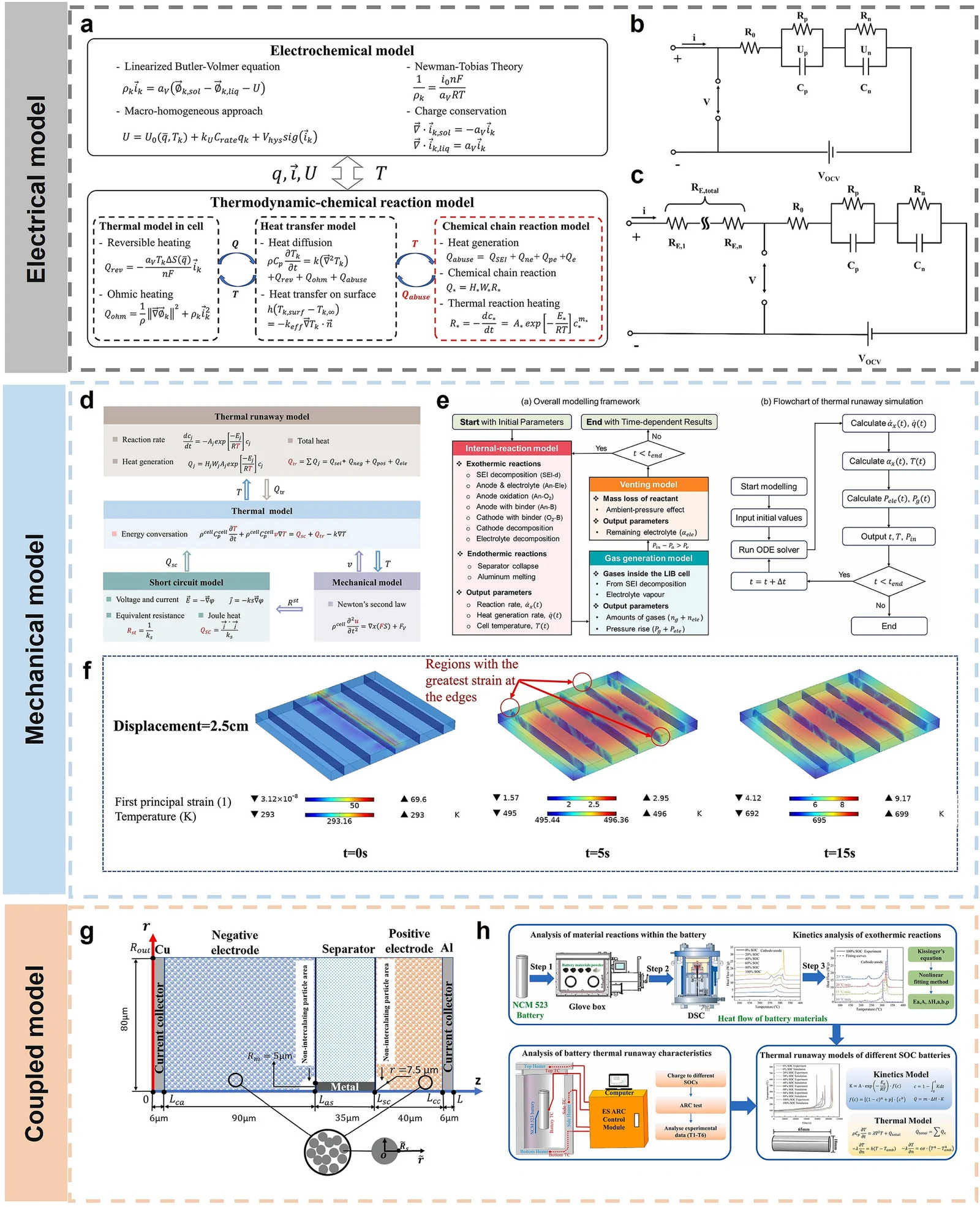
**a** An integrated framework of the proposed multiphysics model coupling electrochemical model and thermodynamic-chemical reaction model. **b** Second-order resistor-capacitor equivalent circuit model. **c** Equivalent circuit model of thermal runaway due to electrical abuse. **d** Coupling framework of mechanical, thermal, and short-circuit models for battery deformation-induced thermal runaway. **e** Overall modeling framework and the calculation process for thermal runaway simulation. **f** Temperature and tangential strain distributions in batteries under varying specified displacements. Red circles indicate high-strain regions. **g** A 2D unit cell model with metal penetrating the separator for internal short circuit modeling. **h** Modeling of TR of lithium-ion batteries under different states of charge based on reaction sequence and kinetics—an overview of this paper. Wiley reproduced with permission from **a** Ref. [129], Elsevier; **b**, **c** Ref. [22], Elsevier; **d**, **f** Ref. [11], Elsevier; **e** Ref. [130], Elsevier; **g** Ref. [131], Elsevier; **h** Ref. [132]

Electrochemical mechanism models, starting from first principles, describe lithium-ion transport in solid and liquid phases, electrochemical reaction kinetics, and heat generation mechanisms. Ertan et al. [128] proposed a high-precision thermal runaway prediction model for lithium-ion batteries based on multi-level physical field coupling. This model integrates the Newman electrochemical kinetics model, which describes charge transport and heat generation during charge/discharge, and the Hatchard decomposition model, which simulates the chain decomposition reactions of SEI, anode, cathode, and electrolyte at high temperatures. The model can predict the entire thermal runaway process of batteries under different charge/discharge rates and temperature conditions. Kim et al. [129], addressing the efficiency issue of electrochemical mechanism models, established a bidirectional dynamic coupling between an improved simplified electrochemical model and a thermodynamic-chemical reaction coupling model (Fig. 9a). By introducing temperature-dependent open-circuit voltage parameters, entropic heat parameters, and rate-dependent parameters, this model overcomes the limitation of traditional simplified models in reflecting SOC dependence. The model linearizes the Butler-Volmer equation, significantly improving computational efficiency while maintaining accuracy. For LFP and NMC batteries at 1C-3C rates, the prediction accuracy of voltage and temperature responses is comparable to that of pseudo-two-dimensional models, but the computational speed is improved by a factor of 7–8.

The resistance of the short-circuit path in actual batteries is dynamic and distributed. Studies using patterned electrodes have found [105] that under realistic abuse scenarios, the current seeks and utilizes all possible parallel, conductive pathways, making the effective short-circuit resistance much lower than simple geometric calculations. Therefore, when constructing thermal runaway prediction models, the complex internal multiphysics coupling and current distribution must be fully considered.

##### Mechanical Models

Mechanical models describe the failure pathway from mechanical abuse to internal short circuit, as well as battery swelling and rupture due to gas generation during thermal runaway. To accurately simulate the complete thermal runaway process and better understand the complex interactions among thermal, mechanical, and electrochemical processes in lithium-ion batteries, models coupling multiple physical processes, including heat and gas generation/venting, are required. Such models can be used for virtual parametric studies to quantitatively analyze the effects of factors such as electrolyte quantity and external heat dissipation conditions on thermal runaway trigger time and severity [20].

Zhang et al. [11] used COMSOL to establish a thermo-mechanical coupling model, achieving quantitative simulation of the bending-induced thermal runaway process, revealing the runaway evolution characteristics of temperature and strain distribution within the battery. Under mechanical bending, strain is concentrated on the lower surface and edge regions of the battery, and large deformation leads to highly non-uniform temperature distribution. However, this model did not consider the effects of gas release during thermal runaway (Fig. 9b). He et al. [133] developed a lumped thermal runaway model integrating gas generation and venting processes, revealing for the first time that internal pressure, rather than simply temperature rise, is the dominant factor triggering battery venting.

##### Fluid Dynamics Models

Fluid dynamics models focus on gas generation and pressure accumulation within the battery, as well as the simulation of jetting, flame propagation, and explosion risks after pressure relief, serving as a key link between internal thermal runaway and external secondary hazards. In recent years, thermal runaway modeling research has expanded from ambient pressure conditions to extreme environments. Liu et al. [130] established a lumped parameter model coupling thermal, chemical reaction, and gas generation for low-pressure environments such as aviation transportation, revealing that low-pressure environments significantly prolong the latency period from pressure relief to thermal runaway by exacerbating electrolyte evaporation loss after venting. Lowering ambient pressure can provide more emergency response time before thermal runaway occurs.

##### Coupled Models

Thermal runaway is a complex process involving deep coupling of thermal, electrical, and chemical phenomena; single-physics-field models cannot fully describe it, necessitating the development of multi-field coupled models.

To simultaneously capture the macroscopic thermal behavior and microscopic chemical evolution of thermal runaway, Yang et al. [23] developed a semi-detailed model for LIB thermal runaway chemistry. This model consists of a global part coupled with an elementary reaction mechanism part. The global part simulates the overall heat generation and temperature evolution of the battery, while the elementary reaction mechanism part includes 53 detailed chemical reactions describing processes such as SEI decomposition, cathode oxygen release, and electrolyte decomposition. Thus, the model can simulate not only temperature and heat generation evolution during thermal runaway but also dynamically describe the generation rates of various gas-phase products. It successfully reproduced the nail penetration-triggered thermal runaway propagation process.

Regarding the critical triggering conditions of thermal runaway, Zhang et al. [131] employed a decoupling-recombining modeling approach to develop a two-dimensional unit cell model for internal short-circuit modeling, systematically investigating the critical triggering conditions for thermal runaway. By calculation, they determined a safety zone map to distinguish between thermal runaway and safe regions, and further quantified the influence of key material parameters such as battery thermal conductivity and anode-electrolyte reaction activation energy on the range of the safety zone.

Although mechanistic models offer high accuracy, their computational complexity makes it difficult to meet engineering application requirements. To address this challenge, researchers have developed various coupled models that balance accuracy and efficiency. One such model, a fast and accurate multiphysics model coupling an improved simplified electrochemical model with a thermodynamic reaction model [56], achieves electrochemical-thermal response simulation from normal operating conditions to abuse conditions. By introducing SOC dependence and C-rate-related parameters, enabling efficient simulation of localized initiation and three-dimensional propagation of thermal runaway under electrical abuse conditions. Based on thermal testing and a three-dimensional thermal runaway model [132], the SOC-dependent mechanism of thermal runaway in NCM||Gr batteries was revealed (Fig. 9c). Although the core reaction sequence remains consistent across different SOCs, the reaction onset temperature significantly decreases with increasing SOC. More importantly, the cathode-anode interfacial reaction and the anode-electrolyte reaction are the two primary heat sources in the thermal runaway process.

Regarding the spatial origin of thermal runaway, three-dimensional electrochemical-thermal coupling simulations [134] revealed that hot spots consistently appear in the tab connection region. This is primarily attributed to locally elevate electronic ohmic heat generation due to current concentration. The model also quantified the dominance of cathode reaction heat and ionic ohmic heat in total heat generation at high rates, providing important insights into the preferential trigger locations for thermal runaway under electrical abuse conditions.

##### Propagation Theory Models

Starting from a more fundamental physical picture, Zhao et al. [135], drawing on solid combustion theory, abstracted the internal thermal runaway propagation within a battery as a reaction wave propagating in a uniform solid medium, establishing a one-dimensional coupled reaction-heat conduction mechanistic model. This model links complex material properties with propagation dynamics through a key dimensionless parameter β, revealing the underlying physical principle that propagation speed is governed by the ratio of exothermic intensity to activation energy.

Karmakar et al. [136] developed a hierarchical thermal runaway propagation analysis framework, constructing a cell-level heat generation model incorporating electrode chemical crosstalk mechanisms and embedding it into a module-level lumped thermal network that comprehensively considers tab conduction, air gap conduction, and convective radiation. This model revealed that in tightly packed modules, tabs serve as critical high-speed pathways for heat propagation. When a corner cell undergoes thermal runaway, heat preferentially propagates through the tabs to adjacent connected cells, determining the initial propagation direction rather than simple radial heat diffusion. Through parametric simulations, they mapped out key thermal safety spectra, clarifying the inhibitory thresholds of ambient temperature, cell spacing, and heat dissipation conditions on thermal runaway propagation (TRP), and for the first time statistically identified the finite sequence of thermal propagation paths and their probability distribution. However, the model simplified the violent physical processes accompanying thermal runaway, which may bypass conventional heat conduction paths and instantaneously induce mechanical and thermal abuse in remote cells.

To address the complexity of thermal runaway mechanisms, data-driven models use machine learning algorithms to directly learn input-output mappings from data. Kriston et al. [137] numerically simulated 780 different thermal runaway scenarios and applied machine learning methods such as principal component analysis and clustering to successfully classify thermal runaway events into five severity levels, providing a systematic simulation framework and data analysis methodology for predicting and classifying thermal runaway severity.

Various thermal runaway models have different strengths in terms of descriptive capability, computational complexity, and applicable scales. A comparative summary is provided in Table 2.

**Table 2:** Core functions, advantages and disadvantages, and applicable scales of different thermal runaway models for lithium-ion batteries

Model type	Core function	Advantages	Disadvantages	Applicable scale	Reference
Reaction kinetics model	Describes heat generation from chain reactions	Clear mechanism, reveals chemical origins	Difficult parameter acquisition, high computational cost	Material/Cell	[20, 24, 75, 119]
Thermal model	Describes heat transfer and temperature distribution	High computational efficiency, strong practicality	Many simplifying assumptions, lacks detail	Cell/Module	[88, 117, 21, 120–122]
Electrical model	Describes electrical behavior and internal short circuits	Connects external electrical behavior with internal failure	ECM has weak mechanistic basis, electrochemical models have complex parameters	Cell/Module	[22, 105]
Mechanical model	Describes structural failure and internal short circuits	Simulates mechanical abuse	Weak coupling with thermal and electrical factors	Cell	[11, 133]
Fluid dynamics model	Describes gas generation and jet flow	Simulates exhaust and combustion	High computational cost, complex boundary conditions	Cell/Module	[130]
Thermal-electrochemical-mechanical coupled model	Couples multiple physical fields	High accuracy, reveals interaction mechanisms	Complex modeling, strong parameter dependency	Cell	[23, 56, 131, 132, 134]
Propagation theory model	Provides analytical solutions	Strong physical insight, fast computation	Many simplifying assumptions, limited general applicability	Cell/Module	[135, 136]
Data-driven model	Maps relationships based on data	No mechanism required, strong adaptability	Depends on data quality and quantity	Any Scale	[137]

Although significant progress has been made in current modeling techniques, several challenges remain. The reaction kinetic parameters of models at the electrode scale heavily rely on simplified assumptions and limited experimental data, lacking high-fidelity correlation from atomic/molecular scales to macroscopic phenomena. Future efforts should integrate in situ/operando characterization techniques with multiscale calculations to refine mechanistic models. Furthermore, current multiphysics coupling models are often one-way or weakly coupled, limiting their ability to accurately predict transient battery failure behavior under complex loads. Machine learning models heavily depend on high-quality, large-scale training data, while thermal runaway experimental data are costly and risky to obtain, and existing data lack unified standards. Constructing a standardized, open-source thermal runaway database is crucial for future development.

#### Prediction Methods

Building on a deep understanding of thermal runaway mechanisms and the establishment of mathematical models, how to leverage these models to achieve accurate prediction of thermal runaway risk is the core objective of battery safety management. Thermal runaway prediction methods aim to achieve forward-looking risk perception and proactive protection, providing a decision-making basis for initiating emergency response plans [138]. Existing prediction methods are mainly divided into two categories: mechanism model-based prediction and data-driven intelligent prediction.

##### Mechanism Model-based Prediction

Mechanism model-based prediction calculates the time and temperature at which the thermal runaway critical point is reached under specific abuse conditions through simulation, representing an offline or quasi-real-time quantitative assessment method.

**Fig. 10:**
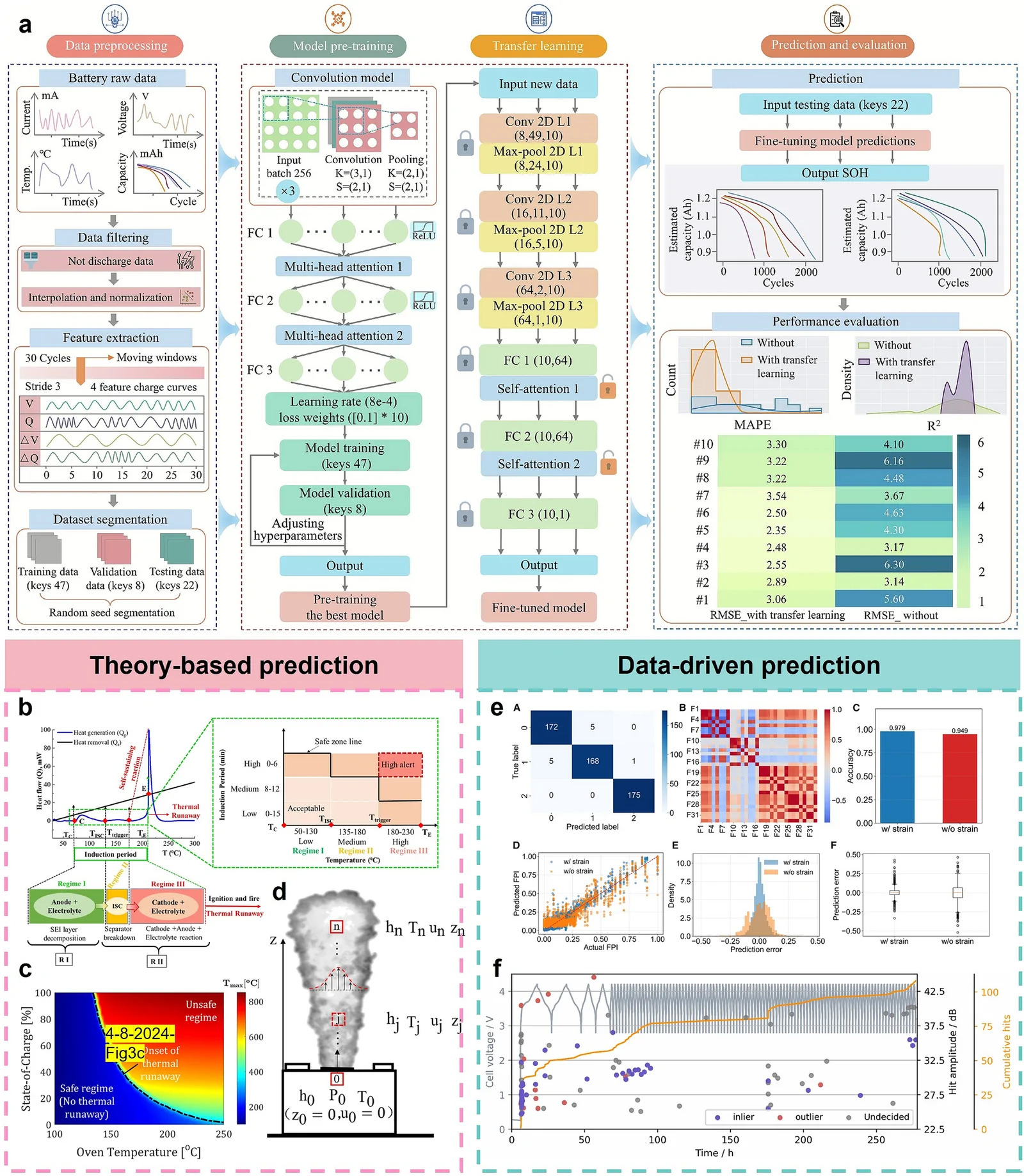
**a** Architecture of the proposed battery health diagnostic model based on multi-model fusion. **b** Evaluation of an induction period with the alert matrix from thermal behaviors of the ultrahigh capacity NCA/SCN LIB system. **c** Thermal stability map for NCA-based Li-ion cells. The black dotted line denotes the oven conditions and SOC at which TR onset is triggered. **d** Analysis model of compressible flow during the jet process. **e** Strain-assisted passive failure detection and FPI tracking utilizing machine learning. **f** AE signals measured on the Gr side during formation and cycling of an NCM-Gr full cell. Hits with ≥ 75% confidence are color-coded: blue for gas evolution (inliers) and red for particle degradation (outliers). The cumulative hit count is shown in yellow. Elsevier reproduced with permission from **a** Ref. [142], Elsevier; **b** Ref. [139], The Electrochemical Society; **c** Ref. [141], Elsevier; **d** Ref. [70], Elsevier; **e** Ref. [143], Elsevier; **f** Ref. [144]

Critical state theory provides quantitative criteria for determining whether thermal runaway will occur and when it is approaching. Padhi et al. [139], by extending Semenov theory, defined three warning temperatures: the safe boundary temperature (T_E_), the maximum temperature limit (T_M_), and the theoretical critical point (T_C_), and established a warning criterion centered on the relative relationship between T_M_ and T_E_, where T_E_ ≤ T_M_ is considered the critical state. This model was experimentally validated on ultrahigh-nickel batteries, and an alert matrix incorporating the time dimension was constructed (Fig. 10b), providing a theoretical framework for graded response. Shah et al. [140] proposed a criterion based on the thermal runaway number (TRN), which, by comprehensively considering internal thermal conduction, external heat dissipation, and heat generation characteristics, clarifies the safety boundaries for thermal management design. However, this criterion is still based on idealized assumptions and does not encompass the entire electrochemical chain reaction process. An improved Thomas thermal explosion model [138] offers an approach that balances computational efficiency and physical mechanism. By coupling the kinetic parameters of two overall reactions, cathode-electrolyte and anode-electrolyte, obtained from testing into the Thomas model, it achieved for the first time quantitative prediction of the SADT for batteries at different SOCs.

The development of analytical thermal models has further enhanced predictive capability. For cylindrical batteries, a nonlinear heat conduction equation considering multiple exothermic reactions such as SEI decomposition, electrode-solvent reaction, and electrolyte decomposition was established. Analytical solutions for the spatiotemporal temperature distribution were obtained through piecewise linearization and time-step iteration mathematical methods [68]. This approach not only accurately predicts temperature evolution but also quantitatively reveals the complex effects of thermal conductivity, cooling conditions, and reaction kinetic parameters on the thermal runaway threshold. Zhang et al. [75] developed a thermal runaway model for LFP batteries based on DSC kinetic parameters, capable of predicting temperature evolution and internal heat distribution under different thermal abuse scenarios. Karmakar et al. [141] extracted activation energies and frequency factors for the four stages of thermal runaway to construct a kinetic model capable of quantifying the effect of SOC, successfully predicting the thermal behavior of batteries in oven tests and plotting thermal stability maps (Fig. 10c), intuitively revealing the safety boundaries under the combined influence of SOC and external temperature.

ECM models support dynamic assessment of thermal runaway risk based on real-time estimation of circuit parameters. When SOC and temperature simultaneously exceed critical thresholds, the series resistance increases sharply, signaling the initiation of the side reaction chain, which can serve as an early warning signal for electrical abuse-induced thermal runaway. The current threshold set by the model can be used as a trigger criterion for mechanical abuse.

Regarding secondary hazards caused by high-temperature jet flow during thermal runaway, Zou et al. [70] proposed a method for prismatic batteries that inverts internal pressure by measuring the reaction force of the battery on the support platform, thereby predicting jet velocity (Fig. 10d). This provides a simple estimation tool for quantifying external hazards after pressure relief. However, this method assumes relatively ideal conditions and does not fully consider multiphase flow and non-steady-state effects.

##### Data-Driven Intelligent Prediction

Accurate prediction of thermal runaway faces the dual challenges of mechanistic complexity and behavioral stochasticity. Although physics-based full-order models can simulate internal reactions with high fidelity, they are complex to model and difficult to parameterize. Reduced-order models offer engineering practicality by introducing efficiency factors or reducing dimensions and can be used for battery pack-level simulations. However, neither model type can overcome the inherent high stochasticity of thermal runaway [145], which has driven prediction methods toward probabilistic risk assessment and the exploration of artificial intelligence/machine learning potential.

The integration of strain signals with machine learning provides new avenues for passive failure prediction. Gao et al. [143], combined with machine learning algorithms, achieved for the first time real-time tracking and prediction of the evolution from normal degradation to safety failure in commercial cylindrical batteries. By extracting 32 features from constant-current charging stage data and training a decision tree classifier, results showed that all passive failures were correctly predicted, with a classification accuracy of 99.7%. A feature correlation heatmap indicated that the inclusion of strain signals improved accuracy to 100%. A gated recurrent unit (GRU)-based neural network for FPI estimation (Fig. 10e) showed that models incorporating strain signals had a more concentrated prediction error distribution, with a normalized mean absolute error of only 3.82%.

Multiscale entropy and time-series analysis provide effective tools for early fault detection. Hong et al. [146] used discrete wavelet transform (DWT) to decompose electric vehicle battery data in real time, successfully detecting and locating faulty cells seven days before thermal runaway occurrence. Their team further proposed an improved multiscale entropy algorithm to achieve risk warning by calculating multilayer anomaly coefficients [147].

Acoustic emission signals and feature engineering have opened new pathways for classifying degradation processes. Noll et al. [144] generated labeled datasets through controlled degradation experiments and, combined with machine learning models, achieved for the first time classification and prediction of two key degradation processes: gas generation and particle cracking, providing a new methodological framework for BMS online diagnostics. When signals exceeded thresholds, time-domain and frequency-domain features were extracted for quantitative description. Subsequently, specific degradations were induced under controlled conditions. An isolation forest model was first trained on a gas generation dataset to learn the distribution characteristics of gas generation signals; this model was then applied to a mixed dataset, identifying data points deviating distribution as anomalies, thereby generating binary classification labels for the mixed dataset. Based on this labeled dataset, a supervised learning classifier was trained to classify signals. In long-term cycle prediction, the model predicted over 50% of events with > 75% confidence (Fig. 10f).

Deep learning architectures have demonstrated powerful capabilities in state of health estimation and fault diagnosis. Sun et al. [148] constructed a CNN-LSTM hybrid neural network for high-precision SOH prediction. The front-end CNN extracts local spatial features from multi-parameter signals such as voltage, temperature, pressure, and strain, automatically capturing patterns in the data through convolutional kernels, avoiding the subjectivity of traditional manual feature engineering. The back-end LSTM processes long-term temporal dependencies over hundreds to thousands of cycles during the aging process, effectively modeling the cumulative effects of physicochemical processes such as SEI growth and lithium dendrite deposition through gated mechanisms. The LSTM output is then mapped to SOH predictions.

Regarding recurrent neural network-based prediction models, Li et al. [149] respectively used long short-term memory (LSTM) architectures to construct models for predicting cell voltage. By comparing predicted and measured values, anomalies can be identified from minutes to days before thermal runaway occurrence. Another study by Li et al. [150] employed an improved extreme gradient boosting algorithm to predict temperature, supplemented by principal component analysis and density-based spatial clustering of applications with noise (DBSCAN) to detect temperature anomalies, achieving early warning 35 min in advance. Their team also combined convolutional neural networks with LSTM to predict abnormal heat generation, advancing the warning time to 27 min [151]. Xiao et al. [152] proposed a cloud-based, model-free unsupervised K-shape clustering method that detects thermal anomalies relying solely on the distribution characteristics of the battery data itself, providing a solution for scenarios lacking labeled data.

Generative models construct a health baseline by learning the data distribution under normal system operation, thereby detecting early anomalies that deviate from this baseline. An adversarial learning-based one-class adversarial network [153] uses an adversarial autoencoder incorporating a latent space and a visual discriminator to force the model to learn and internalize the complete characteristics of normal charging curves. During the prediction phase, the model reconstructs the input charging curve and quantifies the deviation from the health baseline by calculating the reconstruction error, effectively capturing the evolution trend of anomalies over time. While ensuring complete detection of all anomalies, it significantly reduces the false positive rate to 1.75%.

Hybrid methods integrating mechanistic knowledge with data provide system-level solutions for proactive management of thermal runaway risk. One study proposed a method integrating fault tree (FT), dynamic Bayesian network (DBN), and support vector regression (SVR). FT qualitatively describes the logical pathways of thermal runaway; DBN performs dynamic, uncertain quantitative risk assessment on this basis; and SVR further predicts risk evolution trends. This approach can provide dynamic, probabilistic risk warnings for battery systems and is a key technology for implementing predictive health management [154].

Various thermal runaway prediction methods involve trade-offs among prediction accuracy, computational speed, and prior knowledge requirements. A summary is provided in Table 3.

**Table 3:** Comparison of thermal runaway prediction methods for lithium-ion batteries in terms of accuracy, speed, prior knowledge requirements, and applicable scenarios

Prediction method	Subcategory	Accuracy	Speed	Prior knowledge requirement	Applicable scenarios	Reference
Mechanism-based models	Critical State theory	Medium-high	Medium	thermokinetic parameters	Offline design, critical evaluation	[139, 140]
	Analytical thermal model	High	Medium	reaction kinetic parameters	Temperature evolution prediction	[68, 75, 141]
	Equivalent circuit model	Medium	Fast	circuit parameters	Real-time dynamic risk assessment	[22]
	Jet Velocity inversion	Medium	Fast	mechanical parameter	External hazard quantification	[70]
Data-driven models	Signal processing	Medium	Fast	historical data	Early anomaly detection	[144, 146, 147, 152]
	Deep learning	High	Medium	training data	Voltage/temperature prediction	[148–151]
	Generative models	High	Medium	normal data	Anomaly detection, false alarm control	[153]

Thermal runaway prediction methods are evolving toward earlier, more accurate, and more reliable outcomes. Future trends are reflected in multi-source information fusion: combining multi-dimensional sensing signals such as voltage, temperature, deformation, and gas for joint prediction. Algorithmic hybrid enhancement: combining the physical interpretability of mechanistic models with the high adaptability of data-driven models. Establishing a standardized, open-source thermal runaway database to provide a foundation for model training and validation.

## Real-Time Monitoring and Early Warning Technologies for Thermal Runaway

Traditional voltage and temperature warning thresholds are typically established based on fresh batteries and may fail to identify sub-healthy states resulting from cumulative damage. Studies have shown that thermally damaged batteries may exhibit misleadingly superior initial test results compared to fresh batteries, yet their thermal runaway risk is significantly higher [25]. Therefore, developing novel sensing technologies and intelligent diagnostic algorithms capable of directly probing the internal SOH of batteries has become critical for overcoming the limitations of traditional warning approaches.

### Real-Time Monitoring Technologies for Thermal Runaway

**Fig. 11:**
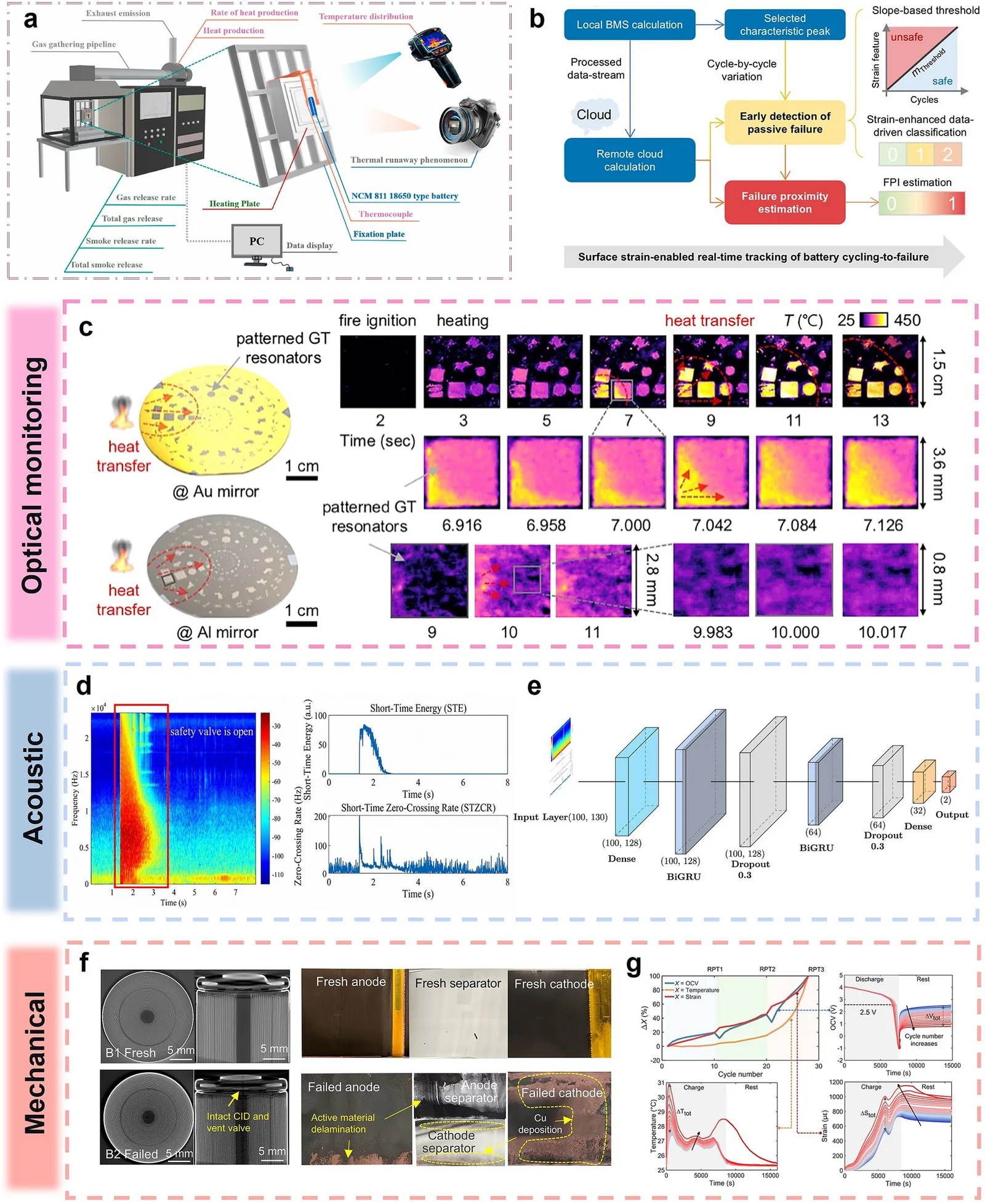
**a** Schematic diagram of TR test method for NCM811 18,650 battery. **b** Strain-enabled real-time monitoring of passive failure in lithium-ion batteries. Slope-based thresholds and failure proximity index (FPI) capture the degradation-to-failure transition, while strain-informed machine learning distinguishes failure precursors from routine degradation, enabling scalable on-board safety monitoring. **c** Heat transfer visualization across the wafer, demonstrated using pre-patterned GT resonators on Au mirror (top) and Al mirror (bottom). **d** Comparison of acoustic signals including early-stage safety valve abnormal sound, background noise, and safety valve opening sound. **e** Schematic diagram of the BiGRU-based prediction model structure. **f** Radial (left) and longitudinal (right) slices from micro-XCT imaging of a 0% SOC fresh battery and a failed case 3 battery. **g** Comparison of strain, OCV, and temperature responses during the transition from degradation to safety failure. Elsevier reproduced with permission from **a** Ref. [26], Elsevier; **b**–**f**, **g** Ref. [143], Wiley; **c** Ref. [156], Elsevier; **d**, **e** Ref. [28]

The thermal runaway process is accompanied by drastic changes in multiple physical quantities, including temperature, pressure, gas, and strain. A single signal is insufficient to comprehensively reflect the internal state of the battery. The development of integrated monitoring platforms has provided systematic tools for mechanistic research [26] (Fig. 11a). By integrating programmable thermal excitation, thermocouple arrays, infrared thermal imaging, and cone calorimetry systems, synchronous detection of the entire thermal runaway process can be achieved. This section will elaborate on the research progress of external non-invasive detection technologies and internal embedded sensors.

#### External Non-invasive Monitoring Technologies

Real-time monitoring of changes in external physical signals of the battery represents the most direct and easily engineerable technological pathway for early thermal runaway warning. External non-invasive monitoring technologies do not require changes to the existing battery structure or manufacturing processes, offering significant advantages such as low cost, strong compatibility, and ease of deployment. They have become the core foundation of current BMS sensing capabilities. These technologies primarily rely on continuous acquisition and analysis of externally measurable signals, including battery surface temperature, voltage, deformation, acoustic emission, and optical characteristics, to indirectly infer internal state evolution and identify anomalies (Fig. 11a).

Thermal imaging overcomes the limitation of single-point thermocouple measurements, enabling direct capture of flame spatial distribution, temperature field evolution, and extreme surface temperatures of the battery. Xu et al. [74] observed a four-stage evolution during thermal runaway using high-speed imaging and thermal imaging: battery swelling, smoke emission, jet flame, and flame extinction. The appearance and duration of the jet flame can serve as important optical characteristic signals for identifying the entry into the intense stage of thermal runaway. Mohamad et al. [155] systematically applied thermal imaging technology to real-time monitoring of thermal runaway processes induced by mechanical impact in lithium-ion batteries. Their work not only validated the unique advantages of thermal imaging in capturing transient thermal runaway events but also directly quantified the extreme temperatures of jet flames and ejected battery contents.

Thermochromic technology offers unique advantages for non-contact, visual monitoring of battery surface temperature fields. Kim et al. [156] developed a thermochromic Gires-Tournois (GT) resonator based on elemental tellurium (Te) for early warning of battery thermal runaway. This device adopts a simple three-layer “glass/Te/metal reflector” structure. When the temperature rises within the critical warning range, the Te layer undergoes reversible partial melting, causing significant changes in its complex refractive index in the visible light band, thereby modulating the reflection spectrum of the GT resonator. This sensor achieves sub-second response times and a spatial resolution of 35 µm, enabling real-time visualization of thermal diffusion processes on the battery surface through intuitive changes in reflected color (Fig. 11c). The device can be directly deposited onto the aluminum casing surface of the battery or fabricated as patterned patches.

Neutron imaging exhibits high sensitivity to lithium. Owejan et al. [157] quantified lithium distribution in Gr anodes using neutron radiography, finding that lost lithium primarily resides in the bulk electrode phase near the current collector. Song et al. [158] visualized the dynamic processes of dendrite growth and dead lithium formation using neutron tomography. Magnetic resonance imaging (MRI) can be used to monitor lithium-ion concentration distribution in the electrolyte and dendrite growth. Grey et al. [159] used ^7^Li NMR to visualize the correlation between dendrite growth and lithium-ion concentration gradients in lithium metal batteries. Jerschow et al. [160] used ^1^H MRI to indirectly visualize dendrite growth by measuring hydrogen atom signals in the electrolyte. Ultrasound imaging is highly sensitive to gases and voids and can be used to monitor electrolyte wetting and interface evolution. Siegel et al. [161] first applied ultrasonic transmission imaging to lithium-ion batteries, finding that ultrasonic signals are sensitive to SOC changes. Deng et al. [162] advanced ultrasound imaging technology to achieve visualization of non-uniform electrolyte wetting and interfacial activation processes.

Acoustic vibration signals provide earlier precursors of thermal runaway. Wang et al. [28] developed an acoustic detection and localization system capable of capturing abnormal sounds before the opening of the battery safety vent (Fig. 11d), achieving ultra-early warning 791 s before complete thermal runaway and 101 s before safety vent activation, with precise localization of the anomalous cell to within 0.05 m accuracy (Fig. 11e). This acoustic-based monitoring approach promotes the development of non-invasive, anti-interference early fault diagnosis and precision management systems for batteries. However, the robustness of this method in extremely complex and noisy environments remains a challenge to be addressed. Ambient noise and vibration interference primarily originate from machines such as cooling fans and battery management systems. To address this issue, Su et al. [163] applied exhaust acoustic signals to battery safety warnings and proposed a pattern recognition system for thermal runaway sounds in battery energy storage systems, enabling the removal of environmental noise and the detection of anomalies. N. Lyu et al. [164] developed an acoustic source localization and battery fault warning method by integrating acoustic sensors with a synchronous signal acquisition card. Liu et al. [165] introduced a dual-stage early warning approach that initially employs the isolated forest algorithm to assess acoustic signals during the early phase of thermal runaway for rapid fault identification, followed by a deeper analysis to uncover more critical issues. A. Kirchev et al. [166] combined acoustic ultrasound testing with deformation measurement for LIB monitoring, providing early warnings for batteries approaching an overheating state.

Surface strain signals, as a monitoring modality that can be directly attached to the battery casing surface, exhibit unique advantages in cylindrical batteries. Gao et al. [143] demonstrated through real-time monitoring of surface strain in commercial cylindrical batteries that strain signals can effectively track the evolution from normal degradation to safety failure, addressing the limitations of traditional electrical and thermal signals, which cannot distinguish this process. Passive battery failure is characterized by its concealment. The experiment employed stress-accelerated degradation through different depths of discharge. Micro-XCT scans of three groups of batteries showed intact internal structures, but post-mortem disassembly analysis revealed that the failed group suffered severe electrode damage, including anode pulverization and peeling, as well as a reddish-brown deposition layer on the cathode surface (Fig. 11f). Strain evolution can be divided into reversible and irreversible components. Reversible strain corresponds to lattice volume changes of electrode materials during lithium intercalation/deintercalation, while irreversible strain mainly originates from cumulative deformation due to SEI thickening, particle cracking, and copper dissolution/migration. Moreover, the strain signal detected the onset of failure 50% earlier in terms of cycle number compared to the temperature signal (Fig. 11g). Figure 11b illustrates a closed-loop process where strain gauges transmit signals to the local BMS for slope threshold judgment, followed by uploading to the cloud for machine learning analysis.

Multi-signal synchronous acquisition and correlation analysis provide systematic tools for understanding the entire thermal runaway process. Zou et al. [70] successfully captured the spatiotemporal evolution of jet flow temperature fields, velocity fields, and dynamic changes in internal pressure using a combination of thermocouples, high-speed imaging, and indirect pressure measurement via reaction force recorded by a high-precision electronic balance. Wei et al. [167] achieved multi-dimensional quantitative characterization of the thermal runaway ejection process through synchronous triggering of high-speed imaging, temperature, voltage, expansion force, and mass sensors. Sun et al. [148] developed a fully printable multifunctional sensor array. Using laser patterning and screen-printing processes, they integrated temperature, pressure, strain, H_2_ gas, DMC vapor, and electrolyte leakage sensors directly onto the inner side of the battery packaging aluminum-plastic film, achieving simultaneous in situ monitoring of thermo-mechanical-chemical multi-dimensional signals with minimal impact on battery energy density.

Nanofluidic sensing technologies based on ion transport present a promising new pathway for monitoring thermal runaway in LIBs. These ion transport-dependent nanofluidic sensing systems exhibit high sensitivity and stability in detecting gas concentration changes and temperature variations [168]. Wang et al. [166] achieved precise control over key properties such as ion selectivity, ion gating, ion rectification, and ion storage by designing nanoporous membrane structures with specific dimensions. This “rational ion transport management” strategy establishes a theoretical foundation for converting external chemical or physical stimuli into measurable ionic current signals. To overcome moderate disturbances that may occur in LIBs, Ma et al. [169] conducted bio-inspired multiscale interface design and developed a moisture-sensing interface based on a “rational water management” strategy. By constructing a multiscale three-phase interface that integrates macroscopic condensation mediation, micro-nano-transport pathway regulation, and molecular-level moisture resistance, achieving stable and reliable gas detection. It is an essential capability for addressing the complex chemical environment inside batteries before and after thermal runaway. For the detection of specific hazardous gases, low-dimensional material-based CO sensors demonstrate high sensitivity and selectivity potential, attributed to their large specific surface area and tunable electronic/ionic transport properties [170]. Meanwhile, ion elastomers with micro-villus structures exhibit excellent stability in moisture sensing by modulating solvated ion transport [171]. Furthermore, hierarchical tremella-like ion membranes provide a feasible technical solution for low-power, high-sensitivity ethanol sensing [172].

In summary, LIB thermal runaway involves complex reactions across solid, liquid, and gas phases, requiring comprehensive analytical approaches. In the future, with the advancement of micro-electromechanical systems technology, implantable micro-sensors can be combined with external acoustic, optical, and thermal signals to construct a comprehensive, three-dimensional sensing network, providing richer and earlier input features for artificial intelligence algorithms.

#### Internal Embedded Sensors

Research indicates that internal thermal runaway propagation is extremely rapid [125]; the internal cascading propagation process in large-capacity lithium iron phosphate batteries takes only approximately 78 s. Under localized thermal abuse scenarios, conventional lateral temperature detection exhibits significant delays, underscoring the urgency of developing internal sensing technologies. In addition to traditional voltage and temperature signals, internal pressure, gas composition, acoustic emission, and visible light/infrared image signals are emerging as cutting-edge detection modalities.

Multi-point temperature detection is a fundamental means of enhancing detection capability [173]. For large-format batteries, traditional single-point temperature sensing is inadequate. Related research deployed 12 thermocouples on the battery surface [88] and reconstructed the two-dimensional dynamic evolution of the temperature field throughout the thermal runaway process through interpolation. In the early stage of thermal runaway, the battery surface temperature distribution is relatively uniform; however, after the battery begins to vent, temperature uniformity is disrupted. When thermal runaway occurs violently, the surface temperature difference expands sharply, exhibiting distinct hot spots and temperature gradients. This indicates that multi-point temperature monitoring and changes in its distribution pattern themselves constitute powerful warning signals.

**Fig. 12:**
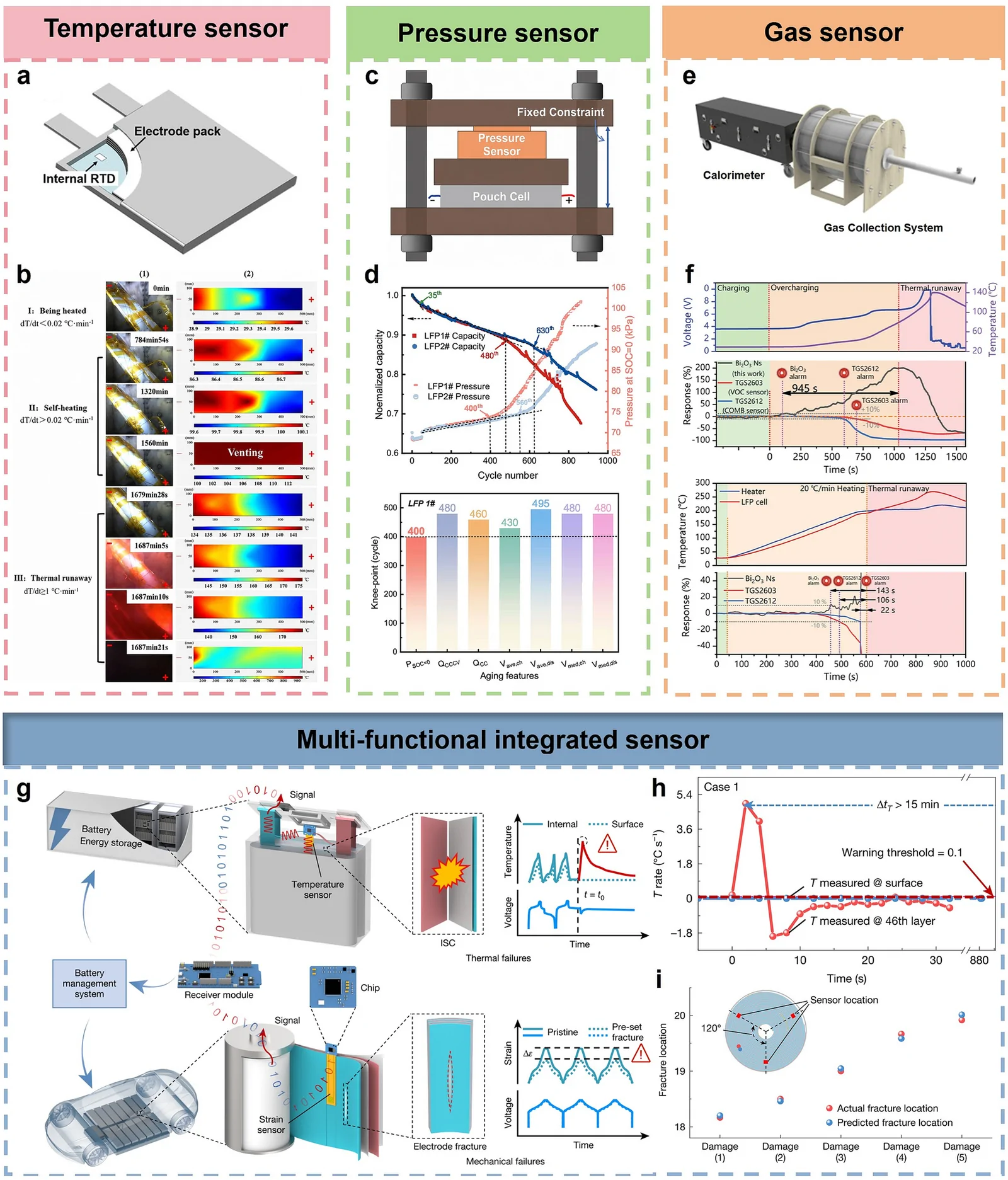
**a** Layout of RTD-embedded pouch cell. **b** Thermal runaway behavior of the cell and the surface temperature distribution obtained by interpolation at the corresponding time. **c** Illustration diagram of the set-up of the operando pressure measurement for long-term cycling experiments. **d** Comparison of the normalized capacity fade curves for LFP 1# and 2# cells with the pressure value curves at SOC = 0 per cycle and comparison of LFP 1# pressure signals PSOC = 0 with other electrical signals indicating the knee-point of nonlinear battery aging. **e** Isoview of the L-FTRC and associated gas collection system. **f** Conditions of the LFP cell and warning performances of different sensors in an overcharging test and the conditions of the LFP cell and warning performances of different sensors in an overheating test. **g** Schematic of the implantable sensing system and detection of thermal and mechanical failure modes in the LIBs. **h** Temperature ratio and schematic of electrode thermal fusing during local ISC of a prismatic battery with winding jelly roll. **i** Schematic of the implantable sensing system and detection of thermal and mechanical failure modes in the LIBs. Wiley reproduced with permission from **a** Ref. [27], Elsevier; **b** Ref. [88], Elsevier; **c**, **d** Ref. [29], The Electrochemical Society; **e** Ref. [66], Wiley; **f** Ref. [175], Springer Nature; **g**–**i** Ref. [176]

To overcome the lag inherent in surface temperature monitoring, researchers have begun exploring embedded temperature sensing technologies to directly detect internal electrode temperature changes. Li et al. [27] embedded a micro-resistance temperature detector (RTD) fabricated on a 3D-printed substrate into an NCM523||Gr pouch cell (Fig. 12a), achieving real-time monitoring of electrode temperature during overcharge. The internal RTD detected the onset temperature of SEI decomposition approximately 10 s earlier than the surface sensor, and a temperature difference of up to 200 °C was observed between the electrode and the surface during thermal runaway, demonstrating that surface temperature significantly underestimates the internal state. Internal embedded temperature sensing provides earlier and more direct thermal signals for thermal runaway. Chen et al. [174] designed a ternary composite film sensor based on carbon black (CB), polyvinylidene fluoride (PVDF), and thermoplastic polyurethane (TPU), with a thickness of only 37 μm, which can be seamlessly integrated beneath the current collector, achieving in situ, real-time monitoring of internal temperature changes in lithium-ion batteries.

Mechanical stress signals, primarily arising from internal forces generated by solid deformation and associated with volume changes of electrode materials and accumulation of side reaction products, are emerging as highly promising early warning indicators. She et al. [177] used thin-film pressure sensors to identify the moment when stress transitions from exponential growth to linear growth in large-format prismatic lithium iron phosphate batteries, using this as the starting point for thermal runaway warning and achieving warning times exceeding 20 min. Fiber Bragg grating (FBG) sensors, owing to their small size, high chemical stability, and immunity to electromagnetic interference, have become ideal tools for monitoring internal stress evolution in batteries. Albero Blanquer et al. [178] embedded FBG sensors in liquid and solid electrolyte batteries. When light passes through the optical fiber, the FBG reflects specific wavelengths; changes in temperature, hydraulic pressure, or strain around the sensor alter the effective refractive index and grating period, causing a shift in the reflected wavelength. The reflected wavelength shift is primarily attributed to strain changes, which are then converted to stress via Hooke’s law. This work achieved, for the first time, in situ, real-time monitoring of chemomechanical stress at the anode, cathode, and electrode/electrolyte interfaces.

Pressure signals, owing to their sensitivity to changes in the internal state of the battery, are emerging as highly promising early warning indicators. Qin et al. [179] placed pressure sensors in a sealed chamber to monitor real-time internal pressure evolution during thermal runaway, successfully distinguishing between electrolyte vapor pressure and gas pressure from chemical reactions. This not only achieved accurate pressure signal acquisition but also provided an experimental basis for pressure relief valve design. To identify the ejection stage during thermal runaway and assess explosion risk, Shi et al. [87] integrated thermocouples on battery tabs and pressure sensors in a sealed chamber to achieve synchronous real-time acquisition of temperature and pressure signals during thermal runaway. They observed that the tab temperature entered the thermal runaway critical state at approximately 120 °C, and the chamber pressure rapidly increased during the ejection stage. Ding et al. [29] employed precise in situ pressure detection technology (Fig. 12c) and found that during long-term cycling of constrained pouch cells, the irreversible cumulative mechanical pressure signal can indicate the inflection point of nonlinear battery degradation earlier than electrical signals. The inflection points of the irreversible cumulative pressure curves for two batteries occurred an average of 70 cycles earlier than the capacity decay curves. Further comparison with multiple electrical signals revealed that the pressure signal exhibited the most significant advantage in early warning (Fig. 12d).

Gas signals, owing to their high timeliness, are key to achieving ultra-early warning. Walker et al. [66] applied a gas collection and analysis system integrated at the end of the L-FTRC to large-scale battery thermal runaway studies (Fig. 12e). The results showed that a 134-Ah battery produced an average of 416.6 SL of gas during thermal runaway, primarily composed of CO_2_ and H_2_, along with flammable hydrocarbons such as methane and ethane. CH_4_, as a characteristic gas released in the early stages of LIB thermal runaway, is of great significance for ultra-early warning. However, CH_4_ molecules are chemically inert and difficult to adsorb and activate at room temperature. Liang et al. [180] developed a high-performance methane sensor based on a synergistic catalytic mechanism involving atomically dispersed Ru and Ag dual sites. Ru serves as the activation center, promoting C-H bond cleavage through d-orbital charge transfer, while Ag modulates the local electronic environment, lowering the CH_4_ dissociation energy barrier and stabilizing reaction intermediates. The proximity of the two sites at the atomic scale achieves a synergistic enhancement effect. When integrated into a battery, this sensor achieved thermal runaway warning 90 s earlier than temperature/voltage signals. Electrolyte solvent vapor can serve as an even earlier thermal runaway marker. Studies have found [175] that the release of DMC occurs earlier than traditional marker gases such as H_2_ and CO. A gas sensor based on Bi_2_O_3_ nanosheets exhibited high sensitivity and selectivity for DMC, achieving warnings more than 15 min in advance under abuse conditions such as overcharge and overheating (Fig. 12f). However, this sensor requires further optimization in terms of practical packaging, long-term anti-poisoning, and cross-sensitivity to multiple gases. In terms of sensing material design, Wang et al. [181] proposed a “first-principles calculation (DFT) and machine learning (ML)” material design and prediction framework for multiple characteristic gases released in the early stages of thermal runaway. First, through high-throughput DFT calculations, they constructed a dataset of gas adsorption energies for 28 noble metal-doped SnS_2_ materials. Feature attribution analysis using the SHAP method revealed that the conduction band minimum and surface topology polar area of the system are the most critical physical quantities influencing adsorption energy. An interpretable and extensible rational design process for high-performance sensing materials was established.

In the field of embedded sensing technologies, the demand for simultaneous detection of multiple physical signals is increasingly urgent. Li et al. [182] developed a dual-mode synergistic sensor based on flexible nickel aerogel and platinum thin films. When pressure is applied, the pores of the Ni aerogel shrink and contact points increase, leading to increased current; when temperature rises, the electron mobility of the Pt thin film decreases, resulting in increased resistance. The response mechanisms of the two materials are independent: pressure primarily modifies the geometric structure of the aerogel, while temperature mainly modulates the crystal structure of the thin film, enabling effective decoupling of pressure and temperature signals. This work achieved, for the first time, in situ, synergistic, and decoupled real-time monitoring of pressure and temperature inside a commercial 4695 cylindrical LIB. Recent research has realized miniaturized, wireless internal temperature and strain detection systems. Chen et al. [176] developed a thin-film sensing system integrated inside commercial lithium-ion batteries (Fig. 12g), achieving wireless signal transmission through power line communication. While maintaining battery cycling stability, they successfully achieved early warning of internal short circuits (Fig. 12h) and electrode fractures (Fig. 12i). The study showed that internal temperature signals are more sensitive than surface temperature, providing warnings minutes before thermal runaway, while voltage signals relied upon by traditional BMS showed only weak changes. Furthermore, by establishing a quantitative relationship between short-circuit area ratio and thermal runaway intensity, a new metric for quantitative assessment of thermal runaway was provided. Nevertheless, this system still faces challenges in terms of cost, spatial resolution, and model generalizability.

Different real-time thermal runaway monitoring technologies exhibit distinct characteristics in terms of signal type, sensing method, and applicable stage. A summary is provided in Table 4.

**Table 4:** Comparison of multi-dimensional signal acquisition technologies for thermal runaway monitoring

Signal type	Sensing method	Monitored parameter	Advantage	Limitation	Applicable stage	Reference
Temperature	Surface thermocouple	Surface temperature	Mature technology, easy to deploy	Response lag, cannot reflect internal state	Whole process	[74, 155]
	Embedded RTD	Electrode temperature	early warning, high accuracy	Complex implantation, may affect battery performance	Pre-thermal runaway	[27, 174]
	Thermochromic sensor	Surface temperature field	Sub-second response, high spatial resolution	Requires optical window, cannot penetrate packaging	Thermal propagation	[156]
Pressure	Cavity pressure sensor	Internal pressure	Distinguishes vapor pressure from reaction gas	Requires sealed cavity, difficult to implant	Venting stage	[87, 179]
Gas	Gas sensor	DMC, H_2_, CO, etc	Up to 15-min early warning	Cross-sensitivity, long-term stability to be verified	Pre-thermal runaway	[175, 180, 181]
Acoustic	Acoustic emission sensor	Abnormal sound	early warning, precise localization	Robustness challenges in water-spray environments	Before safety valve opening	[28]
Optical	Thermal imaging	Temperature field, flame	Non-contact, visualization	Cannot penetrate packaging, affected by occlusion	Ejection stage	[74, 155]
Strain	Thin-film strain gauge	Electrode deformation	Enables internal short circuit monitoring	Complex implantation	Mechanical abuse	[29, 143, 177, 178]

### Intelligent Diagnosis and Warning Strategies

The enhancement of intelligent diagnosis and warning system effectiveness relies not only on the sophistication of back-end algorithms but also fundamentally on the improvement of front-end signal quality and feature richness. Traditional warning models predominantly depend on macroscopic signals such as voltage and temperature, which often exhibit slow and insignificant changes during the incubation period of thermal runaway. In recent years, moving the warning threshold forward, fusing multi-source information, and introducing machine learning algorithms have become research hotspots.

#### Intelligent Diagnosis

Identifying and extracting features that characterize early faults from real-time collected multi-dimensional sensing signals is the primary step in intelligent diagnosis. Intelligent diagnostic algorithms transform raw signals into quantifiable risk indicators through threshold judgment, model comparison, data mining, and artificial intelligence methods, providing a decision-making basis for active safety responses.

At the battery pack level, inconsistency among individual cells can interfere with fault detection. Zhang et al. [184] proposed an online diagnostic method using a density-based spatial clustering algorithm (DBSCAN). By extracting two key features from battery voltage data, voltage deviation increment, which characterizes sudden performance deterioration, and cumulative deviation count, which reflects long-term accumulated degradation, and performing two-dimensional clustering of these features using DBSCAN, potential thermal runaway cells exhibiting significantly different behavior from normal cells can be automatically identified without preset fault categories, suitable for practical scenarios where voltage distribution is non-normal.

For traceability diagnosis of thermal runaway trigger causes, Chio and Park [185] systematically compared multiple machine learning models, including support vector machine (SVM), naive Bayes (NB), ensemble decision tree (DTE), and multilayer perceptron (MLP), for classifying the causes of thermal runaway triggers. The results showed that the Bayesian-optimized DTE model performed best, with an average classification accuracy of 90%. Feature importance analysis revealed that the heat loss rate was the most critical feature for distinguishing different abuse modes. However, this data-driven approach is essentially a post-event diagnosis based on the outcomes of thermal runaway, and the features used cannot be applied for early warning before thermal runaway occurs. Furthermore, model performance heavily relies on limited experimental datasets, and its generalization capability under complex operating conditions remains to be verified.

A single signal is often insufficient to fully characterize the safety state of a battery. Fusing multi-source information such as temperature, voltage, and gas can improve diagnostic accuracy and robustness. Wang et al. [186] proposed a multi-factor thermal runaway risk assessment model based on fuzzy analytic hierarchy process. An evaluation index system was constructed from four dimensions: the battery itself, released gas, jet flame and high-temperature mixture, and ejected powder, covering nine sub-factors including surface temperature, thermal runaway trigger time, gas lower explosion limit, gas toxicity, impact pressure, jet height/length, powder particle size, and toxicity, forming a hierarchical risk assessment structure.

With the rapid development of deep learning technologies, artificial intelligence-based diagnostic methods have demonstrated powerful feature extraction and nonlinear modeling capabilities in the field of battery fault warning, providing technical support for earlier and more accurate anomaly identification.

**Fig. 13:**
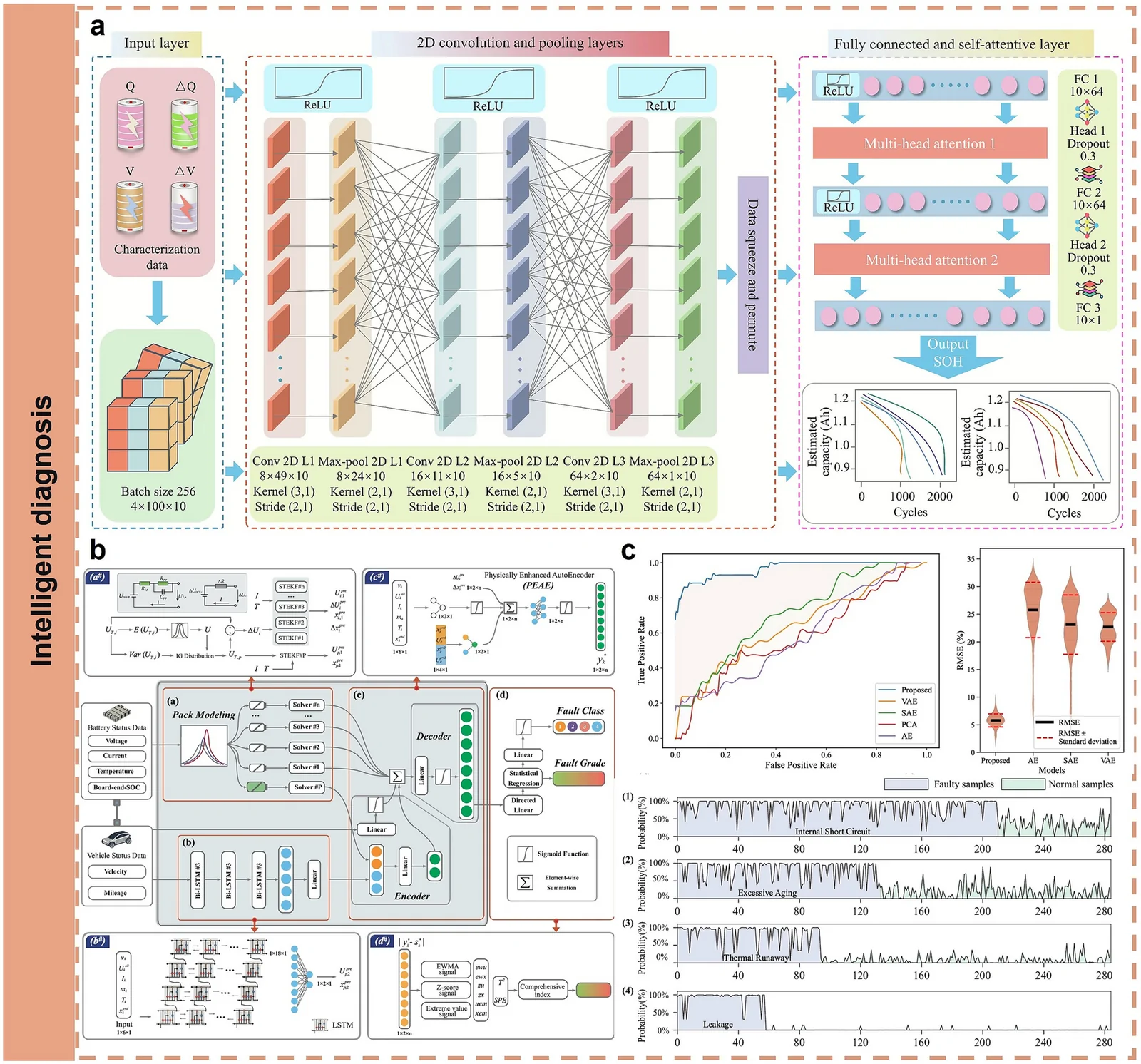
**a** Architecture of the proposed battery health diagnostic model based on multi-model fusion. **b** Detection framework based on the model-constrained deep learning network. **c** Evaluation of fault detection effectiveness and prediction accuracy. Elsevier reproduced with permission from **a** Ref. [142], Springer Nature; **b**, **c** Ref. [183]

At the level of intelligent diagnostic algorithms, Cao et al. [183] proposed a model-constrained neural network (MCNN) that effectively addresses challenges such as operational condition complexity, scarcity of fault samples, and limitations of reproducible data in battery fault diagnosis under stochastic operating conditions. This network consists of three modules: a battery pack model corrected by inverse Gaussian distribution, a temporal prediction network, and an autoencoder based on physical coupling relationships. By embedding battery physical model constraints into the deep learning framework, diagnostic performance is significantly enhanced while preserving interpretability (Fig. 13b). Validation based on 5.15 million real operating data points from 515 vehicles showed that MCNN improved the true positive rate (TPR) by 46.5%–63.7% compared to existing typical methods, with battery pack state prediction error reduced to below 9.81%. More importantly, the algorithm can simultaneously output probability distributions for four types of faults (internal short circuit, over-aging, thermal runaway, and electrolyte leakage) at the time of anomaly, with classification accuracies reaching 80.50%, 80.83%, 89.60%, and 97.76%, respectively (Fig. 13c).

In the area of model-based and data-driven fusion diagnostics, Sun et al. [187] proposed a deep neural network integrating physics-based simulation data with experimental data, achieving quantitative diagnosis of electrode-level degradation modes from electrochemical impedance spectroscopy (EIS). The model combines convolutional layers (CNN), gated recurrent units (GRU), and attention mechanisms: CNN extracts multi-frequency impedance features, GRU captures long-term dependencies during the degradation process, and the attention mechanism automatically identifies sensitive frequency ranges associated with different degradation modes. To address the scarcity of experimental degradation data, a mechanistic impedance model based on COMSOL was established and fused with early experimental data for training. This enables the model to learn a richer combination of degradation modes, significantly improving diagnostic accuracy.

Algorithms capable of online updating model parameters and adapting to individual battery differences and aging processes are key to ensuring the effectiveness of intelligent diagnosis throughout the entire life cycle. Zhao et al. [142] proposed a multi-model fusion-based battery health diagnostic architecture (Fig. 13a). Local features are extracted from input voltage-capacity curves to capture short-term spatial and temporal variations in battery performance. An attention mechanism is introduced after convolution operations to model long-range dependencies in the input sequence, capturing global degradation trends during battery aging. Through parallel processing of multiple attention heads, the model learns features from different subspaces, achieving multi-angle, multi-level data understanding, enabling millisecond-level accurate estimation of the state of health for batteries with three material systems: LFP, NCM, and NCA.

Traditional deep neural networks (DNNs) and convolutional neural networks (CNNs) primarily focus on one-dimensional feature extraction from time-series signals, lacking systematic modeling of sample or multi-sensor spatial correlations. CNNs are constrained by the translation invariance assumption and fixed convolutional kernel design, making it difficult to explicitly represent the dynamic coupling relationships of sensor nodes in non-Euclidean space. Ouyang et al. [188] proposed a LIB voltage fault diagnosis method based on an optimized graph neural network (GNN). Using the Graph SAGE model, precise localization of faulty cells within a battery pack was achieved, overcoming the limitations of traditional CNNs. Experimental results showed that the Graph SAGE model achieved a sudden fault localization accuracy of 99.90% and maintained a slow-varying fault accuracy exceeding 99%. Gaussian noise injection experiments further validated the model’s robustness, providing a new paradigm for modeling spatial correlations in battery packs using graph structures.

In recent years, unsupervised learning methods have demonstrated unique advantages in the field of battery fault diagnosis, particularly in practical scenarios where fault samples are scarce. Cong et al. [189] proposed a comprehensive signal fault diagnosis method based on variational mode decomposition (VMD), generalized dimensionless indicators (GDI), and sparse clustering. This method first uses VMD to decompose voltage signals into high-frequency dynamic components and low-frequency static components, effectively separating the interference of battery state inconsistency on fault detection. Second, a generalized dimensionless indicator construction formula is proposed, unifying features such as correlation coefficient, entropy, and waveform indicators from existing research under a common framework, from which the GDI mean deviation sequence and its forward difference are extracted as key features. Finally, a sparsity-based clustering method is employed to automatically identify outliers in the feature sequence, and a cumulative anomaly count vector with a damping factor is used to quantify the degree of anomaly. This work provides a new paradigm for threshold-free adaptive detection in voltage signal-based battery fault diagnosis.

Based on real-world electric vehicle post-crash thermal runaway accident cases, Hong et al. [190] proposed and validated an improved global Shannon entropy algorithm, combined with anomaly coefficients and anomaly rates, for battery fault diagnosis and thermal runaway tracing. The results showed that the algorithm could identify early anomalous cells 91 days before the accident and trace the propagation trend of anomalies within the battery pack, providing a data-driven diagnostic approach for early detection of short circuits during the post-crash incubation period. However, the relatively low data sampling frequency may lead to the omission of transient impact features, a concern that needs to be addressed in practical applications.

#### Warning Strategies

On the basis of early anomaly identification through intelligent diagnosis, establishing a scientific warning threshold system, and designing corresponding active safety response mechanisms constitute the final line of defense against the catastrophic consequences of thermal runaway.

**Fig. 14:**
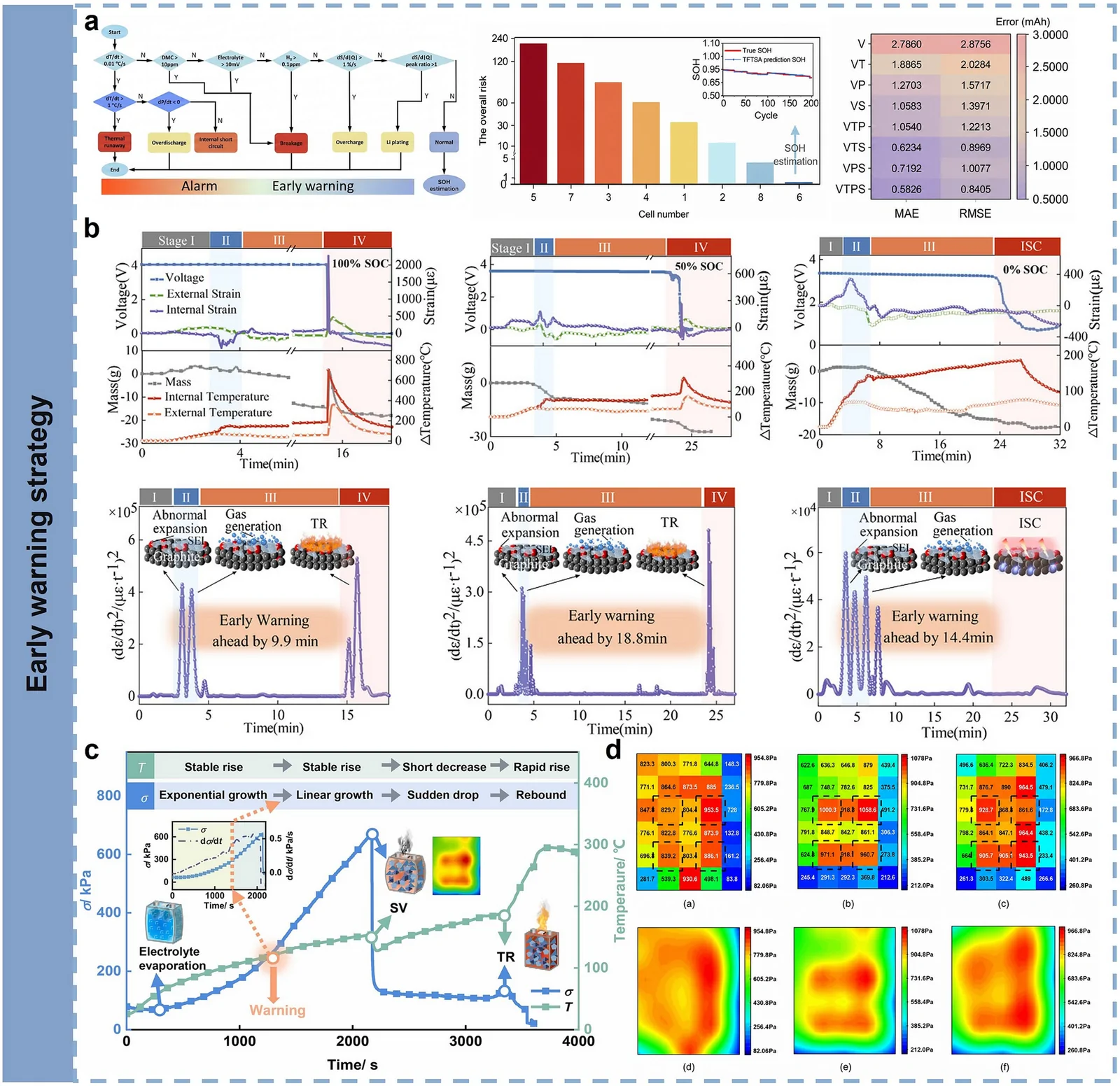
**a** Diagnosis workflow for battery-states identification, the comprehensive risk of eight sample cells and SOH estimation on the healthy cell and averaged lowest mean absolute error (MAE) and root-mean-square error (RMSE) for the validation cells with the combination of relevant features. **b** Results of multi-dimensional parameter monitoring and TR early warning during thermal abuse experiments. **c** Thermal runaway warning and mechanism based on stress signal. **d** Stress heat maps and smoothed maps of 280 Ah batteries when the safety valve opens at 100% SOC, 50% SOC, and 0% SOC. Springer Nature; **a** Ref. [148]. Wiley; **b** Ref. [195], Elsevier; **c**, **d** Ref. [177]

Establishing a graded warning threshold system and designing corresponding decision-making logic are fundamental to achieving precise response. Ji et al. [191] proposed a method for reconstructing the temperature distribution of a battery module based on a lumped parameter thermal model and three-point temperature measurement inversion, enabling real-time inversion of the temperature distribution of all cells within the module and localization of cells with abnormal heating. A three-level warning logic based on multiple parameters such as temperature and voltage was constructed, achieving a closed-loop warning process from anomaly identification to thermal runaway localization. The advantage of this method lies in balancing the limited computing power of BMS with the requirements for warning accuracy, offering a new approach for early thermal runaway diagnosis under sparse sensing conditions. Deep learning models can also be extended to early identification and classification of multiple fault types. Sun et al. [148] constructed a CNN-LSTM hybrid neural network model to achieve high-precision estimation of battery state of health. By extracting characteristic parameters such as temperature, pressure, and strain, early identification and classification warnings for six types of faults were achieved, including overcharge, overdischarge, low-temperature lithium plating, internal short circuit, mechanical puncture, and thermal runaway (Fig. 14a), providing a more comprehensive technical pathway for battery safety warning under complex operating conditions. To address the challenge of high false alarm rates under real sparse data conditions, Li et al. [153] proposed an adversarial learning-based one-class generative adversarial network model that significantly reduces the false alarm rate without compromising the detection rate, offering a feasible solution for deploying intelligent diagnostic algorithms in practical scenarios with limited data quality.

It is worth noting that the reliability of different warning indicators varies and needs to be optimized based on experimental data. Zhang et al. [192] experimentally revealed that LFP batteries exhibit a characteristic voltage drop before thermal runaway. However, further investigation showed that temperature threshold-based warnings were more reliable than those based on voltage change rate. Li et al. [193] proposed a multi-parameter collaborative warning strategy based on infrared thermal imaging, establishing three key warning indicators: temperature rise rate jump, temperature distribution convergence, and second derivative peak of voltage. When the temperature rise rate exceeded 0.03 °C s^−1^ for 10 s and the temperature distribution converged to below 3 °C, the highest-level warning was triggered, activating the liquid cooling system. The appearance of the second peak in the second derivative of voltage served as an auxiliary verification signal, further reducing the false alarm rate to 4.7%.

Addressing the thermal runaway characteristics of aviation lithium-ion batteries under low-pressure environments, Yang et al. [194] proposed a two-stage warning strategy based on voltage drop and Raman gas signals. Based on the irreversible voltage drop phenomenon before thermal runaway, the study showed that the magnitude of the voltage drop is positively correlated with SOC. To ensure reliable monitoring under all SOC conditions, the warning threshold was set at a voltage drop of 1.2 V within 3 s. Using Raman spectroscopy gas signals, the intensity of the organic solvent characteristic peak was used as the primary criterion, with a threshold set at 300 a.u. To exclude signal drift caused by environmental pressure fluctuations, the intensity difference of the N_2_ characteristic peak was introduced as an auxiliary criterion.

Compared with traditional electrical and thermal signals, mechanical signals respond more sensitively to internal gas generation reactions, enabling ultra-early warning. She et al. [177] established an innovative early warning method based on the inflection point of stress signal transition from exponential growth to linear growth. By identifying the turning point from the SEI decomposition and electrolyte evaporation stage to the stage of intensified gas generation from side reactions, thermal runaway warning more than 30 min in advance was achieved, far superior to traditional voltage and temperature signals. The stress change rate threshold corresponding to this inflection point can serve as a quantitative indicator for warning activation (Fig. 14c). Furthermore, by analyzing the stress distribution heatmap at the moment of safety vent opening, it was found that stress is concentrated in the central region of the battery with localized high-stress points, leading to the proposal of an optimization strategy for placing sensors in medium-to-high stress regions (Fig. 14d), providing guidance for engineering applications. Huang et al. [196] proposed an ultra-early thermal runaway warning technology based on battery casing strain signals. The study found that strain signals are more sensitive to thermal runaway response than temperature and expansion force signals. Three key feature points could be captured approximately 438, 214, and 143 s on average before safety vent opening, providing a valuable time window for personnel evacuation and fault handling. The study further established a correlation function with SOC, achieving quantitative graded warning of thermal runaway risk for batteries at different states of charge. Li et al. [197] introduced dimensionless analysis for the first time to confirm the feasibility of expansion force signals for warning at both cell and module levels. The study found that defining the moment when the expansion force change rate reaches 5 N S^−1^ as the onset point of abnormal expansion enabled warning lead times exceeding 15 min under overcharge and overheating conditions.

Implanting sensors inside the battery can obtain more direct and earlier warning signals. Pan et al. [195] proposed a thermal runaway warning strategy based on the internal strain derivative. By embedding FBG sensors inside the battery, in situ synchronous monitoring of internal strain and temperature under thermal, electrical, and mechanical abuse conditions was achieved. A new warning indicator based on the internal strain derivative (ISD_2_) was proposed to quantify structural dynamics intensity, such as pressure surges caused by material fracture or gas generation. Based on the ISD_2_ indicator, thermal abuse warnings were advanced by 9.9 min (100% SOC), 18.8 min (50% SOC), and 14.4 min (0% SOC) compared to traditional voltage signals (Fig. 14b).

EIS can characterize electrochemical processes within batteries at different time scales, providing rich information for early diagnosis. Dong et al. [198] developed a two-stage, three-indicator method based on EIS, utilizing temperature indicators in the mid-frequency range and deformation indicators in the high- and low-frequency ranges to issue early warnings before the battery reaches the internal short-circuit temperature. This method offers a new approach for early warning based on electrochemical characteristics.

Based on the aforementioned intelligent diagnosis and warning signals, active safety response becomes the final line of defense against catastrophic consequences. Studies have shown that triggering cooling at a threshold of 130 °C [192] can effectively interrupt the thermal runaway chain reaction in LFP batteries. Notably, even slow cooling with room-temperature nitrogen can succeed, highlighting the intervention logic that timing is more critical than intensity. Batteries that are successfully intervened can have their thermal runaway process halted and retain their primary functions, although their long-term reliability and the risk of secondary thermal runaway remain unclear.

## Thermal Protection Technologies at the Cell Level

Starting from the common mechanisms of thermal runaway, safety design at the cell material level can be achieved by increasing the T_1_ and T_2_ while reducing the T_3_ [69]. The safety design of individual cells is undergoing a profound evolution from traditional passive protection toward active-response intelligent materials. Following this logical trajectory, this chapter systematically elaborates on the latest research advances in the field of thermal safety for cathodes, anodes, electrolytes, separators, binders, current collectors, and other functional materials.

### Cathode

#### Passive Protection Cathode Materials

The thermal stability of cathode materials is a critical intrinsic factor determining the evolution path of battery thermal runaway. In the early stage of thermal runaway, chemical imbalances at the electrode-electrolyte interface typically originate from the thermodynamic degradation of the inherent crystal structure of the cathode material. Research indicates that the cathode materials with high-nickel content are more prone to irreversible layered phase transitions under thermal stress, accompanied by vigorous lattice oxygen release, thereby significantly lowering the onset temperature of thermal runaway. The release of oxygen induces extensive oxygen vacancies on the cathode surface and drives the reduction of transition-metal ions to lower valence states, subsequently enabling their dissolution into the electrolyte. These dissolved transition-metal ions, migrating through the electrolyte in solvated form, not only catalyze the exothermic decomposition of the electrolyte but may also cross the separator and diffuse to the anode side, further triggering detrimental side reactions at the anode interface. This leads to a cascade process from structural degradation of the cathode to deterioration of the anode interface. Therefore, rational modulation of the crystal structure of cathode materials constitutes an effective strategy to enhance their intrinsic thermal stability and delay thermal runaway.

The thermal runaway of cathode materials accelerates structural degradation and metal dissolution. In layered cathode materials, continuous phase transitions occur at elevated temperatures. When the cathode material exceeds the energy threshold of the oxygen 2*p* band structure, peroxide-like species form O-O bonds and release O_2_. A higher degree of delithiation lowers the activation energy for oxygen release, thereby reducing structural stability. The decomposition of the electrolyte on the material surface and the release of lattice oxygen promotes exothermic reactions, thus facilitating structural degradation and metal dissolution in the cathode [176]. Jiang et al. [177] observed that the mechanisms of thermal runaway vary with nickel content. In high-nickel LIBs, interfacial exothermic reactions dominate the heat release process. Whereas in systems with lower nickel content, O_2_ release during phase transitions and subsequent oxidation reactions are the primary causes of thermal runaway. Consequently, the thermal runaway behavior of cathode materials is closely linked to their degree of delithiation, oxygen release energy barrier, and nickel content. It is necessary to adopt differentiated thermal runaway prevention strategies for various cathode material types.

**Fig. 15:**
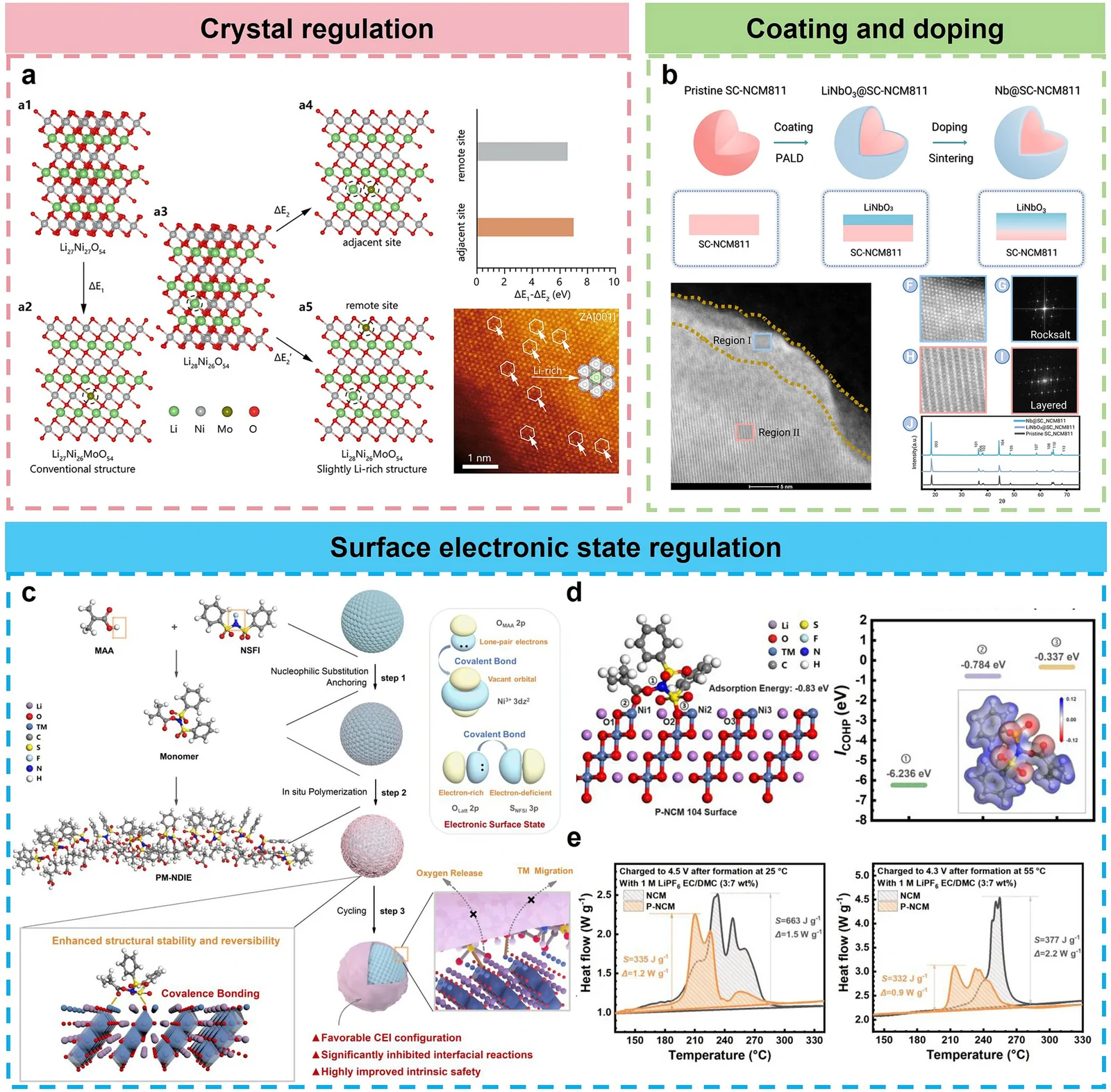
**a** Theory-experiment integrated cathode design: DFT-calculated doping energies for Mo substitution in conventional and slightly Li-rich structures, comparison of doping energy differences, and HAADF-STEM image along [001] showing Li@TM_6_ Li-rich units (white hexagons, TM = Ni, Co, Mn). **b** Modification strategy of LiNbO_3_ coating and Nb^5+ ^surface doping for the Pristine SC-NCM811. **c** Schematic diagram for electronic surface state modification of NCM cathodes using polymethacrylate-N-dibenzenesulfonimide ester (PM-NDIE). **d** Adsorption energy and calculated structure of PM-NDIE coated NCM materials. The COHP of coordination bonds and the calculated electrostatic potential of methacrylate-N-dibenzenesulfonimide ester mono­mer in the inset figure. **e** DSC curves of de-intercalated cathodes at 4.5 V/25 °C and 4.3 V/55 °C in the presence of electrolyte (1 M LiPF_6_ in EC/DMC 3:7). Wiley reproduced with permission from **a** Ref. [199], © 2025 by the authors; **b** Ref. [31], Elsevier; **c**–**e** Ref. [200]

Crystal structure regulation is fundamental to improving cathode thermal stability. Li et al. [199] proposed a strategy combining slight lithium-rich (SLR) lattice design with self-regulated molten salt synthesis to address the core challenges of lattice oxygen loss and structural collapse in nickel-rich layered cathodes under high-voltage deep charging. By introducing a small amount of Li^+^ to partially substitute Ni^3^^+^ in the transition-metal layer, Li@TM_6_ lithium-rich units were formed (Fig. 15a), reducing electrostatic repulsion toward high-valent dopants and enabling uniform distribution of doping elements, which typically segregate at the surface, throughout the particle bulk. This significantly enhanced the structural stability of the cathode material. Sun et al. [30] proposed a modification strategy to transform polycrystalline NCM811 into single-crystal NCM811. Single-crystal materials exhibit a more complete crystal structure, lower grain boundary density, and fewer internal defects. The degree of cation mixing in single crystals is significantly reduced, and internal stress and strain accumulation are weakened. Consequently, the exothermic enthalpy of their thermal decomposition reaction is substantially reduced, and the structural phase transition temperature is delayed. For 10-Ah large-format pouch cells using single-crystal NCM811, the thermal runaway onset temperature increased by 13 °C compared to cells using polycrystalline materials, and the temperature rise rate was significantly slowed.

Elemental doping and surface coating are key structural modification strategies for regulating the intrinsic thermal stability of cathode materials. Co doping not only promotes cation ordering during synthesis, reducing the degree of Li/Ni mixing, but also significantly improves the structural stability of Ni-based materials and delays the phase transition temperature associated with thermal runaway [201], thereby improving thermal safety at the source. Introducing high-valence cations such as Al^3^^+^ and Zr^4^^+^ for doping can suppress oxygen vacancy formation by strengthening Ni-O bond energy, thermodynamically enhancing the stability of the H3 phase [202] and reducing the risk of structural collapse and oxygen release at high voltages. Furthermore, coating cathode materials with materials such as Al_2_O_3_, TiO_2_, and LFP [89] can construct an effective physical barrier, kinetically blocking oxygen release and electrolyte erosion, further enhancing cathode stability at high temperatures and high voltages. Wei et al. [31] systematically enhanced the thermal stability of single-crystal NCM811 cathode materials through a synergistic modification strategy combining LiNbO_3_ coating and Nb^5^^+^ surface doping. Two distinct atomic arrangement regions, a disordered rock-salt phase structure and an ordered layered structure, were observed on the surface of Nb@SC-NCM811, indicating that Nb^5^^+^ surface doping induced surface structural reconstruction, forming a nanoscale rock-salt phase shell. This pre-constructed rock-salt phase shell enhances the structural stability of the particle surface and protects the internal layered structure from degradation (Fig. 15b).

Electronic surface state modulation is also an effective strategy for enhancing the intrinsic safety of nickel-rich layered oxide cathodes. Cheng et al. [200] used poly(methyl methacrylate-N-diphenylsulfonimide) (PM-NDIE) to modify the surface of NCM (Fig. 15c). Through in situ polymerization, the nucleophilic carbonyl oxygen formed coordination bonds with under-coordinated Ni^+^/Ni^2+^, while the electrophilic sulfonyl sulfur atoms bonded with under-coordinated lattice oxygen (Fig. 15d), thereby stabilizing the electronic states on the NCM surface and significantly inhibiting thermally induced phase transitions and oxygen release. In DSC tests, P-NCM exhibited a 49% reduction in total heat release at 4.5 V and 25 °C, with the instantaneous maximum heat flow decreasing from 2.2 to 0.9 W g^−1^ (Fig. 15e).

Electrode microstructure design has also been proven to be an effective approach for enhancing abuse tolerance. Wang et al. [203] doped electrodes with 1 wt% angular carbon black particles as a damage homogenizer (DH). When the battery was subjected to high-speed impact, the weak interface bonding between DH particles and the matrix induced widespread micro-damage within the electrode, thereby increasing the resistance of the internal short-circuit path and reducing total heat generation by approximately 40%.

In sulfide all-solid-state batteries, which are considered a high-safety route, the active oxygen released from nickel-rich cathodes upon heating undergoes vigorous solid-solid interfacial exothermic reactions with the sulfide solid electrolyte (LPSC), with heat release up to 20 times that of the cathode itself, accompanied by the release of toxic gases. Studies have shown [204] that increasing the compaction pressure of the composite cathode can significantly suppress this exothermic reaction, reducing total heat release by approximately 40%. The mechanism lies in the fact that high pressure promotes the in situ formation of a polysulfide P_2_S_x_ protective layer at the interface and reduces porosity, blocking further reactions between oxygen and the electrolyte. However, the universality of this approach under full-cell conditions, dynamic pressure conditions, and aged interfaces remains to be verified, and the potential mechanical damage from high pressure, as well as the active prevention and control of toxic gas release, are challenges that must be addressed.

Beyond the aforementioned conventional strategies, recent research has further developed targeted modification approaches specifically designed to suppress the generation and migration of active oxygen species, a primary trigger for thermal runaway at the cathode-electrolyte interface, particularly in high-voltage and high-temperature operation of high-energy-density batteries [90, 98].

One effective strategy is oxygen vacancy engineering at the cathode surface. By constructing a defective, oxygen-deficient surface layer through aliovalent doping or controlled reduction treatment, localized trapping sites are created that convert active oxygen species into less reactive lattice oxygen, thereby suppressing their attack on the electrolyte. Liang et al. proposed a “pinning strategy” to construct stable oxygen vacancies in an ultrahigh-nickel single-crystal cathode, achieving 86.16% capacity retention after 200 cycles at 4.6 V [90]. Electron paramagnetic resonance (EPR) and in situ differential electrochemical mass spectrometry (DEMS) confirm that such defective surfaces markedly reduce ^1^O_2_ evolution and overall oxygen release. Another emerging approach is lattice anchoring via high-valence cation doping (e.g., Ta^5+^, Nb^5+^, W^6+^, Mo^6+^), which strengthens the covalent bonding of lattice oxygen and elevates the energy barrier for oxygen evolution. Wei et al. demonstrated a synergistic strategy combining LiNbO_3_ coating with Nb^5+^ surface doping in single-crystal NCM811 [31]. The modified material exhibited higher thermal decomposition temperature, reduced heat release, and suppressed oxygen evolution, attributed to increased oxygen vacancy formation energy and elevated migration barrier of transition-metal cations. This approach simultaneously stabilizes the bulk lattice and passivates the cathode surface against oxygen attack.

Furthermore, electronic surface state modulation offers a chemical passivation route. Cheng et al. employed a poly(methacrylate-N-diphenylsulfonimide) (PM-NDIE) coating on Ni-rich NCM surfaces [200]. In this design, nucleophilic carbonyl oxygen coordinates with under-coordinated Ni and electrophilic sulfonyl sulfur bonds with under-coordinated lattice oxygen, thereby stabilizing the surface electronic states and significantly suppressing thermally induced phase transitions and oxygen release. Differential scanning calorimetry (DSC) tests showed a 49% reduction in total heat release at 4.5 V and 25 ℃, with the instantaneous maximum heat flow decreasing from 2.2 to 0.9 W g^−1^. In the context of all-solid-state batteries, composite cathode densification provides a physical route to mitigate active oxygen attack. Wu et al. systematically investigated the thermal runaway mechanism of NCM811/LPSC composite cathodes and found that increasing the compaction density promotes the in situ formation of an inert P_2_S_x_ protective layer at the interface, reducing total heat release by approximately 40% [204]. This finding demonstrates that, beyond chemical modification, physical densification effectively blocks the contact pathway between active oxygen species and the solid electrolyte.

Collectively, these targeted strategies share a common design principle, interrupting the electron transfer pathway between active oxygen species and the electrolyte interface. Future efforts should combine in situ/operando characterization techniques with multiscale simulations to establish direct mechanistic links between specific oxygen species and thermal failure, while translating these material-level strategies into industrially scalable processes for intrinsically safe, high-energy–density batteries.

#### Active-Response Cathode Materials

**Fig. 16:**
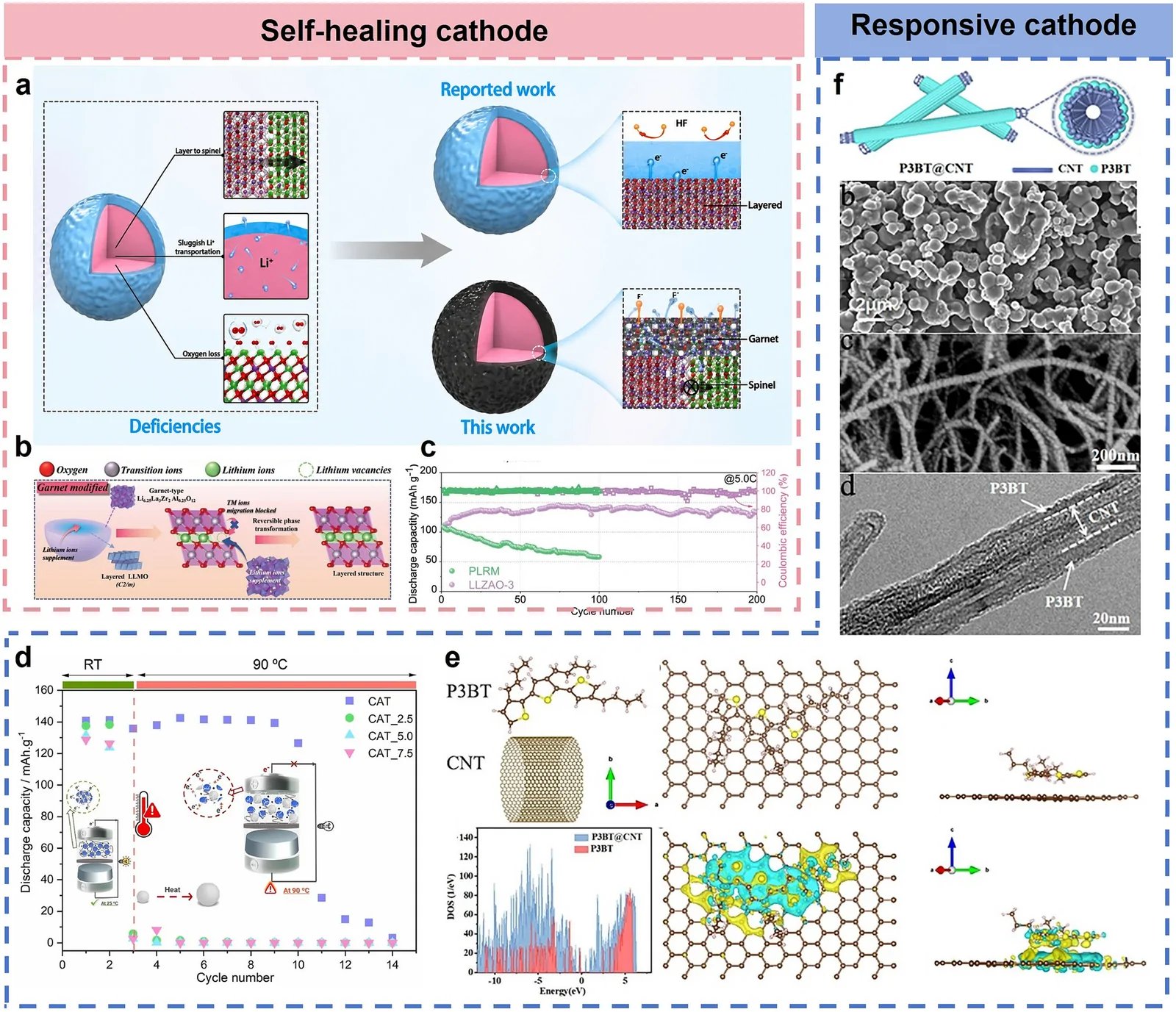
**a** Schematic of garnet modification at the level of ion migration. **b** Mechanism of phase transformation and the lithium supplement process. **c** Cycling stability of PLRM and LLZAO-3 at 5C. **d** Discharge capacities before and after applying a temperature of 90 °C and schematic illustration of the cathodes manufactured with microspheres operating principle, at 25 °C and after reaching a temperature of 90 °C. **e** Optimized configuration of P3BT and CNT, vertical and front views of the P3BT/CNT configuration, DOS calculations of P3BT and P3BT@CNT, and simulated differential charge density between P3BT and CNT. **f** Schematic diagram of P3BT@CNT, with SEM images of P3BT and P3BT@CNT, and a TEM image of P3BT@CNT. Wiley reproduced with permission from **a**–**c** Ref. [208], Elsevier; **d** Ref. [207], Elsevier; **e**, **f** Ref. [209]

Building on passive protection, researchers have begun exploring cathode materials with active-response capabilities. Temperature-responsive cathodes achieve autonomous shutdown under overheating conditions by incorporating positive temperature coefficient (PTC) materials into the cathode. Xia et al. [205] pioneered a particle-level shutdown modification strategy by directly coating PTC conductive polymers onto the surface of cathode active particles, constructing a temperature-sensitive cathode material with self-activating thermal protection. This strategy shifted the thermal protection mechanism from the electrode level down to the particle level, achieving source-level control of thermal runaway. Li et al. [206] further extended this temperature-sensitive coating strategy to nickel-rich ternary material systems. By coating NCM811 with poly(3-octylthiophene) (P3OT) via electrostatic self-assembly, a core-shell-structured NCM811@P3OT composite material was constructed. This design not only significantly improved the cycling stability of NCM811 but also endowed each active particle with overheat self-protection functionality, achieving synergistic optimization of high energy density and high safety. Monteiro et al. [207] proposed a thermal shutdown cathode based on thermally expandable microspheres. By directly mixing commercial microspheres into the electrode slurry, the irreversible volume expansion of the microspheres at critical temperatures disrupted the conductive network and ion transport pathways within the electrode (Fig. 16d), achieving thermal shutdown functionality at low cost.

Structural self-healing cathodes provide a new paradigm for suppressing capacity fade. Wei et al. [208] proposed an electrochemical lithium replenishment strategy by coating lithium-rich garnet-type solid electrolyte Li_6.25_La_3_Zr_2_Al_0.25_O_12_ (LLZAO) onto the surface of lithium-rich manganese-based layered oxide (LLMO) particles (Fig. 16a). LLZAO is inherently rich in lithium ions. During cycling, driven by concentration gradients, lithium ions from LLZAO can migrate to the LLMO surface, occupying vacancies in the lithium layer, thereby significantly increasing the energy barrier for transition-metal ion migration, suppressing irreversible phase transitions, and achieving self-healing of the lithium-rich cathode structure (Fig. 16b). The initial discharge capacity of the LLZAO-3 cathode was significantly higher than that of the uncoated LLMO, and it exhibited superior cycling stability (Fig. 16c).

Intrinsic material response represents the ultimate integrated form of intelligent response. Li et al. [209] directly endowed the cathode itself with thermal response functionality. Using p-type conductive polymers such as P3OT and poly(3-butylthiophene) (P3BT) as active materials, they constructed P3OT@CNT and P3BT@CNT nanocomposites by compositing with carbon nanotubes (CNTs). P3BT@CNT exhibited a composite structure with CNTs as the core and a P3BT nanoshell (Fig. 16e). DFT calculations revealed strong interactions between P3BT and CNTs, with charge transfer from CNTs to the thiophene rings of P3BT via π-π conjugation, which facilitated the stabilization of charge and discharge states and enhanced the cycling stability of P3BT@CNT (Fig. 16f). This resulted in an organic cathode that simultaneously achieved good electrochemical performance and overheat self-protection functionality.

### Anode

The thermal stability of the anode–electrolyte interface is primarily determined by the thermal decomposition of the SEI layer. The thermal stability of the SEI exhibits a distinct stage behavior with increasing temperature. The organic components decompose first and followed by inorganic components. Furthermore, transition-metal ions dissolved from the cathode play a critical role in catalyzing interfacial reactions at the anode. Once introduced into the electrolyte, these ions deposit within the SEI in the form of organometallic complexes [187]. These deposits act as catalysts for the decomposition of the electrolyte triggering additional side reactions, gas evolution, and compromising the structural integrity of the SEI. This degradation allows the electrolyte to continuously infiltrate and decompose at the anode surface. The sustained interfacial side reactions consume active Li^+^, leading to a “bottom-up” growth pattern of the SEI, whose progressive thickening amplifies cell polarization. Therefore, suppressing volume effects and stabilizing interfacial chemistry through surface coating, structural design, and the development of novel anode systems are important approaches for enhancing battery thermal management efficiency and safety reliability.

#### Passive Protection Anode Materials

**Fig. 17:**
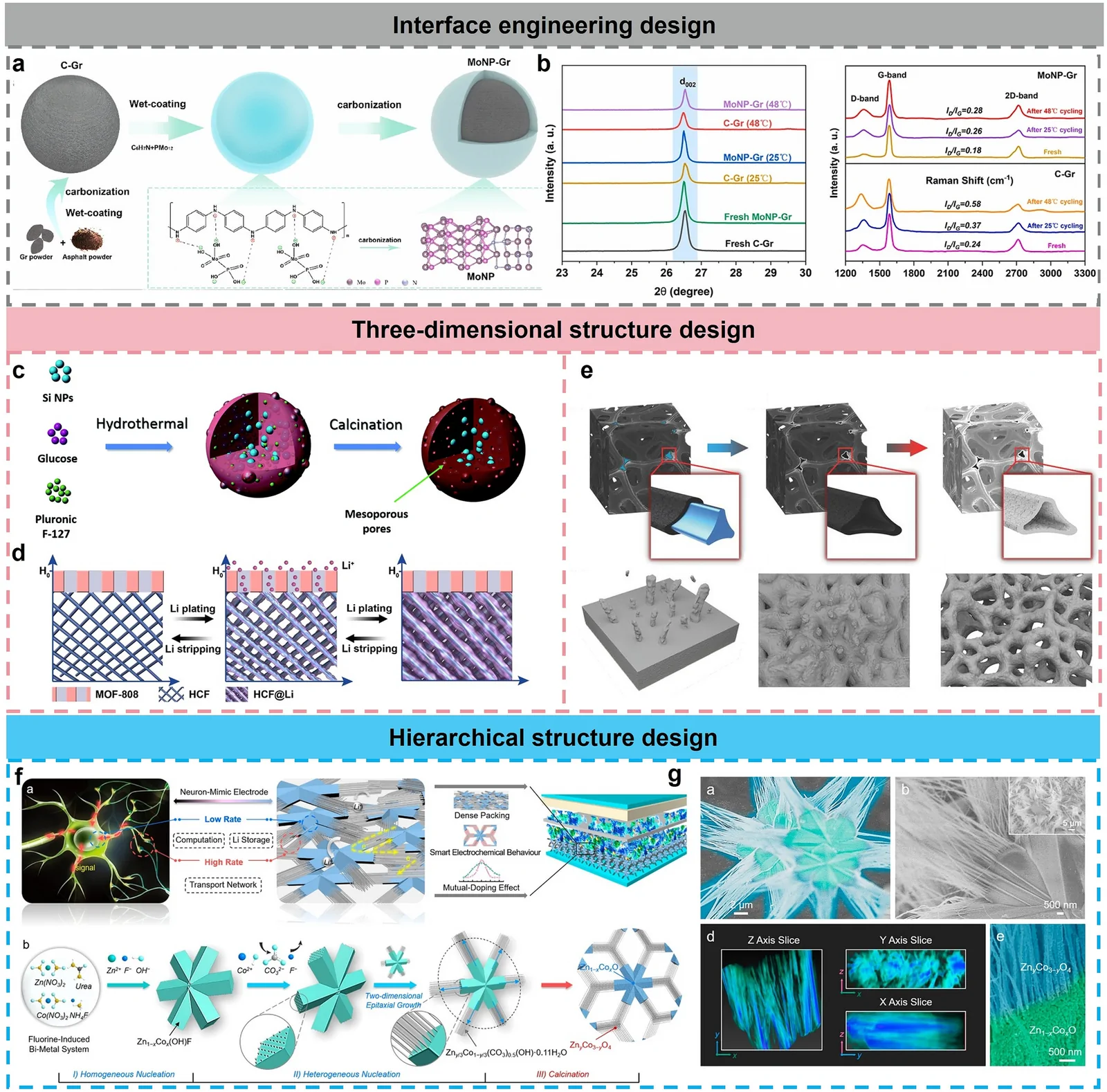
**a** Schematic illustration of the synthesis process for MoNP-Gr anode. **b** XRD spectra and Raman spectra before and after cycling. Surface and cross-sectional SEM images of C-Gr and MoNP-Gr electrodes after half cell 2000 cycles at 25 ℃ and 2C. **c** Design and fabrication of the MP-Si/C composite. **d** Schematic illustration of the Li plating/stripping process on MOF-HCF current collector. **e** Schematic diagram of the fabrication procedure of Li foam. **f** Schematic illustration of neuron-mimic smart electrode design and corresponding performance advantages and synthetic procedure. **g** SEM images of the neuron-mimic precursor at various magnifications, reconstructed 3D tomograms of the regional-nanoarray subunit, and SEM image of the Zn_1-x_O/Zn_y_Co_3-y_O_4_ heterojunction structure. Elsevier reproduced with permission from **a**, **b** Ref. [210], The Royal Society of Chemistry; **c** Ref. [213], Elsevier; **d** Ref. [212], Wiley; **e** Ref. [211], American Chemical Society; **f**, **g** Ref. [214]

In terms of traditional anode material modification, carbon coating is a commonly used strategy to improve Gr anode performance. However, conventional amorphous carbon-coated Gr faces an inherent trade-off between fast-charging capability and high-temperature stability. Amorphous carbon layers contain abundant structural defects, dangling bonds, and edge sites, which can catalyze electrolyte decomposition at high temperatures, accelerating SEI degradation and leading to gas generation and capacity fade. To address this challenge, Wei et al. [210] proposed a Mo_2_N-MoP (MoNP) nanolayer interface modification strategy, constructing a multifunctional artificial interface layer on the basis of conventional carbon-coated Gr, achieving both excellent fast-charging performance and high-temperature stability (Fig. 17a). DSC showed that the C-Gr electrode exhibited severe SEI decomposition and electrolyte oxidation, while the exothermic peak of the MoNP-Gr electrode was significantly mitigated, demonstrating that the MoNP interface layer effectively suppressed side reactions at high temperatures and improved thermal safety. Post-cycle electrode characterization further confirmed (Fig. 17b) that the MoNP-Gr anode maintained a complete interface structure and low defect density after fast-charging and high-temperature cycling, whereas C-Gr exhibited severe structural collapse and SEI failure. XPS analysis revealed that a stable SEI layer rich in LiF formed on the MoNP-Gr surface, effectively suppressing dendrite growth and parasitic reactions.

Three-dimensional structural design of lithium metal anodes is a classic strategy for suppressing dendrite growth. Hafez et al. [211] grew graphene on a nickel foam template and, after etching the nickel, obtained hollow graphene foam. By controlling the amount of molten lithium infiltrated, lithium was uniformly coated on the graphene framework surface. The low-coverage lithium foam retained the open porous structure of the foam and low interfacial impedance, facilitating the formation of a more uniform and stable SEI and suppressing dendrite precipitation (Fig. 17e). Zhang et al. [212] achieved dendrite-free lithium deposition and extremely low volume change in lithium metal anodes by coating a Zr-based metal-organic framework (MOF) layer on the surface of a three-dimensional hollow carbon fiber (HCF) framework (Fig. 17d). The Zr-based MOF coating, with its regular microporous structure, acts as an ion sieve to homogenize the lithium-ion flux. Combining the MOF coating with the three-dimensional HCF framework, which provides void space to accommodate lithium deposition, enables nearly zero-volume-expansion lithium deposition.

Bio-inspired structures for silicon anodes offer new solutions to address volume expansion. Inspired by pomegranate structure [215], Shen [213] proposed a bio-inspired silicon/carbon (Si/C) composite microsphere anode material. Silicon nanoparticles with sizes of 50–100 nm were encapsulated in a mesoporous carbon matrix. The silicon nanoparticles were uniformly dispersed in the carbon matrix like pomegranate seeds, while the mesoporous carbon acted as a conductive network and volume buffer layer (Fig. 17c). This work provided a bio-inspired approach for the structural design of high-capacity alloying anodes. Inspired by watermelon structure, Xu et al. [216] proposed a Si/C microsphere anode material with a hierarchical buffer structure. Silicon nanoparticles were uniformly dispersed in a carbon matrix with internal buffer space to form micron-sized secondary particles, and the particle size distribution was optimized to achieve close packing between particles. This dual protection strategy simultaneously addressed the volume expansion issue of silicon anodes and the electrode compaction density requirement.

Hierarchical structural design is an important research direction for high-performance anodes. Xiao et al. [217] employed a capillary contraction strategy to address the issues of electrical contact failure and structural damage caused by volume expansion during cycling in high-capacity non-carbon materials. This strategy first encapsulated multi-walled CNTs and active nanoparticles in graphene oxide hydrogels via a hydrothermal method, followed by densification of the graphene network through evaporation-induced capillary forces. During this process, CNTs were compressed and anchored on the surface of active particles, forming pre-strained nanospring structures. The dense graphene shell provided mechanical support, while the coiled CNTs acted as flexible buffer layers, accommodating the volume expansion/contraction of active particles and maintaining dynamic electrical contact, ensuring uninterrupted electron transport during cycling. Zhang et al. [218] designed and fabricated a self-standing lithium metal anode host material with hierarchical porous structure (Cu_3_P/CoP@C/CNT), effectively suppressing lithium dendrite growth through the synergistic effects of physical confinement and chemical regulation. Inspired by neuron structure, Yu et al. [214] proposed a two-dimensional multiscale synergistic bio-inspired intelligent electrode design, constructing Zn_1-x_Co_x_O/Zn_y_Co_3-y_O_4_ heterostructures (Fig. 17f). The micron-sized main body ensured high tap density, while the regional nanoarrays provided fast capacitive contribution and lithium-ion diffusion channels, and the mutual doping effect optimized the electronic structure (Fig. 17g).

#### Active-Response Anode Materials

The stress-potential coupling mechanism provides an approach for intelligent regulation of lithium metal anodes. Huang et al. [40] introduced ferrocenecarboxaldehyde (FcCHO) during the rolling process, inducing a mechanochemical reaction with lithium metal to construct a stress-responsive metal-organic framework-derived nanoscale interface layer (Li@FcCHO). This interface layer dynamically modulates surface potential under local stress. The mechanism is based on competitive coordination between TFSI⁻ anions and Fe^2+^ versus Li^+^; under stress, TFSI^−^ shifts from Li^+^ coordination to more stable Fe^2+^ coordination, inducing interfacial potential reconstruction. Consequently, the stress generated by local protrusions during lithium deposition is converted into potential changes, suppressing further deposition at those active sites and achieving uniform dendrite-free deposition.

**Fig. 18:**
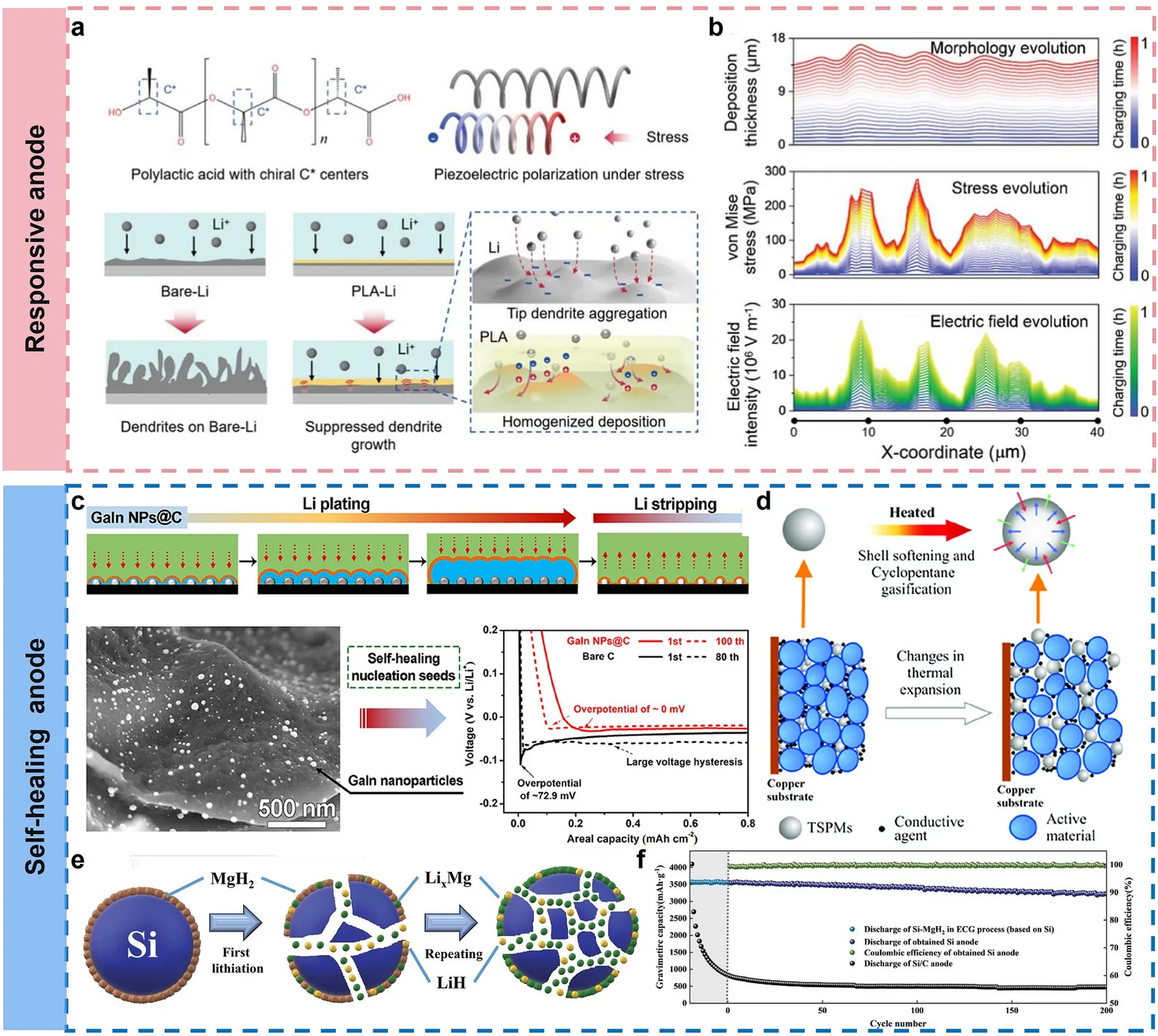
**a** Schematic diagram of the role of electromechanical coupling effect and mechanism diagram of Li^+^ regulation by built-in electric field. **b** Morphology, von Mises stress, and the corresponding electric field evolution during the charging process. **c** Uniform Li plating/stripping behavior seeded by liquid GaIn NPs: schematic illustration, SEM image of GaIn NPs@C, and voltage-areal capacity curves comparing the nucleation process on bare C and GaIn NPs@C electrodes. **d** Thermally sensitive mechanism of the TSPMs in the Gr coating layer. **e** Schematic illustration of ECG process in the Si-MgH_2_ composite. **f** Long-cycling performance of 1st, 50th, 100th, and 20th cycles of the MgH_2_-grinded Si anode at 0.5 A g^−1^. Wiley reproduced with permission from **a**, **b** Ref. [41], American Chemical Society; **c** Ref. [43]. Wiley; **d** Ref. [219], Wiley; **e**, **f** Ref. [220]

Piezoelectric adaptive regulation leverages the piezoelectric effect to achieve active control of deposition behavior. An adaptive regulation strategy based on the piezoelectric effect [41] involves coating a polylactic acid (PLA) piezoelectric polymer layer on the surface of a lithium metal anode, converting the inevitable stress changes during lithium deposition/stripping into a self-adaptive built-in electric field, actively regulating ion migration and deposition behavior (Fig. 18a). In conventional Li||LFP pouch cells, stress fluctuations during cycling are significant and unevenly distributed. The PLA piezoelectric layer converts this stress into a built-in electric field, and the electric field strength adaptively changes with the amount of lithium deposition: the more deposition and the greater the stress, the stronger the generated electric field, thereby accelerating lithium-ion migration, homogenizing local current density, and suppressing dendrite growth. Finite element simulations confirmed that this approach eliminates local current density inhomogeneity and stress concentration (Fig. 18b).

The pressure-adaptive composite lithium anode strategy [221] involves filling a three-dimensional conductive framework with the elastic polymer polyacrylonitrile (PAN). The adaptive pressure generated during lithium deposition regulates lithium deposition/stripping behavior. When lithium deposits on the conductive framework, the compressive force causes elastic deformation of the polymer, which in turn exerts a counter-pressure on the growing lithium, suppressing dendrite growth and forming a dense, smooth lithium deposition layer. Finite element simulations indicated that when the lithium deposition capacity is 1.0 mAh cm^−2^, the pressure exerted by the polymer on lithium can reach 500 MPa. However, the long-term stability of the polymer has not been fully verified.

Self-healing anodes provide a new solution for addressing mechanical damage. Zhai et al. [43] proposed a self-healing nucleation seed strategy using liquid Galn alloy nanoparticles. Galn liquid metal nanoparticles with room-temperature liquid characteristics were uniformly loaded on a carbon layer surface. During lithium deposition, these nanoparticles first undergo alloying reactions with lithium to form Ga-In-Li alloys, reducing the lithium nucleation barrier. After complete lithium stripping, the GaIn nanoparticles return to the liquid binary eutectic phase, achieving self-healing of structural damage and maintaining lithiophilic nucleation functionality throughout long-term cycling. Notably, the GaIn NPs@C electrode maintained near-zero nucleation overpotential after 100 cycles (Fig. 18c). Zhang et al. [222] proposed a synergistic strategy combining liquid metal (LM)-assisted self-healing with detachment acceleration for modifying black phosphorus/Gr (BP/G) composite anodes. LM buffers the volume expansion stress of BP during lithiation and returns to the liquid state after delithiation, repairing microcracks in BP particles and reconnecting conductive pathways.

Self-healing artificial SEI provides a new approach for stabilizing silicon anode interfaces. Patnaik et al. [41] designed a linear polyborosiloxane (PBS) as an artificial SEI layer for thin-film silicon anodes. Leveraging its self-healing properties, anion capture effects, and the Lewis acidity of tricoordinate boron, PBS effectively mitigates volume expansion and interfacial impedance growth in silicon anodes. PBS interacts with hydroxyl groups on the Si/SiO_2_ electrode surface via B-O coordination bonds, forming a strong interfacial bond. SEM observations showed that after mechanical scratching, the PBS coating completely self-healed within 30 min at 45 °C, attributed to polymer chain diffusion and reconstruction of dynamic B-O coordination bonds. A self-healing artificial SEI design based on a TiO_2_ shell [223] involves encapsulating silicon nanoparticles in a TiO_2_ shell. During the initial lithiation, the expanding silicon squeezes the electrolyte out of the shell, while a natural SEI forms on both the silicon surface and the inner TiO_2_ wall, sealing cracks. Upon delithiation, the silicon contracts, leaving a self-healed SEI composite shell. Even if the TiO_2_ shell partially ruptures after compression, this structure can still restore its protective function through the self-healing mechanism.

Temperature-responsive anodes represent an effective strategy for achieving thermal shutdown. A self-triggering strategy for lithium-ion batteries based on thermally responsive polymer microspheres [224] involves coating polyethylene (PE) or paraffin microspheres on the anode surface, achieving irreversible thermal shutdown under overheating conditions. When the internal battery temperature reaches the melting point of the microspheres, they melt and wet the electrode surface, forming a dense non-conductive barrier layer that blocks lithium-ion transport, resulting in permanent battery shutdown. Deng et al. [219] proposed a positive temperature coefficient (PTC) Gr anode based on thermally sensitive polymer microspheres (TSPMs). By incorporating core-shell-structured TSPMs into the anode coating, when the battery temperature rises to the initial foaming temperature of the microspheres, TSPMs expand, causing the Gr anode to swell and the conductive network to disconnect. The electrode resistance increases sharply, cutting off the current path and preventing further thermal runaway development (Fig. 18d).

The adaptive electrochemical grinding strategy [220] cleverly transforms the inevitable pulverization issue of silicon anodes during cycling into a controllable size optimization process (Fig. 18e). Utilizing the stepwise lithiation characteristics of MgH_2_ and Si at different potentials, the internal stress generated by the conversion reaction of MgH_2_ promotes the migration of its lithiation products into the cracks of the pulverized Si particles. Over subsequent cycles, these products gradually transform into a mixed conductive network composed of ion-conductive LiH and electron-conductive Li_x_Mg. As electrochemical grinding (ECG) cycling proceeds, micron-sized Si particles are adaptively ground to an equilibrium size and encapsulated by the in situ generated conductive matrix, effectively maintaining the electrochemical activity of the fractured Si particles. After 200 cycles, the Si anode treated with ECG maintained a reversible capacity of 2118 mAh g^−1^, with a capacity retention of 91% (Fig. 18f). However, the irreversible consumption of MgH_2_ ultimately becomes an electrochemically inert component, reducing the overall gravimetric energy density of the electrode. Wang et al. [225] grafted a self-healing polyurethane-thiourea polymer onto the sulfide electrolyte Li_7_P_3_S_11_ (LPS) via a chemical grafting strategy, endowing the electrolyte with a dual dynamic bond cross-linked network. During cycling of silicon anodes, when microcracks form in the electrolyte film due to electrode volume expansion, the reversible exchange reactions of disulfide bonds and the reorganization of hydrogen bonds spontaneously repair the damage, maintaining interface integrity. This effectively addresses the interface failure issues caused by the enormous volume expansion of silicon anodes.

Additionally, Xu et al. [226] proposed an externally driven thermal safety strategy. By implementing localized cooling at the anode bottom, the anode-electrolyte reaction was successfully suppressed, thereby blocking the core exothermic step of the chain reaction.

### Electrolyte

#### Passive Protection Electrolyte

Transition-metal ions dissolved from the cathode traverse the electrolyte to reach the anode interface, thereby endowing the electrolyte with a dual role as both a transport medium and a reaction medium during the initial stage of thermal runaway. These metal ions do not exist as free species after entering the electrolyte, they coordinate with solvent molecules and anions to form solvated ions. This process alters the interfacial reaction characteristics of the electrolyte. The presence of solvated transition-metal ions increases the reduction potential of the electrolyte, enabling reduction at a lower overpotential. Concurrently, these ions function as Lewis acid catalysts, facilitating a two-electron reduction reaction of the electrolyte and substantially accelerating its decomposition. This results in an increase in gas generation, accompanied by additional heat release from side reactions [204]. However, as the primary fuel and heat source for thermal runaway, the intrinsic thermal stability of the electrolyte is fundamental to safety. Quantum chemical calculations have revealed the microscopic determinants of this stability from first principles: high dielectric constant of the solvent contributes to thermal stability; stable solvation structures formed through intermolecular interactions effectively resist thermal decomposition. However, as the core charge carrier, lithium ions lower the decomposition energy barrier of electrolyte molecules at high temperatures, exhibiting a catalytic effect [96].

**Fig. 19:**
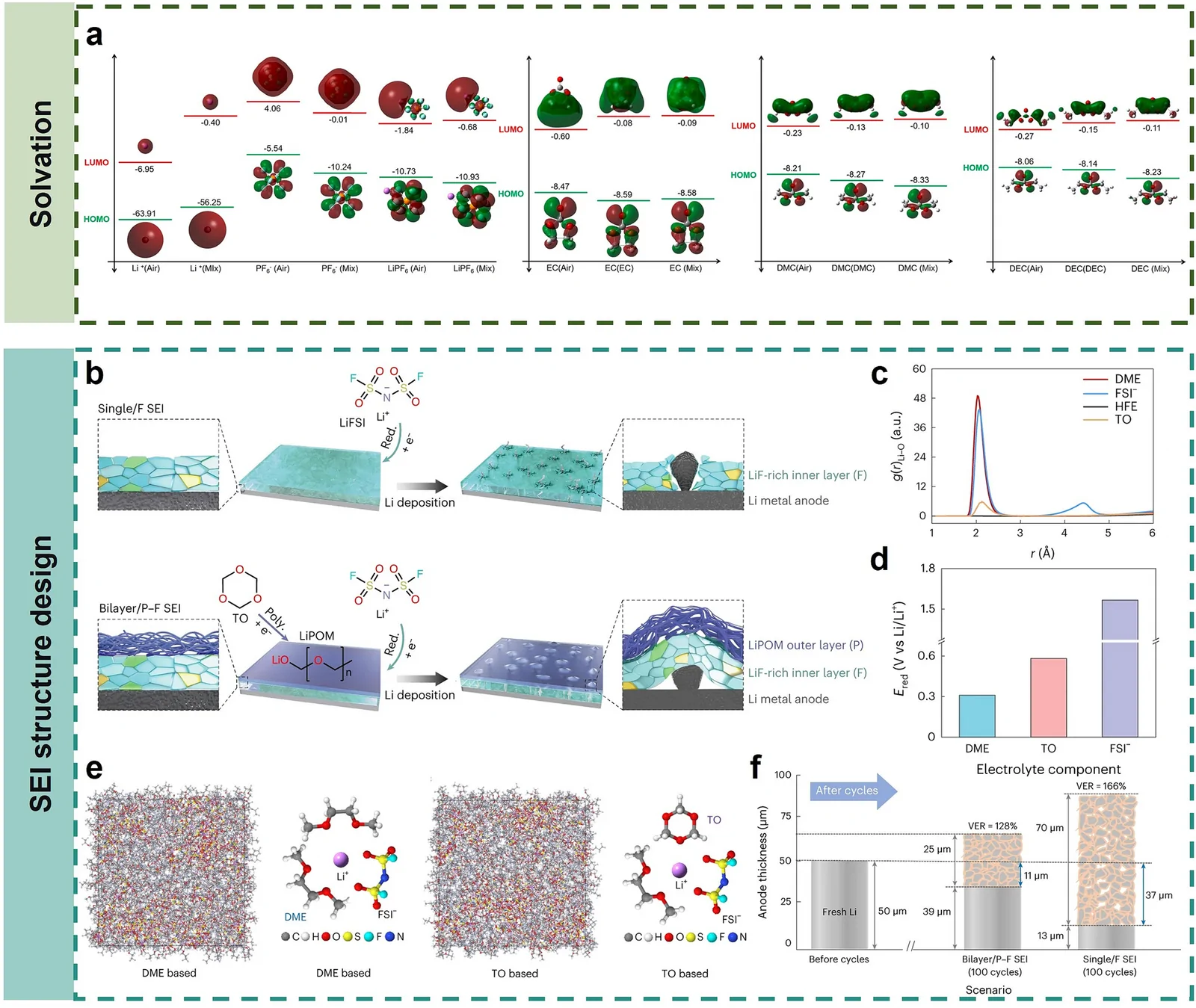
**a** HOMO and LUMO energy levels of key electrolyte constituents (LiPF_6_, EC, DMC, DEC) under different conditions. **b** Schematic diagram of the structural evolution of single-layer and tailored bilayer SEIs during Li plating. **c** RDF plots of Li-OTO pairs in TO-based electrolyte and DFT-calculated reduction potentials (E_red_) of DME, TO, **d** FSI⁻. The y-axis is broken for comparison between DME and TO. **e** MD simulation snapshots and schematic illustrations of solvation structures in DME- and TO-based electrolytes. **f** Schematic illustration of anode evolution during cycling and the corresponding volume expansion after 100 cycles. Reproduced under the Creative Commons CC BY license, © 2024 by the authors; **a** Ref. [96], Springer Nature; **b**–**f** Ref. [33]

Based on the understanding of the catalytic mechanism of LiPF_6_ decomposition products, fluorinated electrolytes have been shown to effectively suppress the catalytic effect of PF_5_/POF_3_ [32], enhancing the oxidation resistance and thermal stability of the electrolyte. However, studies have found that although fluorinated electrolytes [227] effectively delay external ignition and reduce combustion intensity, they fail to prevent or slow down the occurrence of internal thermal runaway. This is because in fluorinated systems, the reductive exothermic reactions between lithiated Gr and fluorinated components also accumulate critical energy during the self-heating stage, triggering subsequent violent exothermic chains. In response to the catalytic effect of Li^+^, regulating the solvation structure (Fig. 19a) or using more stable lithium salts to replace LiPF_6_ can fundamentally avoid the generation of PF_5_/POF_3_ and mitigate the negative catalytic effect of Li^+^.

Flame-retardant additives such as phosphates and fluorinated solvents can enhance electrolyte safety, but a trade-off with electrochemical performance must be considered. Zhang et al. [33] proposed an in situ SEI structure design strategy based on a trioxolane (TO)-modulated electrolyte. By constructing a bilayer SEI with a LiF-enriched inner layer and a lithium polyformaldehyde (LiPOM) outer layer, both the homogeneity and mechanical stability of the SEI were enhanced. LiFSI preferentially reduces to form the LiF-enriched inner layer, while TO subsequently reduces and polymerizes to form the LiPOM outer layer. The introduction of TO enables its participation in the Li^+^ solvation sheath (Fig. 19c), and its reduction potential is higher than that of DME but lower than that of FSI⁻, achieving a stepwise reduction mechanism where FSI⁻ reduces first, followed by TO (Fig. 19d). Post-cycle SEM showed that the bilayer SEI resulted in denser lithium deposition with larger grains, a 64% reduction in dead lithium, and a decrease in volume expansion rate from 166% to 128% (Fig. 19e, f).

#### Active-Response Electrolyte Design

**Fig. 20:**
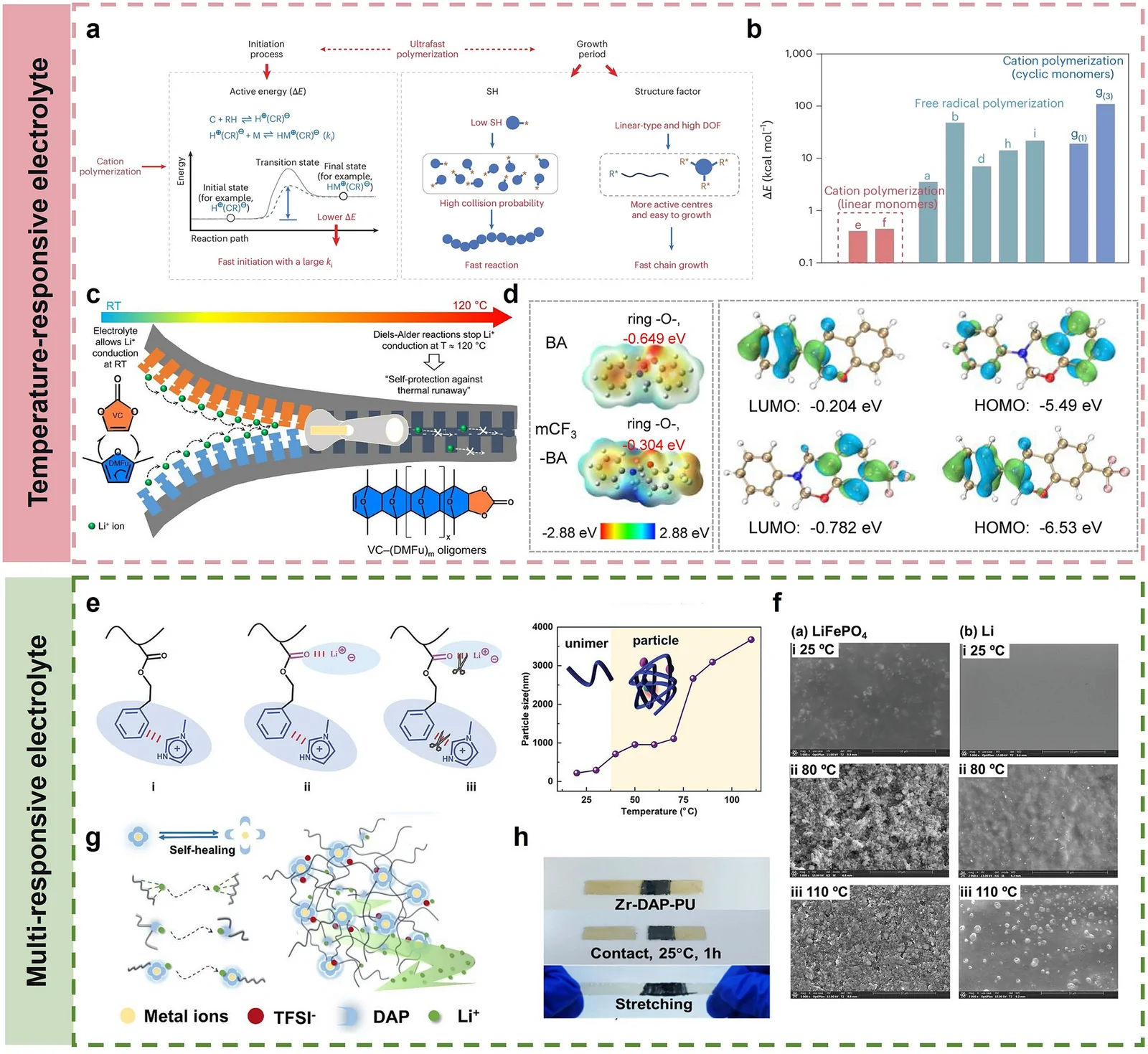
**a** Schematic illustration showing the critical factors affecting the polymerization kinetics of monomers. **b** Activation energy (∆E) of various polymerization types from DFT calculations. **c** Safe battery design with a thermoresponsive electrolyte. Diels-Alder reaction of solvent molecules at elevated temperatures produces oligomers that block separator micropores, leading to Li^+^ conduction shutdown and battery thermal shutdown. **d** Electrochemical stability and performance of BA/mCF_3_-BA additives in Li||NCM811 cells: electrostatic potential (ESP) distributions of BA and mCF_3_-BA. **e** Molecular interplay of 5 wt.% PPhEtMA in C₂MIM[TFSI] under different conditions and repeatable cycles of LCST electrolytes switched at 25 °C and 90 °C. **f** SEM pictures of the Li anodes and LCST electrolytes in smart LMBs at 25, 80 and 110 °C, respectively. **g** Schematic illustration of the MPEs for LIBs. **h** Self-healing process of Zr-DAP-PU. Springer Nature reproduced with permission from; **a**, **b** Ref. [37]. Wiley; **c** Ref. [228], Elsevier; **d** Ref. [36], Wiley; **e**, **f** Ref. [229], Wiley; **g** Ref. [42]

To achieve active safety protection of the electrolyte, temperature-responsive intelligent materials have been widely introduced into electrolyte systems. Zeng et al. [36] designed a thermally responsive benzoxazine derivative (mCF_3_-BA). This additive participates in constructing a stable LiF-rich SEI/CEI at room temperature (Fig. 20d), significantly enhancing battery cycling stability; at elevated temperatures, it rapidly polymerizes to form an insulating network that blocks thermal runaway propagation. Response speed is critical for effective intervention within the transient timescale of thermal runaway. Yang et al. [37] designed an ultrafast thermally responsive electrolyte (TDT). Utilizing the Lewis acid PF_5_ generated from LiPF_6_ decomposition to attack the C=C double bond of triethylene glycol divinyl ether, cationic polymerization is initiated, achieving a liquid-solid phase transition within 5 s at temperatures near the separator melting point, forming a thermal barrier that physically blocks ion transport (Fig. 20a). DFT calculations indicate that the activation energy for cationic polymerization is much lower than that for free radical polymerization, which is the fundamental reason for achieving ultrafast response (Fig. 20b). Ghosh et al. [228] developed a two-component thermally responsive electrolyte composed of vinylene carbonate (VC) and 2,5-dimethylfuran (DMFu). When the temperature rises above 80 °C, VC and DMFu undergo a Diels-Alder cycloaddition reaction, generating oligomers (Fig. 20c), and the electrolyte viscosity gradually increases, entering a warning mode. When the temperature reaches 120 °C, the reaction accelerates, causing a sharp increase in viscosity and bulk resistance, and the battery completely ceases operation, achieving thermal shutdown.

Lower critical solution temperature (LCST) behavior [229] utilizes the temperature-dependent phase separation behavior of poly(phenyl methacrylate) (PPhEtMA) in ionic liquids to achieve reversible, progressive thermal regulation of lithium metal battery electrochemical performance. Above the LCST, the polymer precipitates to form micron-sized particles (Fig. 20e), clogging separator pores and covering the electrode surface (Fig. 20f), physically blocking Li^+^ transport pathways. The temperature dependence of ion-dipole interactions [230] opens new directions for electrolyte/interface modification. By anchoring LiDFOB anions in a PPFA matrix, in situ protection of the lithium metal anode was achieved. At room temperature, the strong PPFA-DFOB⁻ interaction promotes the formation of a stable SEI rich in LiF and LiB_4_O_7_F_2_. At elevated temperatures, thermally triggered anion release and DOL polymerization form a solid electrolyte network, achieving a liquid–solid phase transition. This intelligent response mechanism reduces parasitic heat generation by a factor of 34 and significantly increases the thermal runaway trigger temperature of pouch cells from 112 to 286 °C. Voltage-responsive additives can effectively address overcharge abuse. Overcharge protection additives [231] are activated when the voltage rises abnormally, consuming excess charge through reversible redox reactions or clamping the voltage within a safe range, thereby preventing overcharge-induced thermal runaway.

Self-healing polymer electrolytes can repair interfacial damage. Li et al. [42] constructed a polyurethane electrolyte with a dynamic cross-linked network by coordinating three metal ions, including Zr^4+^, Cu^2+^, and Zn^2+^ with diaminopyridine (DAP) (Fig. 20g). The metal-coordination bonds act as dynamic cross-linking points, enabling self-healing at damaged interfaces through bond dissociation and reassociation (Fig. 20h).

Microencapsulation technology integrates sensing and intervention functions into battery materials. Tong et al. [45] designed microcapsules with aluminum sulfate hexadecahydrate as the core and melamine-urea-formaldehyde resin (MUF) as the shell, which release the hydrate at approximately 100 °C, consuming active lithium at the anode and thereby blocking subsequent violent chain reactions. Adding only 0.25 g of microcapsules to a 1-Ah NCM811 battery increased the thermal runaway trigger temperature by 16.2 °C and reduced the maximum temperature by 117.5 °C. Wei et al. [44] further optimized the microcapsule system, using Ba(OH)_2_·8H_2_O as the core material, increasing the response temperature to 120 °C. Adding only 4 g Ah^−1^ reduced the total reaction enthalpy by 24.6% and significantly lowered the thermal runaway peak temperature. This built-in chemical fuse design concept offers a new pathway for achieving intrinsically safe next-generation batteries without the need for external control.

### Separator

The separator not only serves the fundamental role of physically isolating the positive and negative electrodes, but its thermal stability and ion transport characteristics are also key factors affecting battery thermal safety. Traditional polyolefin separators are prone to shrinkage and melting at high temperatures, leading to internal short circuits; additionally, their nonpolar surfaces exhibit poor compatibility with polar electrolytes, reducing ion transport efficiency, which in turn exacerbates deposition non-uniformity, promotes lithium dendrite growth, and further increases thermal runaway risk.

**Fig. 21:**
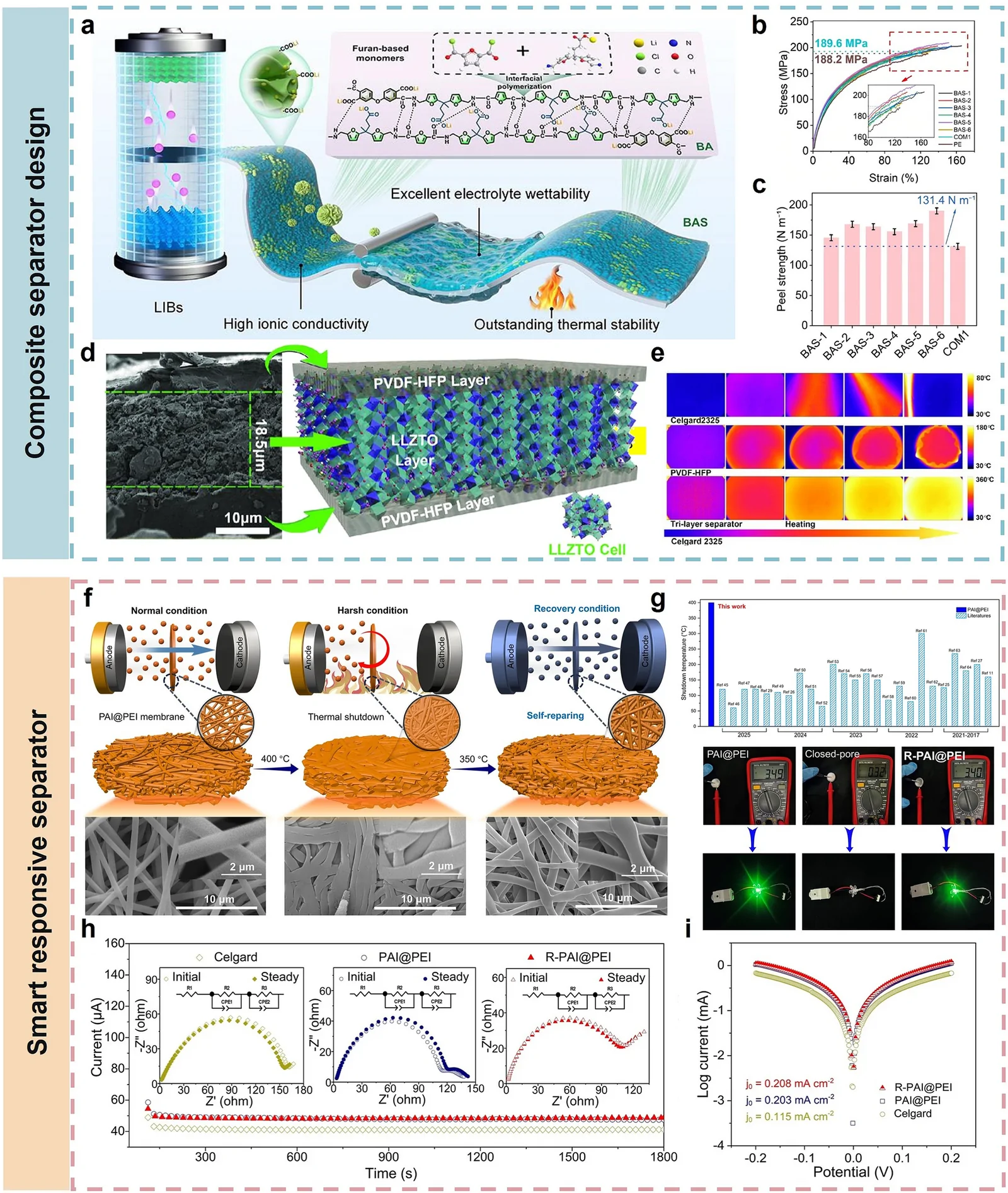
**a** Schematic illustration of the separator fabrication process. **b** Tensile strength. **c** Peel strength. **d** Cross-sectional SEM image and the structural diagram of the trilayer separator. **e** Infrared thermography of Celgard 2325, PVDF-HFP, and trilayer separators. **f** Illustrations of the normal operation, harsh condition, and recovery condition of PAI@PEI separator for LMBs applications. **g** Comparison of shutdown temperature with the state-of-the-art separators and digital images of the DC voltage for the PAI@PEI-based Li||NCM523 batteries before and after heat treatment and corresponding LED lighting. **h** Chronoamperometry plots for a Li||separator||Li cell. **i** Tafel plots for Li plating/stripping of the Li||Li cells. Elsevier reproduced with permission from **a**–**c** Ref. [34], Wiley; **d**, **e** Ref. [35], Springer Nature; **f–i** Ref. [232]

Composite separator design is an effective approach to enhancing thermal stability. Zhu et al. [34] designed a novel separator (BAS) based on bio-based furan aramid (BA) composited with nano-ceramics. By introducing exchangeable carboxyl groups and converting them in situ to -COOLi, a dual lithium-ion transport system combining ion exchange and physical pores was constructed (Fig. 21a). The BAS separator exhibited a thermal shrinkage of less than 1% after heat treatment at 150 °C for 1 h (Fig. 21b). Tensile strength and peel strength were also significantly improved (Fig. 21c). Furthermore, the polar structures of furan rings, amide bonds, and ether bonds effectively improved the compatibility of the BAS separator with the electrolyte, balancing thermal stability and ion transport performance. The mechanical strength of the separator directly determines battery safety under extreme conditions. Feng et al. [35] reported a three-layer composite separator with a PVDF-HFP/LLZTO/PVDF-HFP structure. This separator uses the garnet-type solid electrolyte Li_6.75_La_2_Zr_1.75_Ta_0.25_O_12_ (LLZTO) as the intermediate layer, with gel polymer PVDF-HFP as the outer layers (Fig. 21d). After heat treatment at 300 °C for 1 h, it exhibited no thermal shrinkage, maintaining its original shape and dimensions. Meanwhile, the surface temperature distribution of the three-layer separator was uniform, with no localized hot spots (Fig. 21e). The capacity retention after 200 cycles was 95%, achieving synergistic optimization of thermal stability, flame retardancy, and electrochemical performance.

Smart responsive separators have also become a research hotspot. Li et al. [232] employed a core-shell structured polyamide-imide@polyetherimide (PAI@PEI) nanofiber separator, achieving for the first time reversible thermal shutdown and self-healing of separator pores. This separator leverages the synergistic effect of the thermoplastic PEI core and the thermosetting PAI shell. At high temperatures up to 400 °C, the PEI core melts, and the PAI shell compresses the PEI melt to form a dense structure, closing ion transport channels and achieving thermal shutdown (Fig. 21f). When the temperature decreases to 350 °C, the shape memory effect of the PAI shell drives the reshaping of PEI, restoring the porous structure and regenerating separator functionality. This reversible thermal response characteristic enables the PAI@PEI separator to achieve a record shutdown temperature of 400 °C, significantly higher than existing separators. LED demonstrations showed that the PAI@PEI separator functioned normally both before and after heat treatment (Fig. 21g). Furthermore, after pore recovery, PAI@PEI exhibited excellent lithium-ion transference number, ionic conductivity, and higher nucleation overpotential (Fig. 21h, i), further demonstrating its long-term safety reliability.

Therefore, through surface modification, composite structure design, and the development of novel smart separator materials, continuously improving the thermal dimensional stability, mechanical strength, and ion selectivity of separators is one of the core pathways for suppressing thermal runaway at the material source and enhancing the intrinsic safety of batteries.

### Binder

**Fig. 22:**
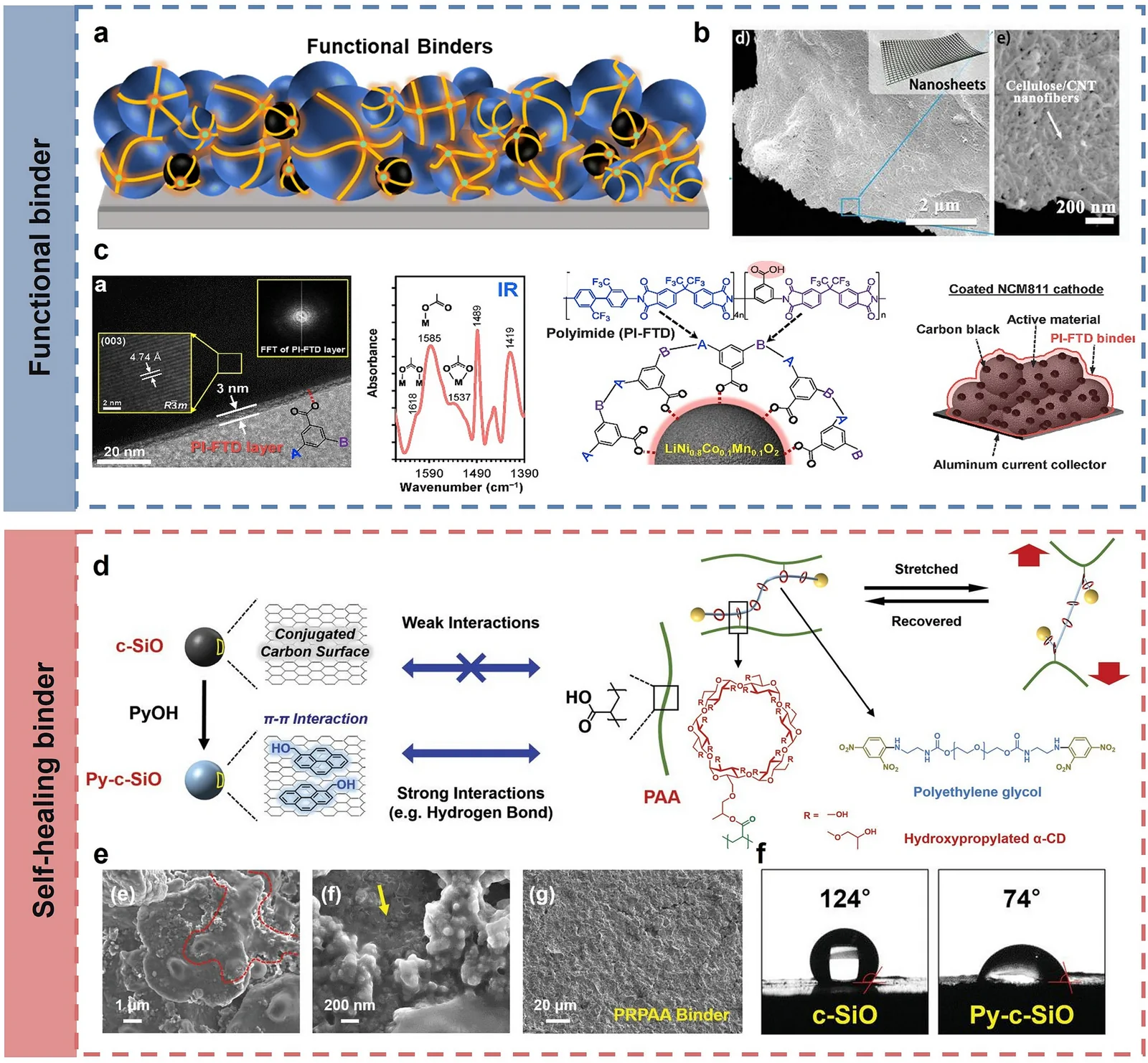
**a** Schematic diagram of functional binders. **b** 3C-binder with their corresponding structure diagrams and SEM images. **c** TEM image and IR spectrum of pristine PI-FTD-NCM811 cathode. Schematic illustration for the chemical binding of PI-FTD to the surface metal oxide of a NCM811 particle, and the structure of the as-coated NCM811 cathode with PI-FTD binder. **d** Schematic illustration of surface modification of c-SiO using PyOH and its interaction with PAA and graphical representation depicting ring-sliding motion in PRPAA and its chemical structure. **e** SEM images of Py-c-SiO-PRPAA electrode after the 1st lithiation. **f** Water contact angle measurements of c-SiO pellet without and with the PyOH functionalization. The Royal Society of Chemistry reproduced with permission from **a** Ref. [234], Wiley; **b** Ref. [235], Elsevier; **c** Ref. [236], Wiley; **d**–**f** Ref. [237]

The binder plays a critical role in connecting active materials, conductive agents, and current collectors within the electrode. Its mechanical properties and interfacial stability directly determine the structural integrity of the electrode, thereby affecting the cycling stability and thermal safety performance of the battery (Fig. 22a).

Although the binder accounts for a relatively small proportion of the total battery mass, its decomposition behavior during thermal runaway has a non-negligible impact on battery safety. Studies have shown that under high-temperature thermal runaway conditions, active lithium in lithiated Gr reacts with hydrogen-containing chemical bonds in the binder molecular structure, leading to bond cleavage and hydrogen gas generation, further intensifying the severity of thermal runaway. Based on this finding, Chen et al. [233] designed a binder with a cyclic structure, PVP, which stabilizes hydrogen bonds in the molecule through the cyclic structure, suppressing hydrogen generation. Additionally, selecting binders without hydrogen atoms can fundamentally eliminate hydrogen generation, thereby improving battery thermal safety.

Traditional PVDF binders face serious challenges in terms of thermal safety. The development of novel functionalized binders has become a research focus. Qian et al. [238] used soluble polyimide (PI) as a cathode binder for lithium-ion batteries. NCM cathodes prepared with this binder exhibited a dense and uniform coating structure, effectively reducing interfacial reactions between NCM particle surfaces and the electrolyte, thereby enhancing battery safety. Pham et al. [236] designed a functional polyimide binder. The carboxyl groups on the binder formed covalent bonds with transition-metal atoms on the surface of NCM811 particles via monodentate ligands, forming an amorphous protective layer in situ on the cathode surface. This chemical bonding mechanism, distinct from the physical adhesion of PVDF, provides stronger structural stability for the electrode (Fig. 22c). Wang et al. [235] prepared a composite binder with a large-size, porous, ultra-thin network structure through esterification-enhanced self-assembly of cellulose and carbon nanotubes. In contrast to the line–point contact mode of traditional PVDF binders, this composite binder combines with active particles in a surface-point wrapping manner (Fig. 22b), forming a continuous three-dimensional conductive framework that integrates conductivity and binding functionality, balancing electrode structural stability and electron transport efficiency.

The introduction of self-healing binders further enhances the service reliability of binders. Peng et al. [239] designed a thermoplastic intrinsically self-healing polymer (TISP) binder. This binder exhibits a very low glass transition temperature, amorphous structure, and low cross-linking density, allowing polymer chains to have high mobility at 40 °C. When cracks form in the electrode, providing heat energy enables intermolecular hydrogen bonds and chain entanglements to rearrange the polymer chains, successfully achieving crack self-healing in high-voltage LiCoO_2_ electrodes. Wei et al. [240] reported a self-healing supramolecular polymer binder additive (TD) based on dynamic covalent disulfide bonds and hydrogen bonds, synthesized by copolymerizing thioctic acid (TA) with diisopropenylbenzene (DIB). This additive possesses three functions: ultrahigh elasticity, rapid self-healing at room temperature, and sulfur-rich CEI film formation, synergistically addressing the issues of cathode volume strain and interface failure from both mechanical buffering and interface regulation perspectives, improving battery cycling stability and thermal safety.

Supramolecular binder networks are beneficial for enhancing the interface stability of silicon anodes. Cho et al. [237] designed a supramolecular binder network based on pyrene-polyacrylic acid-polyrotaxane (PRPAA) for carbon-coated SiO_x_ anodes. Through π-π stacking interactions between pyrene groups and the graphitic carbon layer on the carbon surface, non-covalent functionalization of the carbon surface was achieved, followed by interaction with the PRPAA network via hydrogen bonding, endowing the binder network with high elasticity (Fig. 22d). The reduction in contact angle of PyOH-functionalized c-SiO pellets confirmed the successful introduction of polar hydroxyl groups (Fig. 22f). The high elasticity of PRPAA allowed it to maintain coverage of active particles even after lithiation (Fig. 22e). Through the synergy of multiple non-covalent interactions, this approach addresses the interfacial stability and volume expansion issues of high-capacity anodes.

### Current Collector

The stability of the current collector-active material interface is also critical to thermal safety. During thermal runaway, the active oxygen released from the decomposition of nickel-rich cathodes undergoes a violent aluminothermic reaction with the aluminum current collector, with heat release more than three times that of electrolyte combustion, making it a key factor in battery explosion. Therefore, optimizing the structure and performance of current collectors is of great significance for improving battery thermal safety.

Carbon-coated aluminum foil [244] used as a positive electrode current collector has a surface carbon layer that effectively consumes active oxygen and delays the aluminothermic reaction. This reduces the thermal runaway temperature of a 3-Ah NMC811 battery at full charge from over 600 °C to approximately 300 °C, reduces the maximum temperature rise rate by two orders of magnitude, and significantly prolongs the self-heating time, creating a window for safety warning and intervention.

**Fig. 23:**
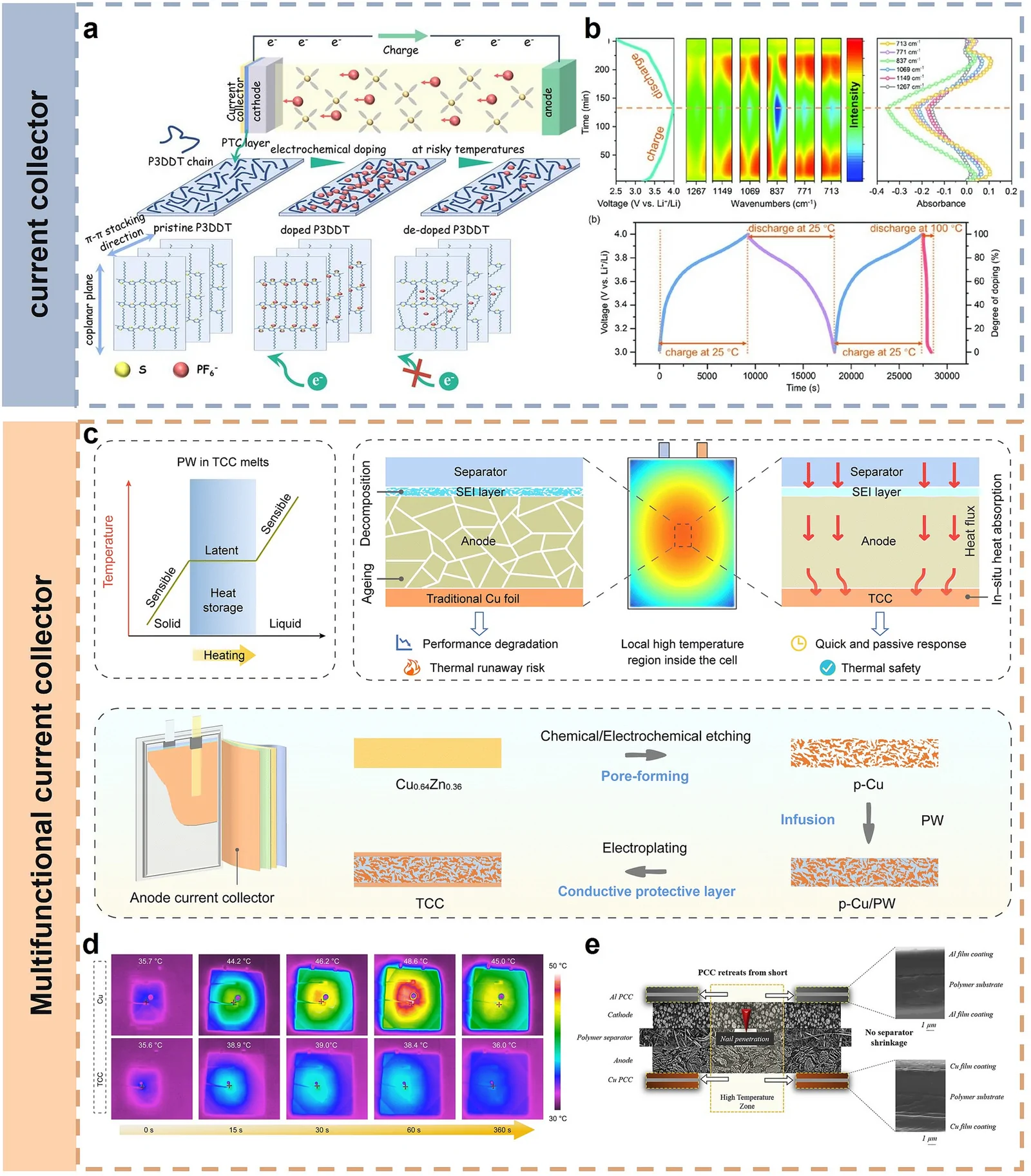
**a** Schematic illustration of the working mechanism of the P3DDT-based thermal shutdown cathode. **b** In situ FT-IR mapping of the P3DDT/Li cell at selected wavenumbers with IR intensity variation during cycling, and charge/discharge curves of the P3DDT electrode at 25 °C and 100 °C. **c** Schematic illustration of PCM-based TCC, including its mechanism for alleviating internal temperature rise in LIBs, the accelerated degradation induced by local high temperatures, and the fabrication process comprising electrochemical etching, infusion, and electroplating steps. **d** Thermal images of pouch-type cells assembled with TCC and Cu after needling. **e** Failure mitigation mechanism of Al PCC and Cu PCC cell during nail penetration. Wiley reproduced with permission from **a**, **b** Ref. [241], The Royal Society of Chemistry; **c**, **d** Ref. [242], Cell Press (Elsevier); **e** Ref. [243]

Temperature-responsive coatings endow current collectors with active-response capability. Li et al. [241] synthesized poly(3-dodecylthiophene) (P3DDT) with long alkyl side chains and used it as a functional coating on the current collector surface (Fig. 23a). The P3DDT layer is extremely thin, making its impact on battery energy density negligible. The p-doped state of P3DDT exhibits high conductivity at room temperature, but its resistance increases sharply above 90 °C due to thermal dedoping, achieving thermal shutdown functionality. In situ FTIR monitoring further revealed the reversible doping/dedoping behavior of PF_6_⁻ during charge/discharge and the associated migration of solvent molecules. The potential-temperature curve showed that the discharge potential of the P3DDT electrode dropped sharply to 3.0 V at 100 °C, confirming the occurrence of thermal dedoping (Fig. 23b).

In studies on metal-coated polymer current collectors, Pham et al. [243] systematically evaluated the performance of aluminum-coated polymer current collectors (Al PCC) in 18,650 cylindrical battery nail penetration tests. These current collectors achieve conductivity by depositing an ultra-thin metal layer on a polymer substrate. When nail penetration triggers an internal short circuit and localized temperature rise, the polymer substrate undergoes thermal shrinkage, physically severing local electron conduction pathways and preventing sustained short-circuit generation (Fig. 23e). The results showed that batteries using Al PCC successfully avoided thermal runaway after nail penetration, maintaining intact battery voltage and retaining an open-circuit voltage of approximately 3.6 V after 8 months. Post-mortem X-ray computed tomography analysis further verified that voids formed in the Al PCC material around the nail penetration path, while the separator remained intact.

Notably, the thermal management function of current collectors, as key components for electron conduction in batteries, has long been overlooked. Peng et al. [242] for the first time integrated phase change materials with current collectors (Fig. 23c), developing a composite current collector (TCC) with self-thermal regulation functionality, combining high heat storage capacity with high thermal conductivity. This enables rapid absorption and buffering of internal battery heat. Infrared thermal imaging showed that TCC effectively suppressed hot spot formation and propagation (Fig. 23d). This design integrates the heat storage function of phase change materials directly into the internal core component of the battery, overcoming the limitations of external thermal management systems, such as response lag and large temperature gradients, offering a new concept for battery thermal safety protection.

### Evolutionary Trajectory of Intelligent Response

Based on the different trigger signals, current research on intelligent response materials can be divided into five categories: temperature response, voltage response, mechanical response, self-healing, and external field response. Temperature response is the most widely studied and most diversely applied intelligent response mechanism, having been realized in nearly all battery components, including cathodes, anodes, current collectors, electrolytes, and separators. Voltage response and mechanical response are more targeted, addressing the key issues of overcharge and dendrite growth, respectively. Self-healing functionality offers a unique solution for performance recovery after mechanical damage, while external field response opens a new dimension for active regulation of battery performance. Although the trigger signals and mechanisms of various intelligent response mechanisms differ, their development follows common evolutionary logic.

**Fig. 24:**
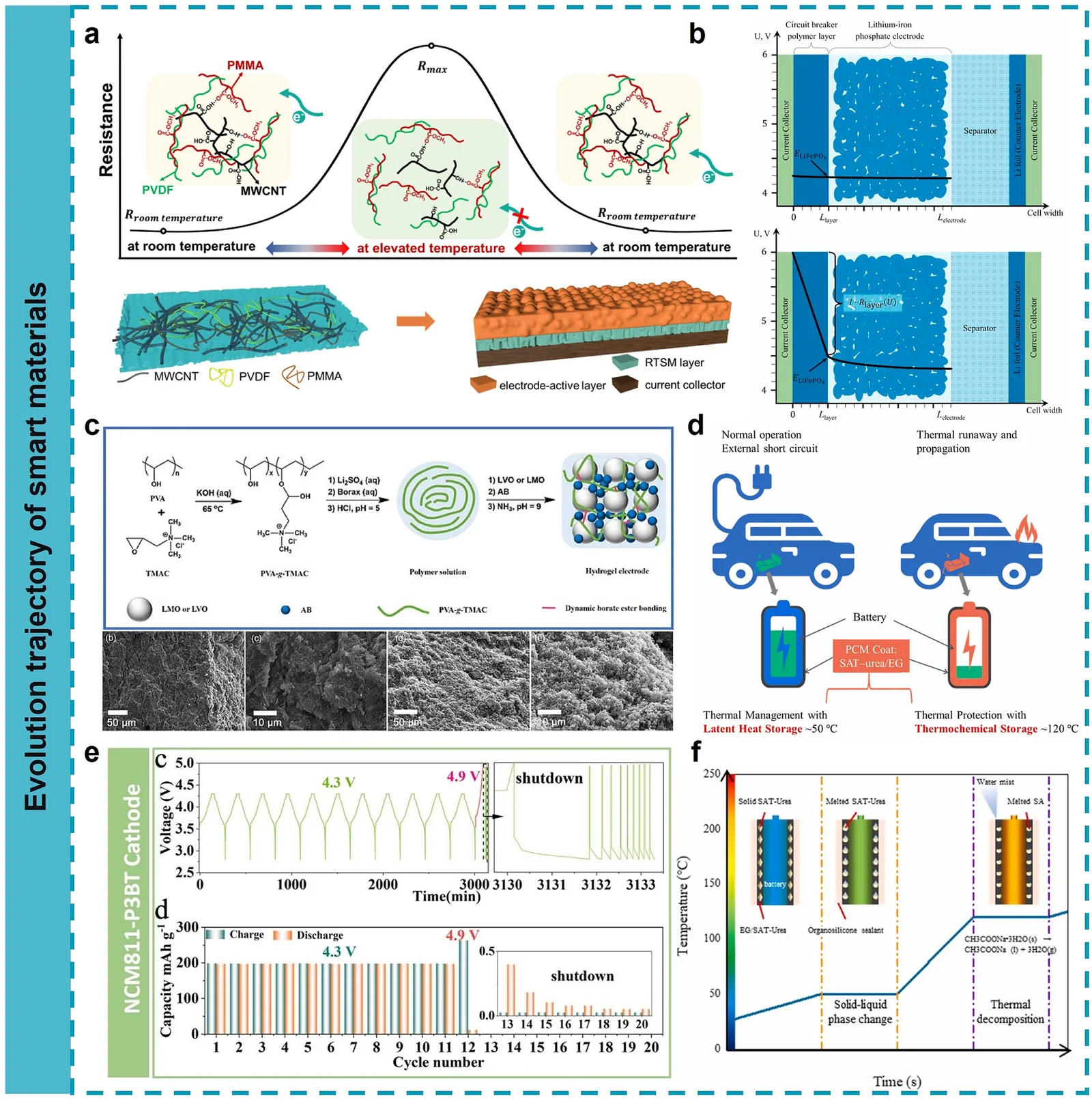
**a** Schematic illustration for structural features and temperature-responsive mechanism of the as-proposed RTSM composite and structure of the temperature-sensitive electrode with an RTSM coating sandwiched between the electrode-active layer and current collector. **b** Schematic representation of the half-cell configuration with protective layer in normal operation conditions and at overcharge. **c** Schematic of self-healable LiV_3_O_8_ and LiMn_2_O_4_ electrode fabrication, with SEM images of the lyophilized electrodes. **d** Schematic diagram of the two-stage thermal storage of the SAT-urea/EG composite PCM for thermal management and protection of Li-ion batteries. **e** Charge/discharge capacities of NCM811 cathode and NCM811-P3BT cathode under deep overcharge conditions. **f** Working principle of thermal management and thermal protection using the inorganic salt hydrate SAT-urea/EG. American Chemical Society reproduced with permission from **a** Ref. [245], Elsevier; **b** Ref. [9], Wiley; **c** Ref. [246] Elsevier; **e** Ref. [39], Elsevier; **d**, **f** Ref. [247]

The evolution from irreversible to reversible is an important direction for intelligent response materials. Early PTC materials were mostly based on thermal expansion or thermal decomposition mechanisms, leading to permanent battery failure once triggered. Zhang et al. [245] dispersed multi-walled carbon nanotubes (MWCNTs) in a mixed polymer matrix of PVDF and polymethyl methacrylate (PMMA). Strong hydrogen bonding between the ester groups on PMMA chains and the hydroxyl and carboxyl groups on MWCNT surfaces enabled reversible reconfiguration of the conductive network. PVDF, due to its large expansion coefficient, undergoes significant volume expansion at high temperatures, causing the MWCNT conductive network to disconnect and resistance to increase sharply. Upon cooling, hydrogen bonding guides the reconnection of MWCNTs, upgrading the PTC effect from a “one-time fuse” to a reversible switch (Fig. 24a). This design concept provides important insights for the reversible response of functional materials.

The shift from macroscopic coatings to the particle level reflects the downward migration of protection mechanisms. Xia et al. [205] pioneered the direct coating of PTC polymers onto the surface of cathode active particles, achieving a particle-level shutdown protection mechanism. Li et al. [206] further extended this strategy to nickel-rich ternary material systems, coating NCM811 with P3OT composite material, endowing each active particle with overheat self-protection. Monteiro et al. [207] achieved electrode-level thermal shutdown at lower cost through the physical mechanism of thermally expandable microspheres. This downward scaling from macroscopic to microscopic brings protection mechanisms closer to the source of thermal runaway.

The evolution from functional coatings to intrinsic material properties represents the ultimate integrated form of intelligent response. Thermal response functionality is typically integrated into electrode structures through coatings or encapsulation, but these strategies often require the introduction of additional functional components into the electrode, potentially sacrificing energy density or increasing process complexity. Li et al. [209] directly endowed the cathode material itself with thermal response functionality, using p-type conductive polymers such as P3OT and P3BT as active materials and constructing P3OT@CNT and P3BT@CNT nanocomposites by compositing with CNTs. This achieved a dual functionality where the electrode material serves both as an energy storage active material and as an overheat self-protection switch.

The evolution from single response to multimodal integration represents the development trend of intelligent materials. Beletskii et al. [38] and Li et al. [39] developed voltage-responsive PTC materials for LFP and NCM811 systems, respectively, integrating overcharge protection and thermal protection functionalities into a single functional layer. The poly[Ni(CH_3_OSalen)] complex exhibits high conductivity only within the normal operating potential range of 3.0–4.2 V. When the potential exceeds the overcharge threshold of 4.2 V, its resistance sharply increases by more than 50 times, rapidly driving the battery voltage to the cutoff voltage through ohmic polarization, interrupting the overcharge current (Fig. 24b). P3BT undergoes p-doping under normal charge/discharge conditions, maintaining good electronic conductivity to ensure unimpeded electron transport between the active material and the current collector. When the battery experiences deep overcharge (≥ 4.9 V), P3BT undergoes irreversible overoxidation, losing electrochemical activity and conductivity, thereby permanently cutting off the electron transport pathway, rendering the battery completely inactive and preventing thermal runaway (Fig. 24e). Hao et al. [246] constructed a self-healing battery based on dynamic boronate ester bonds, achieving autonomous repair of mechanical damage, complementing thermal response functionality (Fig. 24c). Lee et al. [248] pioneeringly reported the accelerating effect of light radiation on the charging process of LMO cathodes, opening a new dimension for external field response functionality.

Multifunctional integration of intelligent materials is the future development direction. Lin et al. [247] developed an inorganic salt hydrate composite material based on sodium acetate trihydrate-urea/expanded Gr (SAT-urea/EG), which possesses dual functions of latent heat and thermochemical energy storage, achieving two-stage adaptive response to temperature (Fig. 24d). During normal operation, when the battery temperature reaches approximately 50 °C, the material absorbs the waste heat generated during operation through phase change, providing thermal buffering. Once the temperature suddenly rises to the thermal runaway trigger point, the material initiates a thermal decomposition reaction, instantly absorbing a large amount of heat (Fig. 24f), intervening in the thermal runaway chain reaction at its source.

In summary, the safety design of individual cells is undergoing a profound evolution from traditional passive protection toward active-response intelligent materials. Passive protection enhances intrinsic stability through material modification, delaying thermal runaway initiation at the source. Active response, through sensing signals such as temperature, voltage, and stress, spontaneously initiates protection mechanisms, achieving precise intervention at the critical threshold of thermal runaway. These two types of strategies are not mutually exclusive but complement and synergize with each other: passive protection buys time for active response, while active response compensates for the limitations of passive protection. Future development directions will focus on multimodal sensing and coordinated response, response kinetics optimization, reversibility and cycle stability enhancement, system-level integration, and low-cost large-scale preparation. Through the synergy of passive protection and active response, and the integration of intrinsic material safety with intelligent response, next-generation energy storage systems are expected to achieve true intrinsic safety.

Table 5 systematically summarizes the main strategies and mechanisms of thermal safety technologies for cathodes, anodes, electrolytes, separators, binders, and current collectors from the perspectives of passive protection and active response.

**Table 5:** Summary of thermal protection technologies at the cell level

Component	Protection type	Strategy category	Core mechanism	Reference
Cathode	Passive protection	Crystal structure regulation	Suppresses lattice oxygen loss, enhances structural stability	[199]
		Elemental doping	Strengthens Ni-O bond energy, suppresses oxygen vacancies	[201, 202]
		Surface coating	Forms physical barrier to block oxygen release and electrolyte attack	[30] [31]
		Surface electronic state modulation	Stabilizes surface electronic states, inhibits thermally induced phase transition and oxygen release	[200]
		Electrode microstructure design	Disperses impact stress, increases internal short-circuit resistance	[203]
	Active response	Temperature-responsive cathode	Autonomous shutdown of electron/ion transport under overheating conditions	[205–207]
		Structural self-healing cathode	Replenishes lithium vacancies, suppresses irreversible phase transition	[208]
		Intrinsically responsive materials	Combines electrochemical activity with overheat self-protection function	[209]
Anode	Passive protection	Surface coating and interface modification	Suppresses high-temperature side reactions, stabilizes SEI	[210]
		3D structure design	Uniform lithium deposition, suppresses dendrite	[211, 212]
		Bio-inspired structure design	Multi-level buffer structure	[213, 215]
		Hierarchical structure design	Maintains dynamic electrical contact, accommodates volume changes	[214, 217, 218]
	Active response	Stress-potential coupling regulation	Stress-induced potential redistribution, inhibits preferential dendrite growth	[40]
		Piezoelectric adaptive regulation	Converts stress into built-in electric field, adaptively regulates lithium deposition	[41]
		Pressure-adaptive composite anode	Converts deposition stress into counter-pressure, suppresses dendrites	[221]
		Self-healing anode	Self-heals structural damage	[43, 222, 223]
		Temperature-responsive anode	Overheating-induced expansion disrupts conductive network	[219, 224]
		Adaptive electrochemical grinding	Converts pulverization into controlled grinding	[220, 225]
Electrolyte	Passive protection	Fluorinated electrolyte	Suppresses PF_5_/POF_3_ catalysis	[32, 227]]
		Flame-retardant additives	Delays combustion, reduces burning intensity	[33]
		Solvation structure regulation	Constructs a dual-layer SEI	[33]
	Active response	Thermally responsive additives	High-temperature polymerization or phase separation blocks ion transport	[36, 228, 229]
		Ultrafast thermally responsive electrolyte	Ultrafast liquid-solid phase transition, rapidly cuts off ion pathways	[37]
		Voltage-responsive additives	Consumes excess charge during overcharging	[231]
		Self-healing polymer electrolyte	Self-repairs interface damage	[42]
		Microencapsulation	Thermally triggered release of active agents consumes active lithium	[44, 45]
Separator	Passive protection	Composite separator	Improves thermal dimensional stability, enhances mechanical strength	[34, 35]
	Active response	Smart responsive separator	Reversible thermal shutdown and shape-memory self-repair	[232]
Binder	Passive protection	Functionalized binder	Covalently bonds to active particles, forms 3D conductive network	[235, 236, 238]
		Hydrogen evolution suppression	Stabilizes hydrogen bonds, reduces hydrogen generation	[233]
	Active response	Self-healing binder	Thermally induced crack self-repair, buffers volumetric strain	[239, 240]
		Supramolecular binder network	Multi-network synergy, provides high elasticity and interface stability	[237]
Current Collector	Passive protection	Carbon coating	Consumes active oxygen, delays aluminothermic reaction	[244]
	Active response	Temperature-responsive coating	Thermally induced resistance surge, enables thermal shutdown	[241]
		Metal-coated polymer current collector	Heat-induced polymer shrinkage physically severs local conductive pathways	[243]
		Phase change composite current collector	High heat storage capacity combined with high thermal conductivity	[242]

### Tailored Protection Strategies for Emerging Battery Systems

#### Multi-dimensional Modification and Protection Strategies for Sodium-Ion Batteries

In addressing the distinctive failure mechanisms of SIBs, recent cutting-edge research has expanded safety mitigation roadmaps across five critical dimensions—anode, cathode, electrolyte, separator, and thermal management—furnishing a rich technical repository for engineering high-safety, long-lifespan SIB configurations.

In terms of anode material modification, Jia et al. [249] designed a carbon aerogel-encapsulated bimetallic selenide (NiCoSe@GC) anode framework, leveraging the porous carbon skeleton to cushion massive volumetric expansion and utilizing bimetallic synergetic effects to accelerate reaction kinetics. Within full-cell configurations, the Na/NiCoSe@GC half-cells advanced their T_0_ and T_1_ thresholds by 48.2 and 10.3 °C, respectively, alongside a 450% enhancement in activation energy, delivering the first systematic proof of the positive impacts of rational anode spatial tailoring on cell thermal safety.

For cathode material modification, Qian et al. [250] developed a polyethyleneimine (PEI) aqueous binder to displace the conventional PVDF/NMP processing paradigm. The compiled NVP/C, PEI cathode delivered a capacity of 134.6 mAh g^−1^ at 0.1C, and ARC profiling demonstrated that this binder chemistry shifts the self-heating onset upward while decreasing the total reaction enthalpy compared to PVDF-bound counterparts. Cheng et al. [251] introduced a dual-modification strategy using Y_2_O_3_ and rGO on NVP, constructing a multifunctional “electronically conducting, ionically transporting, and stress-buffering” interfacial shell. This architecture yielded a fourfold increase in electronic conductivity and maintained a capacity retention of 91.5% after 400 cycles at 45 °C (contrasting with a dismal 38.2% for pristine NVP). Liu et al. [251] capitalized on the negative temperature coefficient (NTC) material Pr_6_O_11_ to modify NVP. By exploiting its unique high-temperature impedance-drop trait to optimize high-temperature charge transfer, the modified cathode sustained a 78.97% capacity retention after 1000 cycles at 8C under 45 °C, far outstripping the 14.99% baseline of pristine NVP.

Regarding electrolyte engineering, Zhang et al. [252] developed an in situ polymerizable, non-flammable electrolyte (PNE) formulated on triethyl phosphate (TEP). This system leverages the high-temperature thermal polymerization of TEP to construct physical blockades that arrest electrode crosstalk, enabling Ah-scale cylindrical cells to successfully pass severe nail penetration tests and sustain high-temperature ARC heating up to 300 °C without undergoing TR. Tran et al. [108] formulated a localized high-concentration electrolyte (LHCE) composed of NaFSI/diglym/TTE. By exploiting an anion-enriched solvation structure to build inorganic-dominated SEI/CEI cross-layers, they suppressed the full-cell outgassing to a minimal 0.26 mL Ah^−1^ and delayed the ARC self-heating onset from 101.5 to 126.5 °C. Kausthubharam et al. [253] emphasized the philosophy that “non-flammability does not equate to thermal safety,” calling for the establishment of a systematic evaluation framework correlating electrolyte formulations, SEI characteristics, and ultimate thermal safety limits. Zhang et al. [254] introduced a chloroform additive into carbonate ester electrolytes to achieve complete flame retardancy, though noting the necessity of monitoring its pitting corrosion risks toward the aluminum current collectors.

In separator modification, Du et al. [255] reported a multifunctional polyimide (PI) separator. The integrated imide rings chemically coordinate with PF_5_ intermediates to suppress corrosive HF generation and alleviate transition-metal dissolution, while concurrently elevating the Na^+^ transference number from 0.42 to 0.71, enabling coin cells to preserve 80% capacity over 1200 cycles at 50 °C. At the thermal management level, Ren et al. [256] systematically compared the mitigation efficacy of fluorinated fluid immersion cooling across layered oxide and polyanion SIB Chemistries. For Na_1/3_Fe_1/3_Mn_1/3_O_2_ (NFM) cells, the cooling fluid reduced the maximum peak temperature (T_max_) by 53.4% and compressed the comprehensive risk index from 98% down to 63%. However, for Na_3_V_2_(PO_4_)_3_ (NFP) configurations, the vented H_2_ proportion surged from 12.3% to 43.3%, which conversely exacerbated secondary explosion risks. This highlights that the efficacy of immersion cooling management is heavily contingent upon the underlying cathode crystal chemistry.

Although these multi-dimensional modifications exhibit exceptional thermal control at the material level, future breakthroughs must conquer the intrinsic trade-off between safety gains and kinetic penalties. For instance, localized high-concentration electrolytes (LHCEs) or in situ high-entropy SEIs, while effectively retarding the self-heating onset, frequently penalize low-temperature power densities or escalate raw material costs due to expensive fluorinated diluents. Similarly, the starkly antithetical explosion behaviors induced by fluid immersion cooling across layered oxide versus polyanion frameworks reveal that macro-scale thermal management is by no means a universal “panacea”; its architecture must be strictly decoupled and synchronized with the underlying crystal chemistry and specific outgassing dynamics of the cell over its entire lifespan.

#### Multi-dimensional Modification and Protection Strategies for Solid-State Batteries

To resolve the critical interfacial instabilities and severe thermal hazards of ASSBs, recent research has yielded pivotal breakthroughs across the dimensions of interfacial engineering, stress regulation, and structural design.

In the realm of high-entropy inorganic interphase design, Luo et al. [257] strategically formulated a high-entropy anionic electrolyte to derive an in situ hybridized high-entropy SEI on a hard carbon anode, comprising an array of inorganic building blocks (NaF, NaCl, Na_3_P, Na_2_S, and Na_4_B_2_O_5_). Within this architecture, the chemically inert components provide thermal passivation, while the active constituents deliver low-barrier diffusion pathways for rapid ion hopping. ARC evaluations proved that this high-entropy inorganic SEI advanced the T_1_ of compiled pouch cells from 52.4 to 161.9 °C, while simultaneously compressing the maximum temperature rise rate from a catastrophic 1628.5 °C min^−1^ down to a placid 17.4 °C min^−1^.

Regarding stack pressure regulation of thermal stability, Hasan et al. [258] systematically quantified the impacts of mechanical stack pressure on the thermal kinetics of sulfide-based ASSBs. Within Li|LSPS|Li symmetric cell configurations operated under 0 MPa, the transient maximum self-heating rate soared to a severe 4233.9 °C min^−1^. Crucially, when the external stack load was scaled up to 20 MPa, the characteristic T_2_ point was pushed back from 194.8 to 303.8 °C, and the maximum self-heating velocity dropped sharply to 2.5 °C min^−1^. XPS and FIB-SEM diagnoses revealed that high stack loads effectively suppress the chemical segregation of reactive Li-Sn alloys and reduced metallic Sn species. Full-cell thermal safety phase diagrams indicated that elevating the stack pressure from 0 to 20 MPa advances the T_2_ from 213.4 to 278.6 °C, while curbing the T_3_ from 512 °C down to 405 °C.

For interfacial dual-conductive layer engineering, Zheng et al. [259] leveraged a high-temperature semi-cyclized polyacrylonitrile (sc-PAN) strategy to construct a soft gel coating simultaneously delivering high ionic and electronic conductivities on the boundaries of high-nickel LiNi_0.8_Co_0.1_Mn_0.1_O_2_ (NCM811) cathode particles. This architecture accelerated interfacial Li^+^ and electron transfer, with ARC profiling recording a solid-state cell self-heating onset of 221.4 °C, markedly superior to the 148 °C baseline of liquid references. Zheng et al. [260] further implemented an in situ polymerization pathway to construct a partially cyclized polyacrylonitrile (cPAN) shell. DFT calculations revealed that the formed Ni–N coordination bonds elevate the migration energy barrier of nickel ions from 2.83 eV up to 3.36 eV, thereby successfully locking cation mixing and sustaining stable cycling even under an extreme thermal stress of 100 °C.

In the dimension of dipole-matched polymer electrolytes, Song et al. [261] incorporated N-CFF acting as a smart nucleating agent within a PVDF-HFP-based polymer electrolyte matrix. This additive directed the polymer matrix to assemble into a highly dense and homogeneous microscopic topology, boosting the room-temperature ionic conductivity to 6.2 × 10^–4^ S cm^−1^ and elevating the Li^+^ transference number (t_Li+_) from 0.36 to 0.57. The resulting Li||LFP full cell delivered a 90% capacity retention over 3000 cycles at 1C, with ARC profiling verifying an upward shift in the self-heating onset from 90 to 160 °C.

Targeting competitive-diffusion stabilized cathodes, Dai et al. [262] proposed a chemical competitive diffusion strategy utilizing Ta and Nb to concurrently stabilize the bulk lattice of NCM811 and its rigid interface against sulfide electrolytes. Ta doping successfully lodged into the bulk crystal lattice to suppress oxygen evolution under high thermal stresses, while surface-segregated Nb formed a piezoelectric LiNbO_3_ buffer shell to optimize interfacial ion transport. The compiled M-NCM/LSPS all-solid-state full cell achieved a capacity retention of 97.3% over 120 cycles under a stringent high-voltage ceiling of 4.5 V at 0.2C.

Looking into the future, the research paradigm for ASSB safety should shift from static single-component modifications to quantifying how dynamic interphase evolution rules govern the thermodynamic limits of thermal failure. It is noteworthy that while external stack pressure effectively suppresses the malicious propagation of mixed conducting interphases (MCIs) during the thermal runaway incubation window, heavy mechanical clamping hardware severely penalize the system-level gravimetric and volumetric energy densities. Consequently, how to convert macro-scale external mechanical constraints into internal “self-regulating elastic stress axes” within the cell architecture, and how to develop smart tri-in-one topological micro-coatings that simultaneously deliver electronic insulation, ionic superconduction, and thermochemical passivation under extreme high-voltage/high-temperature stresses, remain the ultimate path toward intrinsically safe, high-energy–density ASSBs.

## System Integration and Engineering Protection

In the transition from cell-level material modification to system-level thermal safety protection, optimization of intrinsic material properties provides foundational support for efficient thermal management and propagation barrier technologies. By enhancing the thermal stability and thermophysical properties of key components at the material level, thermal runaway initiation can be delayed and heat accumulation reduced. Meanwhile, the introduction of functional materials with high thermal conductivity, high heat storage capacity, and thermal insulation properties lays a functional foundation for thermal management design within cells and between modules. In practical applications, lithium-ion batteries are typically used in modular configurations, making research on thermal runaway propagation within modules particularly important.

### Efficient Thermal Management and Propagation Barrier Technologies

Thermal management is a key strategy for preventing thermal runaway in lithium-ion batteries. Its technical approaches are mainly divided into two categories: enhanced heat dissipation and thermal propagation blocking. Enhanced heat dissipation aims to improve the heat dissipation efficiency of the battery itself, with typical methods including liquid cooling plates, phase-change materials, and heat pipes. Thermal propagation blocking focuses on suppressing the spread of thermal runaway within battery modules, primarily through the application of fire-resistant insulation materials and the design of physical barriers.

To achieve effective thermal propagation blocking, a deep understanding and quantitative prediction of thermal propagation mechanisms are first required. Zhang et al. [263] proposed a thermal runaway propagation uncertainty quantification framework. Through high-dimensional surrogate modeling and global sensitivity analysis, they identified SOC, phase-change materials (PCMs), thermal conductivity, and cell spacing as key factors dominating TRP uncertainty. The study found that when SOC is in the critical range of 40%–55%, the system is extremely sensitive to parameter variations, with TRP occurrence probability and temperature exhibiting high dispersion and peak uncertainty, revealing the existence of a nonlinear abrupt threshold in the safety state of battery systems.

To overcome the limitations of empirical design, Xia et al. [264] proposed a universal theoretical criterion based on energy balance: the thermal runaway propagation number (TRPN), achieving a transition from qualitative protection to quantitative prediction. TRPN integrates factors such as battery heat generation kinetics, geometric thickness, internal thermal conductivity, external heat dissipation intensity, and thermal coupling between cells. Its critical condition, TRPN < 1, constitutes the theoretical boundary for whether thermal propagation occurs. Mohaddes and Wang [265] established a dimensionless theoretical framework for module-scale thermal runaway propagation. For simplified modules with stacked pouch cells, quasi-one-dimensional modeling was used to abstract the complex propagation process into a control problem governed by several key dimensionless parameters. The core output is the average mass consumption rate, which directly relates to the overall heat release power and fire hazard of the module. Based on such models, more targeted propagation blocking strategies can be developed.

On this theoretical foundation, this section systematically elaborates on research progress in efficient thermal management and propagation blocking from three dimensions: enhanced heat dissipation design, thermal propagation barrier technologies, and multifunctional integrated thermal management materials.

#### Enhanced Heat Dissipation Design

To ensure the thermal safety and performance of battery systems under high-power, fast-charging, and extreme environmental conditions, enhanced heat dissipation design has become a core aspect of system thermal management. It primarily relies on two approaches: active thermal management and passive thermal management. Active thermal management achieves precise temperature control by consuming external energy, including liquid cooling [266], air cooling [267]**,** immersion cooling [268], spray cooling [269], and water mist [270]. Passive thermal management technologies, such as phase-change materials, heat pipes, and thermally conductive structural components, achieve thermal buffering and temperature uniformity without additional energy consumption through the inherent heat storage and superconducting properties of materials. Current research and practice are increasingly focusing on the organic coupling of active and passive technologies to construct multiscale thermal management systems with rapid response, controllable energy consumption, and high safety and reliability.

##### Active Thermal Management

At the system level, efficient active thermal management is a key engineering approach for building a firewall against thermal runaway propagation. However, there is an order-of-magnitude gap between active heat dissipation power and the instantaneous heat release power during thermal runaway. To reduce this gap, Xu et al. [271] proposed and validated an aluminum microchannel cooling system integrated on the side of individual cells. The study found that microchannel cooling cannot halt an already triggered thermal runaway at the cell level but can effectively prevent its propagation to adjacent cells at the module level.

**Fig. 25:**
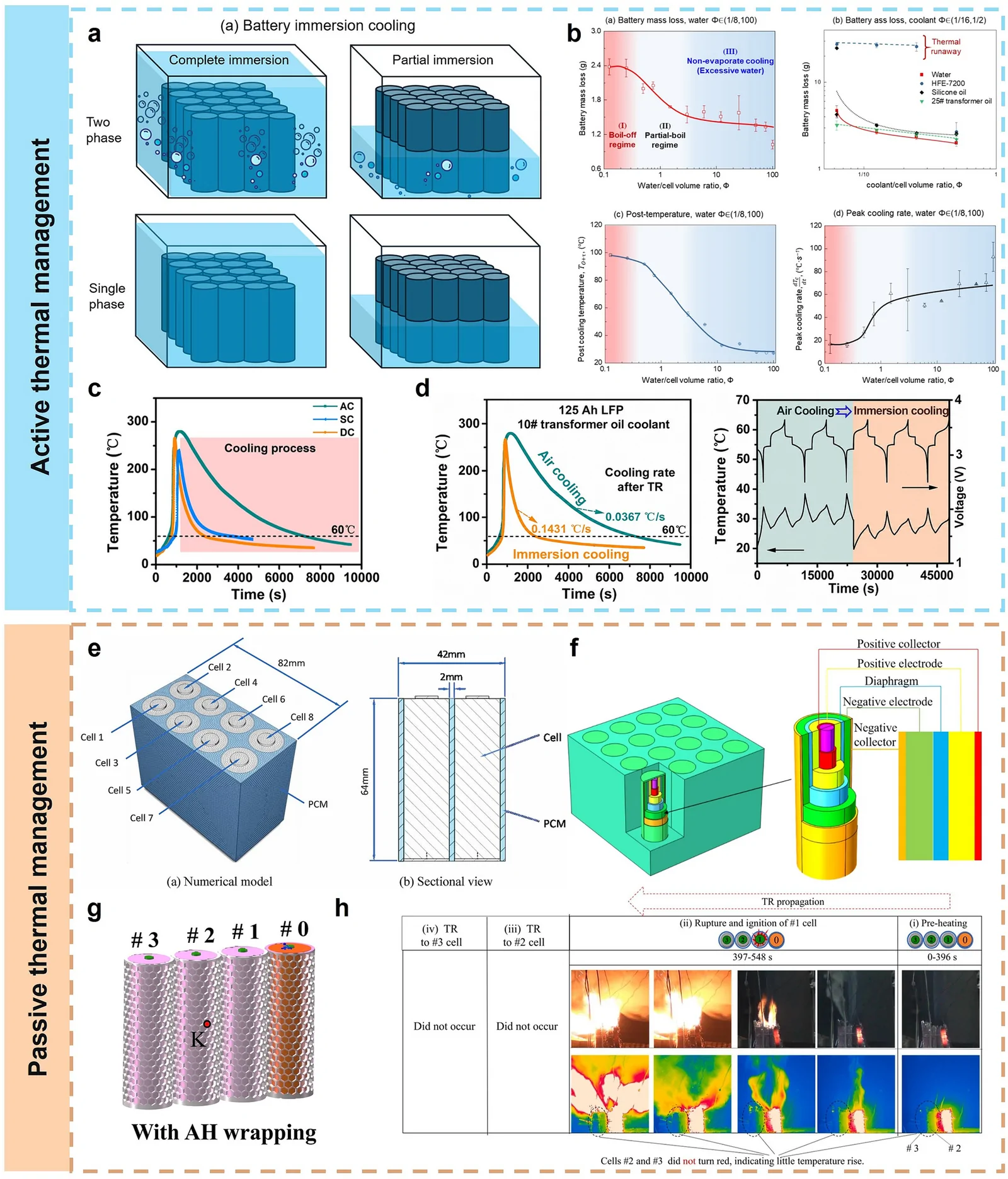
**a** Schematics of different types of battery immersion cooling system. **b** Effect of water-battery volume ratio on preventing thermal runaway battery mass loss, battery mass loss vs. coolants, post-cooling cell peak temperature, and peak cooling rate of battery. **c** Temperature variation on the top surface of the battery under three cooling methods. **d** Voltage and temperature curves vs time of an LFP battery under AC to DC during the charging/discharging power of 1P and selected data. **e** Battery module with PCM. **f** Schematic diagram of battery module and internal structure of battery. **g** Conceptual design of the cell module with aluminum honeycomb (AH) wrapping. **h** Experimental photographs and infrared (IR) images of the TR process for Group VI. Elsevier reproduced with permission from **a**, **b** Ref. [272], American Chemical Society; **c**, **d** Ref. [268], Elsevier; **e** Ref. [274], Elsevier; **f** Ref. [275], Elsevier; **g**, **h** Ref. [276]

In the area of active liquid cooling, Bai et al. [268] investigated the application of immersion cooling in thermal runaway suppression and thermal management of large-capacity LFP batteries, systematically comparing the effects of air cooling, static immersion cooling, and dynamic immersion cooling (Fig. 25c). The study showed that dynamic immersion cooling achieved the highest cooling rate, which was twice that of static cooling and four times that of air cooling. This is because the system achieves uniform heat dissipation through medium flow, effectively avoiding heat accumulation and propagation (Fig. 25d).

In system-level thermal safety design, early active suppression strategies aim to intervene before the thermal runaway chain reaction is fully established. Zhang et al. [272] utilized the golden time window after battery pressure relief, employing micro-volume liquid immersion cooling (Fig. 25a). The study showed that even a minimal amount of coolant can effectively curb the self-heating process, with the effect depending on the precise matching of coolant type and dosage. Three mechanisms (boiling depletion, partial boiling, and non-evaporation) were identified (Fig. 25b). A five-dimensional evaluation system compared four immersion coolants, indicating that water has advantages in comprehensive performance.

When battery thermal management systems cannot meet heat dissipation demands under extreme conditions, emergency spray cooling [269] serves as an enhanced heat dissipation method. Utilizing the dual mechanisms of evaporative heat absorption from water droplets and oxygen isolation from water vapor, initiating spray during thermal runaway Stage II can effectively suppress the chain reaction. Studies have shown that water mist (FWH) cooling can improve cooling efficiency by up to 46% compared to air cooling. However, when the net droplet content in the mist field is below 6.2 g m^−3^, the cooling effect is significantly weakened. Based on TR cooling control classified into three levels and with 240 °C identified as a conservative temperature threshold for hazard control, the TR control capability of the FWH cooling system was demonstrated [273].

However, existing studies mostly place nozzles close to the batteries, neglecting the counteracting effect of high-speed gas flow from safety valves on the water mist. Li et al. [270] simulated a more practical long-distance (1 m) spraying scenario. For LFP batteries, water mist was effective both before and after safety valve opening, and a critical heat accumulation density threshold of 155 kJ Kg^−1^ was identified. For large-capacity, multi-cell battery systems, thermal runaway evolution exhibits holistic characteristics, with the disaster process dominated by continuous gas generation from internal cells. The time window from anomaly development to overall failure is extremely short, approximately 15 s. Under these conditions, the water mist system was proven to effectively remove over 65% of the accumulated heat in the battery [277], demonstrating strong suppression potential. However, its efficient application requires intelligent integration with monitoring systems capable of capturing early key signals for precise activation. In summary, water mist cooling offers advantages such as fast response, strong heat dissipation, and fire suppression capability, but faces challenges in engineering response speed, low droplet utilization efficiency, and system reliability.

##### Passive Thermal Management

The core principle of PCMs lies in their ability to absorb or release large amounts of latent heat during phase transitions, thereby effectively controlling battery temperature fluctuations [70]. Ni et al. [274] directly attached paraffin PCM to the surface of 18,650 cylindrical cells (Fig. 25e). During nail penetration-induced thermal runaway, PCM significantly reduced the maximum temperature of the triggered cell and slowed its temperature rise rate. More importantly, by absorbing heat and maintaining near-constant temperature during the phase-change process, PCM effectively prevented thermal runaway propagation to adjacent cells. Heat pipes, as highly efficient heat transfer devices, promote rapid heat transfer through the phase change cycle of internal working fluid, thereby reducing the maximum surface temperature of the battery and improving temperature uniformity [278].

In terms of structural design innovation, inspired by the physical concept of thermal invisibility, Sun [275] proposed a novel thermal cloak structural design (Fig. 25f). Individual cells were sequentially wrapped with an inner PCM layer and an outer high-thermal-conductivity metal layer. The inner PCM efficiently absorbs heat during normal operation and early thermal runaway, delaying temperature rise. The outer high-thermal-conductivity metal establishes rapid radial heat dissipation channels during extreme heat generation, achieving synergy between internal heat accumulation and external heat dissipation. Simulation and experiments showed that this structure significantly improves temperature field uniformity and delays thermal propagation. However, the introduction of the metal layer increases system weight and complexity, and its integration scheme and energy density loss must be carefully balanced. Structured expanded surface designs enhance heat dissipation efficiency by increasing the effective heat dissipation area of the battery. Wang et al. [276], inspired by honeycomb structures, used lightweight, high-thermal-conductivity aluminum honeycomb structures as expanded heat transfer surfaces external to the battery (Fig. 25g). Under natural convection, this structure reduced the maximum battery temperature by approximately 5%; under forced convection, the temperature reduction reached 17.1%, demonstrating excellent convective heat transfer enhancement and thermal propagation blocking effects (Fig. 25h), while also improving the mechanical impact resistance of the module. However, the overall thermal conductivity of the aluminum honeycomb is relatively low, which may limit heat dissipation capability under high-rate discharge or high-temperature environments.

Optimizing the combination of these strategies enables the most effective thermal management under various operating conditions. Among them, the innovative application of composite phase-change materials (CPCMs) offers new approaches. Traditional PCMs suffer from low thermal conductivity, leakage, and structural instability. Li et al. [279] designed a dual-network composite PCM based on aerogels. This material combines the high-thermal-conductivity network of graphene nanoplatelets (GNP), the phase change heat storage capability of hydrogels, and the structural support of nanofiber frameworks, achieving triple functional integration of “thermal conduction-heat storage-structural support.” This provides an all-weather thermal management solution with excellent cycling durability for batteries. Furthermore, the geometric design of composite PCMs is crucial to their performance. Morali [280] showed that a 1 mm thick PCM can effectively reduce battery temperature under high-rate discharge conditions, but the material thickness must be balanced against weight/volume fraction in system integration. Gao et al. [281] developed a thermal management system combining heat pipes with PCM. Heat pipes efficiently dissipate heat, while PCM absorbs and stores this heat. This synergy demonstrated excellent thermal performance under various discharge rates, highlighting the advantages of multiphysics coupled heat dissipation design.

#### Thermal Propagation Barrier Technologies

Thermal propagation barrier technologies primarily achieve their goals through two approaches: thermal insulation materials and structural isolation, aiming to delay or block the spread of thermal runaway between individual cells and modules.

##### Thermal Insulation Materials

**Fig. 26:**
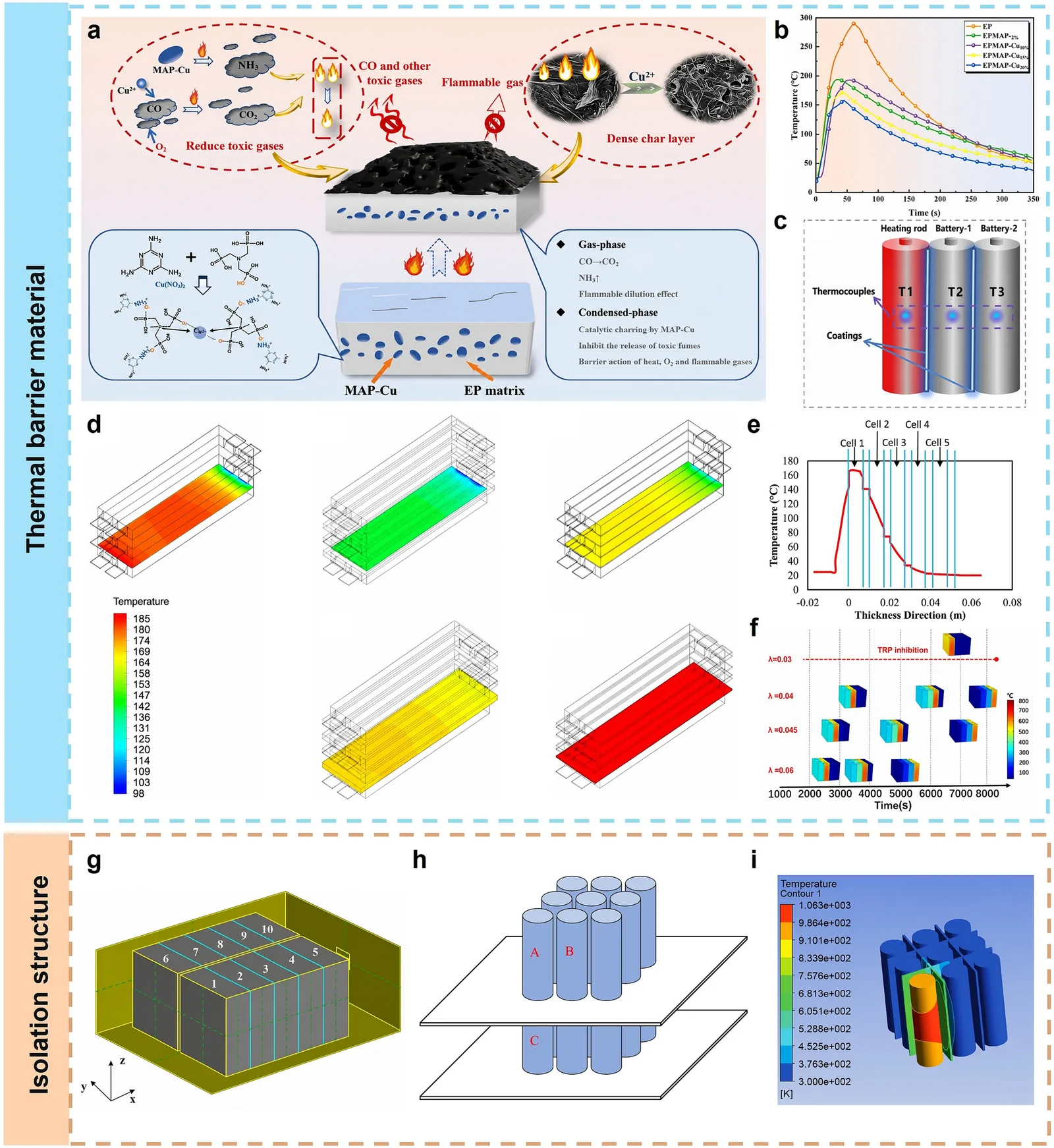
**a** Flame-retardant mechanism of EP/MAP-Cu. **b** Ablation test and temperature change curves of steel plate back. **c** Diagram of the experimental device for battery thermal runaway. **d** Temperature profiles at different interfaces and locations. **e** Temperature distribution along the center line in battery module for Case-A1. **f** 3D distribution temperature of thermal runaway propagation sequence under different thermal conductivity thermal barriers. **g** Physical model of battery pack. **h** Schematic diagram of battery pack arrangement. **i** Simulation temperature field of each monitoring point. Elsevier reproduced with permission from **a**–**c** Ref. [285], Elsevier; **d**, **e** Ref. [9], Elsevier; **f** Ref. [284], © 2023 by the authors; **g** Ref. [286], Springer; **h** Ref. [287], Taylor & Francis; **i** Ref. [288]

At the module and system levels, the application of thermal insulation materials is key to blocking thermal runaway propagation. Metal heat sinks have been widely studied due to their high thermal conductivity and heat capacity. Li et al. [282] found that aluminum plates, as passive heat sinks, have a blocking effect on thermal runaway propagation. This is because aluminum plates absorb and uniformly distribute the energy released during thermal runaway (Fig. 26d). Additionally, the increase in thermal contact resistance due to cell deformation actually helps delay heat transfer and reduce the temperature rise of adjacent cells (Fig. 26e). Therefore, moderately introducing controllable thermal resistance layers is an effective approach for system-level thermal safety design.

Ye et al. [283] added a 1.0 mm thick aerogel layer between the module end plate and the cells, effectively blocking heat conduction on the end plate side. Meanwhile, arranging mica plates on the inner side of the battery pack cover dispersed the thermal impact of flames and high-temperature gases, preventing casing burn-through. These insulation strategies successfully limited the temperature rise of adjacent modules to below 200 °C in experiments, preventing thermal propagation. Liu et al. [284] systematically investigated the influence of thermophysical properties of aerogel insulation layers on TRP suppression through parametric simulations, finding that thermal conductivity is the most critical factor for TRP suppression (Fig. 26f). Anisotropic insulation layers can significantly improve the critical threshold. Increasing specific heat capacity delays propagation time and reduces peak heat flux, while density has a minor effect. This provides quantitative guidance for material selection and structural design of insulation layers.

Fire-retardant insulation coatings are also an effective strategy for blocking thermal runaway propagation. Zhang et al. [285] developed an intelligent self-responsive insulation coating (EP/MAP-Cu) based on epoxy resin and Cu^2^^+^-modified intumescent flame retardant. The flame-retardant mechanism of this coating originates from the strengthening effect of Cu^2+^ on the char layer and the gas-phase/condensed-phase synergistic flame-retardant effect of P/N elements (Fig. 26a). At high temperatures, it forms a continuous, porous, high-strength intumescent char layer, significantly delaying heat transfer. When coated on the surface of LFP batteries, it delayed thermal runaway occurrence time by 51% and reduced surface temperature rise by 36% (Fig. 26b). After coating a 3-mm-thick layer on the inner side of the module casing, the peak casing temperature was reduced to below 93 °C (Fig. 26c), demonstrating excellent insulation and fire protection performance. However, this coating still has the issue of thickness affecting energy density, requiring future development toward thinner, integrated designs.

To compare the effectiveness of different insulation materials in suppressing thermal runaway propagation, Zou et al. [289] systematically studied three 1-mm-thick insulation materials: pre-oxidized fiber aerogel AG-ST-POF, polymer-coated aerogel PC-AG-ST-POF, and silicone (SI). None of the three materials completely prevented thermal runaway propagation, but they significantly delayed the propagation process, with the delay effect ranked as AG-ST-POF > SI > PC-AG-ST-POF. Further analysis indicated that maximum service temperature and thermal diffusivity α are key parameters for screening insulation materials. Future research should optimize thickness design to achieve efficient thermal propagation blocking while maintaining energy density.

##### Structural Isolation

Studies have shown that under constant contact area, the thermal runaway propagation speed of a battery is proportional to its thickness [290], and non-solid heat transfer factors such as high-temperature gases and jet flames produce a significant acceleration effect, which sharply increases with increasing battery thickness. Bai et al. [286] studied large-capacity prismatic LFP battery modules and found that a 2-mm-thick insulation material achieved the best balance between delaying thermal runaway propagation time and module energy density. In complex modules, setting the spacing between adjacent battery rows to 10 mm effectively prevented rapid propagation from one side to the other (Fig. 26g).

As the most fundamental means of thermal propagation blocking, optimization of battery spacing is critical. Wang et al. [287] systematically quantified the critical spacing for preventing thermal runaway propagation in 18,650 battery modules in open and enclosed environments through experiments. In open environments, horizontal spacing greater than 2 mm and vertical spacing greater than 8 mm effectively blocked propagation (Fig. 26h). In enclosed environments, the required horizontal spacing increased to more than 4 mm. This indicates that the vertical direction is at higher risk due to direct impact from high-temperature ejecta, and the enclosed environment significantly exacerbates propagation due to heat accumulation. However, this study was based on specific batteries at full charge; future research should conduct multi-factor coupling studies on more systems.

Qin et al. [288] confirmed through experiments and simulations that introducing glass fiber separators with specific gaps in 18,650 battery packs significantly blocked heat transfer to adjacent cells (Fig. 26i). Simulation data analysis showed that increasing separator thickness contributed approximately 2.74 times more to reducing battery surface temperature than increasing cell spacing. Large-gap structures between modules effectively prevent thermal runaway propagation to adjacent modules, representing an important design concept for system-level structural isolation. Blet et al. [291] used aluminum alloy spacers with precise gaps in a module structure. Under various severe trigger scenarios, including corners, edges, and centers, thermal runaway propagation was completely blocked, validating the effectiveness of this strategy.

In addition to traditional fire-retardant coatings and rigid separators, innovative gap materials targeting specific failure modes are continuously emerging. For the most destructive sidewall rupture of batteries, studies have shown that filling functional foam materials between cells effectively cushions physical impact and prevents thermal runaway propagation. Notably, the insulation provided by such foam allows adjacent cells to maintain their open-circuit voltage after an accident, enabling selective recycling and system recovery, greatly enhancing the economy and sustainability of system safety. Meanwhile, under near-realistic conditions without cover plates, low-density foam may exhibit better comprehensive performance, favoring lightweight barrier design [292].

#### Multifunctional Integrated Thermal Management Materials

Against the backdrop of battery systems evolving toward higher safety and integration, single-function thermal management materials are no longer sufficient to cope with complex operating conditions. The construction of multifunctional integrated systems follows a “material-component-system” co-design approach, with the core being to break the boundaries of traditional single-function materials and achieve optimal configuration and dynamic linkage of multiple thermal management mechanisms in space and time, enabling co-design of thermal management and thermal protection.

**Fig. 27:**
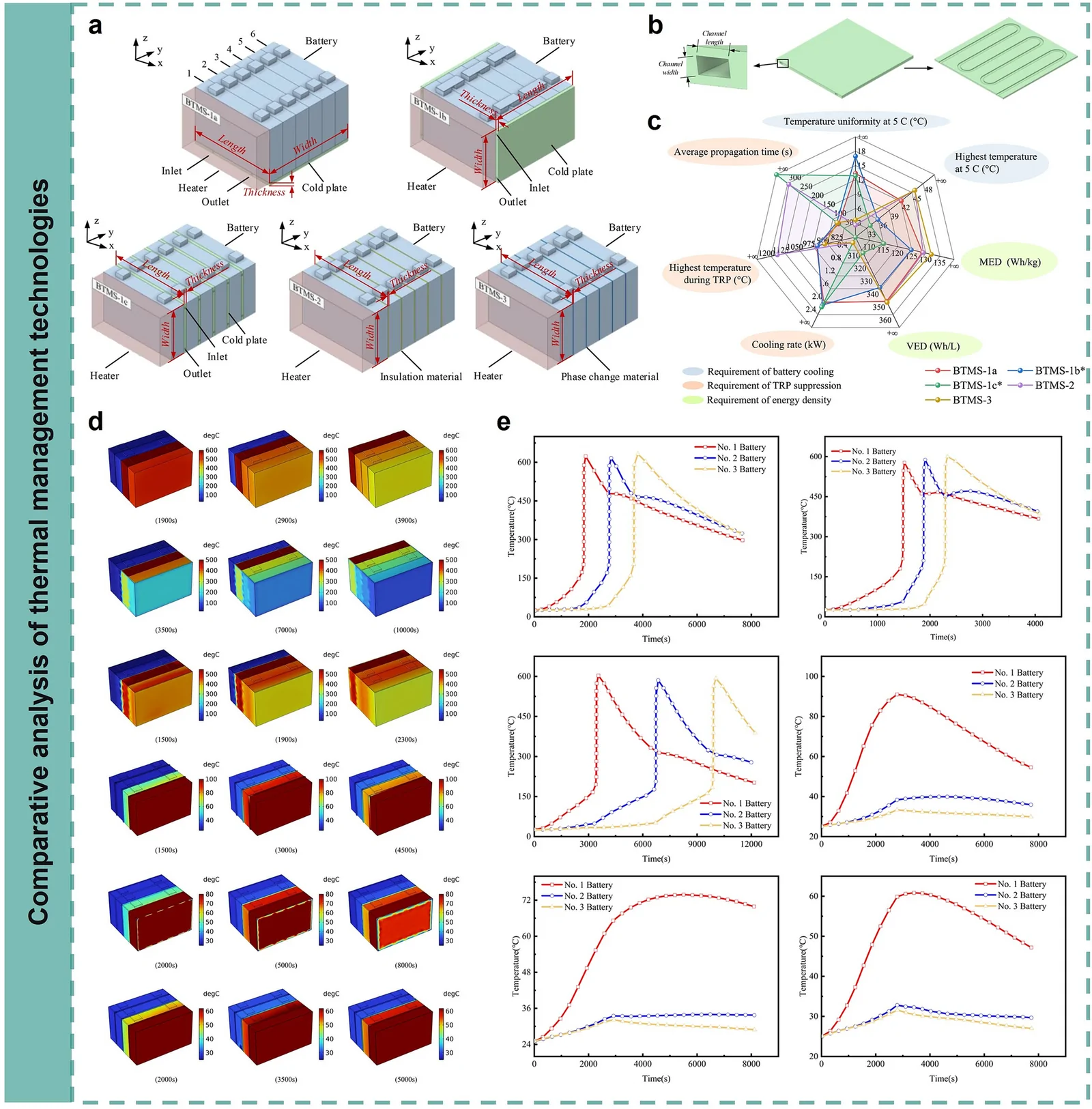
Schematic of different **a** BTMSs and **b** cold plates. **c** Comprehensive comparison of different BTMSs. **d** 3D temperature distribution of each scheme at varying time points and **e** effects of different schemes on TR propagation: No measures; PCM; Aerogel; Liquid cooling; PCM and aerogel; PCM, aerogel and liquid cooling. Elsevier reproduced with permission from **a**–**c** Ref. [293], Elsevier; **d**, **e** Ref. [293]

At the battery system level, efficient suppression of thermal runaway propagation requires synergistic design of heat dissipation and insulation. Zuo et al. [293] proposed a composite thermal management system (BTMS) integrating PCM, aerogel insulation layer, and liquid cooling plate for suppressing thermal runaway propagation in lithium-ion batteries. Numerical simulations comparing six different thermal management methods (Fig. 27d) showed that the integrated scheme reduced the maximum temperature of the thermal runaway cell from 620 to 60 °C (Fig. 27e), significantly outperforming single-material or single-cooling methods, intuitively demonstrating the superiority of multi-mechanism synergy. Further systematic comparison of the effectiveness of five different battery thermal management technologies, including three liquid cooling designs (bottom cooling plate, side cooling plate, and inter-cell cooling plate), insulation materials, and PCM, in suppressing thermal runaway propagation (Fig. 27a-c), revealed that the inter-cell cooling plate design completely suppressed thermal runaway at high flow rates, suitable for large electric vehicles and energy storage systems [293]. PCM excelled in reducing thermal runaway peak temperature and improving energy density, suitable for space-constrained small systems. This comparison provides a basis for selecting options for different scenarios.

**Fig. 28:**
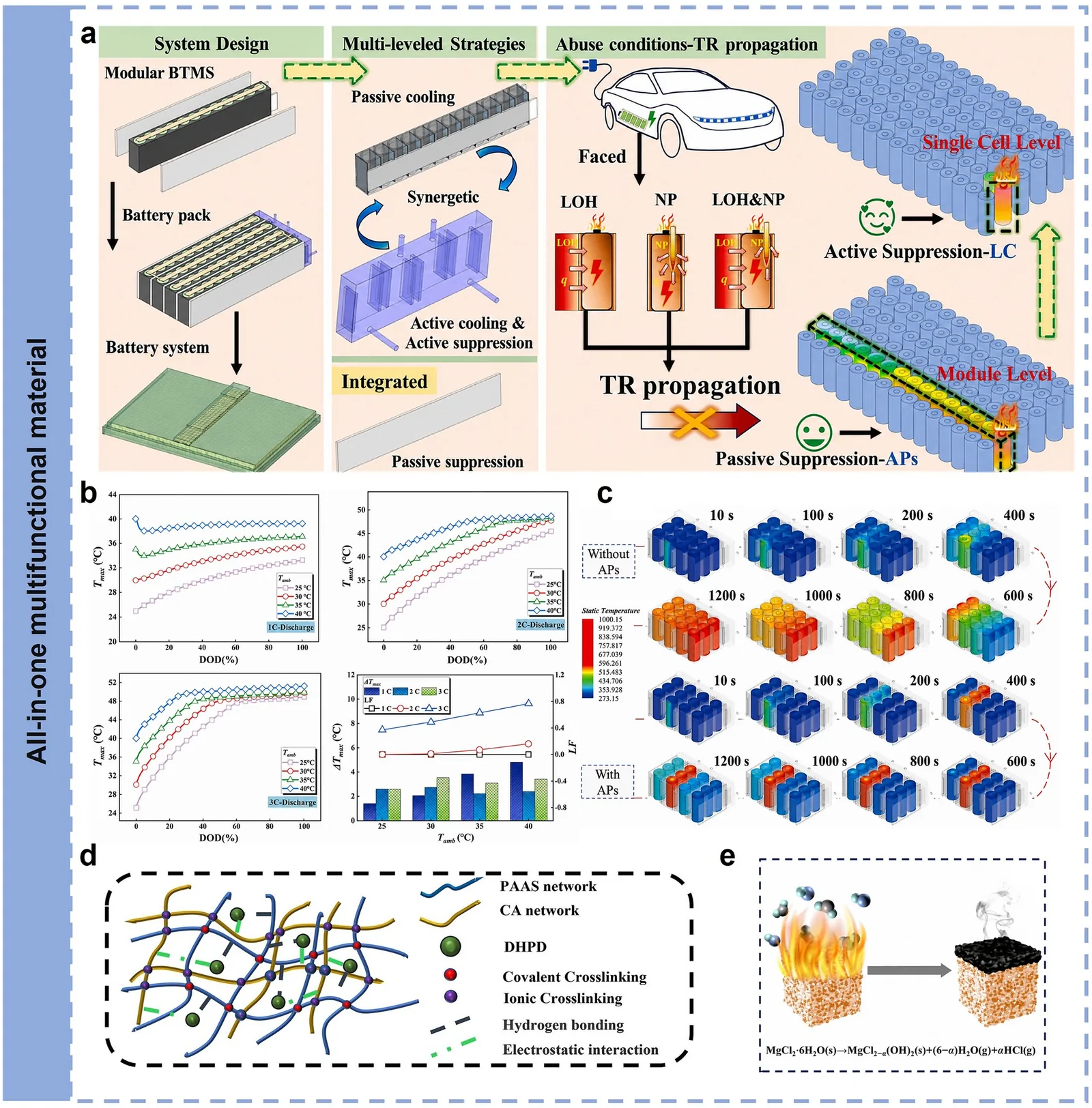
**a** Schematic diagram of the integrated system for TR prevention and TR propagation suppression. **b** Thermal performance of the battery pack with Aps. **c** Temperature contours of the battery packs with and without APs under Mode II triggered by LOH. **d** Schematic diagram of a double-gel network. **e** Flame-retardant mechanism. Elsevier reproduced with permission from **a**–**c** Ref. [294], Elsevier; **d** Ref. [295], American Chemical Society; **e** Ref. [296]

Traditional composite phase-change materials face a fundamental contradiction in thermal management: high thermal conductivity is needed for rapid heat dissipation under normal operating conditions, while low thermal conductivity is required to block thermal propagation during thermal runaway. To address this challenge, researchers have developed novel materials with intelligent response characteristics. Li et al. [297], inspired by the thermal oxidation characteristics of MXene nanosheets, developed a thermally triggered composite phase-change material with tunable thermal conductivity, MF@MXene/PA. By adsorbing paraffin (PA) into an MXene-reinforced melamine–formaldehyde resin porous framework, the synergistic effects of PA phase change heat absorption and MXene/MF high-temperature carbonization achieved dual functions: efficient heat dissipation under normal conditions and thermal blocking during thermal runaway. Another novel dual-function flame-retardant composite phase-change material (CPCM) [296] introduced magnesium chloride hexahydrate (MCH) as a substitute for traditional flame retardants. While maintaining PA’s normal operating thermal management performance, the irreversible dehydration decomposition reaction of MCH in the 100–300 °C range provides an endothermic capacity as high as 1653 J g^−1^, giving the composite material a total heat absorption of 800 J g^−1^ in the thermal runaway temperature range. The flame-retardant mechanism encompasses both gas-phase dilution and condensed-phase char formation (Fig. 28e). This material not only controls the operating temperature of battery modules at relatively low levels but also absorbs a large amount of heat during simulated thermal runaway, effectively delaying its trigger and propagation. However, this material faces challenges such as irreversible consumption of MCH and corrosive decomposition products.

In recent years, multifunctional integrated thermal management materials with both potting processability and high heat storage density have become research hotspots. Li et al. [295] developed a hydrated salt gel based on disodium hydrogen phosphate dodecahydrate for filling battery gaps (Fig. 28d). This material possesses both phase change enthalpy and thermal decomposition enthalpy to address the high-temperature sensitivity of lithium-ion batteries and the poor temperature control performance of current battery potting compounds. It can be used to encapsulate battery packs, providing temperature protection and thermal runaway protection through potting and curing.

Xie et al. [294] proposed and validated a multi-level, multifunctional intelligent thermal management integrated system (Fig. 28a), achieving an integrated safety solution from material composite to system synergy through deep integration of composite phase change materials, super-insulating aerogels, dynamic liquid cooling, and modular architecture. High-thermal-conductivity composite phase change material was used as the encapsulation matrix for battery modules, efficiently absorbing and uniformly distributing battery heat during normal operation to maintain temperature equilibrium. Aerogel panels were inserted between adjacent battery modules to form physical firewalls with ultra-low thermal conductivity, effectively blocking heat transfer to adjacent modules during thermal runaway, confining the thermal runaway within a single module. An integrated intelligent adjustable flow liquid cooling system dynamically adjusts cooling flow rates based on system thermal conditions, enabling seamless switching from low-power daily heat dissipation to high-power emergency cooling (Fig. 28b, c).

In summary, efficient thermal management and propagation barrier technologies are undergoing a profound evolution from single-function materials toward multifunctional integration, from passive protection toward active regulation, and from empirical design toward quantitative prediction. At the mechanistic level, the establishment of theoretical criteria such as TRPN provides a scientific basis for system design. In thermal management technology, the synergistic optimization of active liquid cooling, immersion cooling, and passive phase-change materials and heat pipes significantly improves heat dissipation efficiency. At the barrier level, aerogels, fire-retardant coatings, and structural isolation designs effectively delay thermal runaway propagation. At the integration level, multifunctional integrated materials achieve synergistic protection of “heat dissipation-insulation-flame retardancy.” Future efforts should further deepen the understanding of thermal propagation mechanisms, develop high-precision simulation tools, optimize material parameters and structural designs, and construct multi-level thermal safety protection systems, providing solid support for the development of next-generation battery systems with high energy density and high safety.

This section systematically summarizes the relevant technical approaches, core mechanisms, and safety effects from three dimensions: enhanced heat dissipation design, thermal propagation barrier technology, and multifunctional integrated thermal management materials. Table 6 provides a categorized summary of the above content.

**Table 6:** Summary of efficient thermal management and propagation barrier technologies

Category	Subcategory	Key technology	Core mechanism	Reference
Enhanced heat dissipation design	Theoretical prediction	Uncertainty quantification framework for thermal runaway propagation	Identifies SOC, PCM thermal conductivity, and cell spacing as key factors; reveals nonlinear mutation threshold in system	[263]
		Thermal runaway propagation number (TRPN) criterion	Integrates heat generation kinetics, thermal conductivity, heat dissipation intensity, etc., provides theoretical boundary for propagation	[264]
		Dimensionless theoretical framework	Abstracts complex propagation into dimensionless parameter control; outputs average mass consumption rate, relates to fire hazard	[265]
	Active thermal management	Liquid cooling, air cooling	Consumes external energy for precise temperature control	[266, 267]
		Immersion cooling, dynamic immersion cooling	Uniform heat dissipation via medium flow, effectively avoids heat accumulation and propagation	[268]
		Micro-volume liquid immersion cooling	Utilizes golden window after pressure relief; minimal coolant can suppress self-heating process	[272]
		Spray cooling, water mist cooling	Dual mechanism of evaporative heat absorption and oxygen isolation	[269, 273]
	Passive thermal management	Phase-change materials (PCMs)	Absorbs/releases large amounts of latent heat during phase transition, reduces peak temperature, slows temperature rise	[274]
		Heat pipes	Utilizes phase change circulation of internal working fluid for rapid heat transfer	[278]
		Thermal cloak structure	Inner PCM absorbs heat, outer high-thermal-conductivity metal establishes rapid radial heat dissipation	[275]
		Structured expanded surfaces	Increases effective heat dissipation area, enhances convective heat transfer, improves mechanical impact resistance	[276]
		Composite phase change materials	Integrates high thermal conductivity, phase change heat storage, and structural support	[279]
		Geometric design optimization of PCM	1 mm thick PCM effectively reduces battery temperature under high-rate discharge	[236]
		Heat pipe + PCM coupled system	Heat pipe efficiently dissipates heat, PCM absorbs and stores heat	[237]
Thermal propagation barrier technology	Thermal insulation materials	Aluminum plates	Absorbs and distributes thermal runaway energy; increased thermal contact resistance due to cell deformation	[282]
		Aerogel layers, mica plates	Blocks heat conduction at end plates; disperses thermal impact of flames and high-temperature gases	[283]
		Fire-retardant insulation coatings (EP/MAP-Cu)	Forms continuous, porous, high-strength expanded carbon layer at high temperatures	[285]
	Structural isolation	Battery spacing optimization	Critical spacing exists; vertical spacing is more critical due to direct impact from high-temperature ejecta	[287]
		Glass fiber separators with specific gaps	Effectively blocks heat transfer to adjacent cells	[288]
		Module-level large-gap structures	Effectively prevents propagation to adjacent modules	[291]
		Functional foam materials	Cushions physical impact, prevents propagation	[292]
Multifunctional integrated thermal management materials	System-level integration	Integrated BTMS with PCM, aerogel insulation layer, and liquid cooling plate	Combines multiple mechanisms (heat absorption, insulation, active cooling)	[293]
		Integrated system with composite PCM, aerogel, dynamic liquid cooling, and modular architecture	Composite PCM for heat absorption and distribution; aerogel as physical firewall; dynamic liquid cooling for adaptive heat dissipation	[294]
	Smart responsive materials	Thermally switchable thermal conductivity CPCM (MF@MXene/PA)	High thermal conductivity for normal operation; high-temperature carbon formation for thermal blocking	[297]
		Dual-functional flame-retardant CPCM	Irreversible dehydration decomposition provides high heat absorption	[296]
	Processable high-density materials	Hydrated salt gels (Na_2_HPO_4_·12H_2_O)	Combines phase-change enthalpy and thermal decomposition enthalpy	[295]

### System-Level Safety Design and Failure Response

The safety of energy storage systems depends not only on individual cells but is also profoundly influenced by the system operating environment, electrical connection methods, and topological structures. This section systematically elaborates on system-level safety design and failure response strategies from three dimensions: BMS safety logic, mechanical design for pressure relief, fire and explosion prevention, and electrical connections and topological structures.

#### BMS Safety Logic

The BMS is a crucial component of LIB packs, ensuring safe and efficient battery operation by monitoring and controlling the charging and discharging processes [298]. The main functions of the BMS include monitoring battery voltage, current, and temperature, balancing the charge of each cell in the pack, and providing protective control when anomalies occur [299]. By continuously collecting key battery parameters in real time, the BMS can accurately estimate SOC and SOH of the battery, adjusting the charging and discharging processes accordingly to optimize performance and extend battery life [300]. As the brain of the battery pack, optimization of BMS safety logic is key to achieving the transition from passive response to active prevention. This section reviews research progress in BMS safety logic optimization from three dimensions: dynamic thresholding, hierarchical strategies, and coordinated response.

Dynamic thresholding refers to adaptive adjustment of warning thresholds based on environmental perception. Traditional BMS uses fixed temperature thresholds, which are difficult to adapt to complex operating conditions. Tian et al. [46] found through COMSOL simulation that when the ambient temperature rises from -10 to 50 °C, the thermal runaway trigger time in energy storage containers can advance by 33 min. This finding reveals that the BMS needs to dynamically adjust warning thresholds based on ambient temperature. Establishing an ambient temperature-trigger time mapping library enables adaptive threshold adjustment.

Hierarchical strategies refer to graded response based on thermal runaway propagation types. Chen et al. [47] proposed for the first time a graded response strategy based on thermal runaway propagation types. The study found that thermal runaway propagation can be divided into two types. The T_onset_ type refers to adjacent cells being directly heated above the trigger temperature without an intervention window, requiring high-rate discharge or full-flooding fire suppression. The T_initial_ type refers to adjacent cells entering the self-heating accumulation stage after being delayed by insulation materials, presenting an intervention window during which micro-current electrochemical regulation can be applied to suppress heat generation at the source. Specifically, a weak discharge current matching the electron transfer rate of harmful interfacial reactions is applied to the at-risk cell. This current preempts electrons at the anode-electrolyte interface, inhibiting key exothermic pathways at the chemical reaction source, thereby fundamentally blocking thermal runaway propagation with almost no energy consumption. This study provides a novel graded response logic for BMS. Similarly, Theiler et al. [301] proposed and validated a thermal propagation suppression strategy based on active SOC management. By constructing a small module consisting of three pouch cells, the thermal propagation behavior under uniform and non-uniform SOC distributions was systematically compared. The results showed that selectively reducing the SOC of key cells on the propagation path fundamentally weakens the driving source of thermal runaway, significantly reducing the maximum temperature and ejection intensity of the runaway cell. This is because lower SOC reduces the amount of reactive lithium in the anode and the oxygen release capability of the cathode, thereby reducing the overall reaction enthalpy and gas generation rate, making the thermal runaway process more moderate. This study further confirms the feasibility of actively regulating SOC through BMS to intervene in thermal runaway propagation, providing a new technical pathway for graded response strategies. However, the Joule heat generated by the emergency discharge process itself may pose a new thermal risk, which must be balanced in BMS decision-making algorithms.

Coordinated response refers to multi-subsystem coordinated control. Based on thermal propagation models and real-time monitoring data, the BMS can coordinate thermal management systems, electrical protection systems, fire suppression systems, and mechanical protection systems. Lu et al. [173] showed that when one cell in a module experiences thermal runaway, immediately applying ultrahigh-rate emergency discharge to adjacent cells can sharply reduce their SOC, transforming violent jet flames into smoke and effectively prolonging thermal runaway propagation time. This provides key data support for BMS decision-making algorithms under disaster conditions. For large-format, high-capacity modules, the tight arrangement and high energy concentration make thermal runaway propagation risks more severe [302]. Active liquid immersion systems, as an emergency response measure, have attracted attention. Meelapchotipong et al. [303] developed a seawater immersion method triggered by voltage drop, which rapidly cools and terminates reactions in the early stage of thermal runaway. The implementation of such active-response strategies also relies on precise triggering and coordinated control by the BMS.

Furthermore, the application of machine learning and deep learning algorithms in BMS has become an important research direction, enabling real-time analysis of battery data and enhanced system intelligence [304]. In recent years, intelligent algorithms such as incremental capacity analysis (ICA), adaptive neuro-fuzzy inference systems (ANFIS), genetic algorithms (GA), and deep learning have been widely used to improve BMS state estimation accuracy and decision-making reliability and are gradually being integrated with thermal management systems to construct system-level safety architectures with multi-objective optimization and robust control under uncertainty. Therefore, the intelligentization and integration of BMS are key technological supports for achieving efficient, long-life, and high-safety operation of battery systems. The safety design of battery systems must consider performance evolution throughout the full life cycle. Cumulative thermal damage is a key factor leading to degradation of battery safety boundaries [25]**.** Batteries that have undergone thermal aging have reduced mechanical abuse safety thresholds, potentially earlier thermal runaway critical temperatures, and significantly shortened incubation periods. However, the capacity fade of these batteries may not be obvious, posing a hidden safety risk. Therefore, advanced BMS must not only monitor real-time status but also integrate aging models and safety boundary prediction functions to dynamically adjust safety control strategies.

In summary, the battery management system is the core for ensuring safe and efficient operation of LIB systems and serves as the decision-making brain for implementing precise thermal management and active safety strategies. As electric vehicles and large-scale energy storage systems evolve toward higher energy densities, faster charging speeds, and more complex operating environments, higher requirements are placed on the sensing accuracy, decision-making intelligence, and robustness of BMS. Therefore, promoting continuous innovation and breakthroughs in BMS technologies in areas such as multi-state fusion estimation, intelligent fault diagnosis, adaptive thermal management, and system-level coordinated control is the future development direction.

#### Mechanical Design for Pressure Relief, Fire and Explosion Prevention

**Fig. 29:**
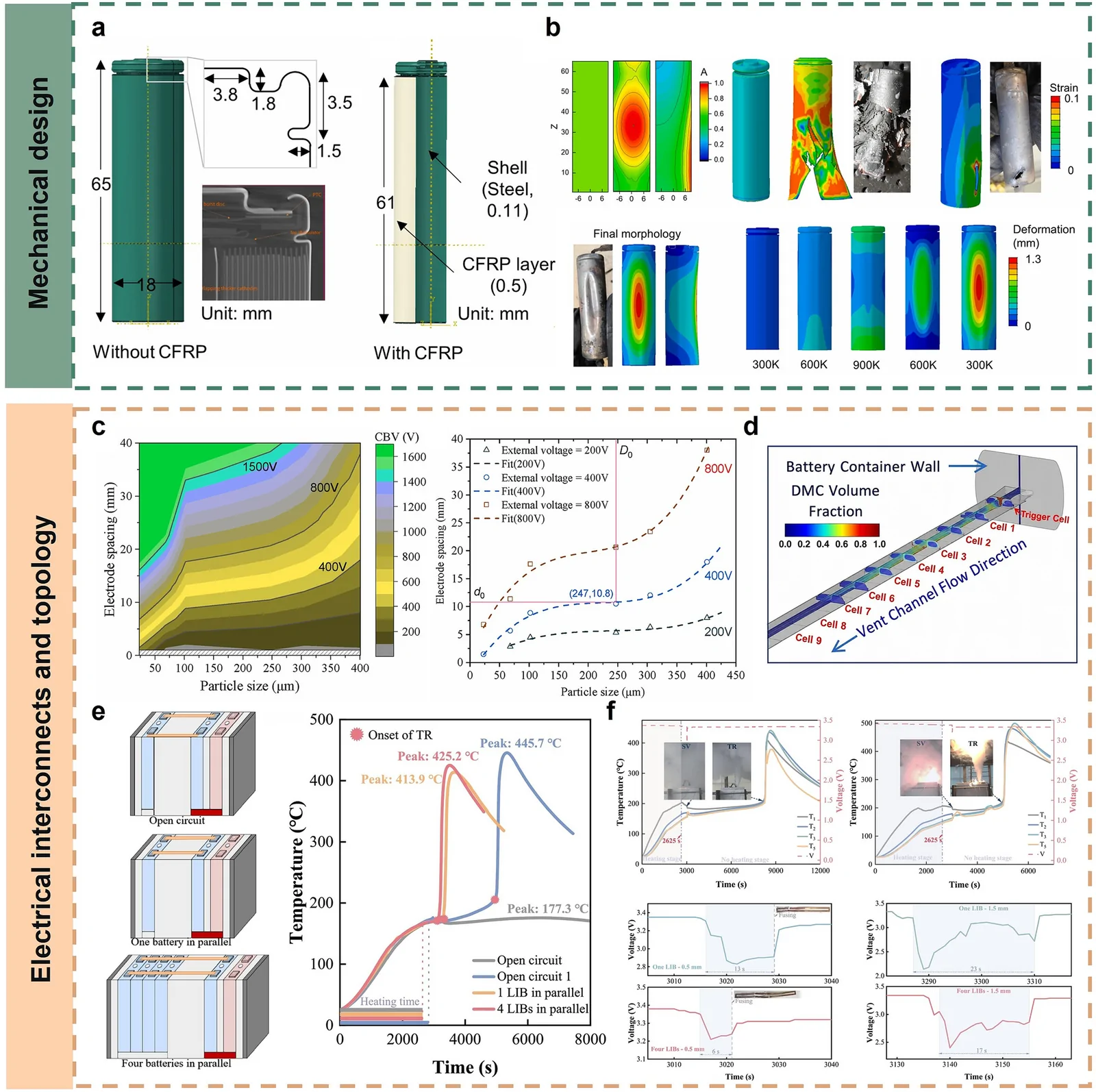
**a** Model geometries of the cell shell. **b** Fracture behavior without CFRP layer and fracture/deformation with CFRP layer. **c** Critical voltage map as a function of electrode spacing and particle size, with a parametric evaluation and design method to prevent particle-induced arc. **d** CFD simulation results of DMC venting from a trigger cell. **e** Configurations of three battery modules and the temperature variations of the heated batteries during the preliminary experiment. **f** Temperature and voltage variations of the heated battery under parallel configurations with one and four LIBs, and voltage variations with copper connectors of 0.5 mm and 1.5 mm thickness. Elsevier reproduced with permission from **a**, **b** Ref. [49], Elsevier; **c** Ref. [52]. The Electrochemical Society; **d** Ref. [10], Elsevier; **e**, **f** Ref. [50]

The mechanical response of the battery casing during thermal runaway directly affects pressure relief effectiveness and thermal propagation risk. Traditional LIB safety design relies mainly on pressure relief valves that open when internal pressure becomes too high, releasing flammable gases to reduce explosion risk. However, after the pressure relief valve opens, high-temperature flammable gases come into contact with air, easily causing fire or even explosion. To address this challenge, Hofmann et al. [305] proposed an active explosion prevention strategy based on negative pressure. By applying negative pressure inside the battery during thermal runaway, flammable gases are forcibly extracted, and solvent evaporation absorbs heat, preventing battery explosion and fire. However, this strategy still faces engineering feasibility challenges, including the impracticality of pre-embedded pipelines inside the battery and irreversible damage caused by electrolyte removal. Jia et al. [49] studied the deformation and rupture behavior of 18,650 cylindrical battery casings under thermal runaway through experiments and simulations (Fig. 29b), finding that the rupture mode is highly dependent on internal temperature distribution. Uniform temperature leads to end cap rupture, while lateral heat concentration causes sidewall rupture. The study proposed wrapping the battery casing with a carbon-fiber-reinforced polymer (CFRP) layer (Fig. 29a), which effectively prevents sidewall rupture but may cause axial indentation due to thermal expansion mismatch.

In terms of pressure relief valve and explosion-proof design, Finegan et al. [48] used ultrahigh-speed synchrotron X-ray imaging to observe two rupture mechanisms in 18,650 batteries during thermal runaway: electrode stack collapse blocking the venting path and insufficient venting capacity leading to pressure accumulation, confirming that the mismatch between gas generation and venting capacity is the root cause of rupture. Based on this mechanism, dual venting holes effectively achieved bidirectional pressure relief, preventing structural rupture. The lower explosion limit (LEL) of gases is also a key parameter; pressure relief valves should ensure complete venting before gases approach the LEL, with directional flow-guiding and flame-retardant functions. Jet velocity and jet length are important risk indicators; module and system layouts should ensure safe distances greater than the maximum jet range, or physical barriers and thermal shields should be installed.

When thermal runaway has already occurred, traditional external thermal management and passive protection measure often become ineffective. At this point, built-in active fire suppression systems become the engineering line of defense against disaster escalation. Kang et al. [306] demonstrated the strong effectiveness of directly injecting water into the battery box for extinguishing thermal runaway fires. Water effectively prevented chain explosions and blocked the release of harmful gases from the battery.

#### Thermal Safety Implications of Electrical Connections and Topological Structures

The electrical connection methods and topological structures within battery systems directly determine current paths and heat distribution under fault conditions, thereby affecting the trigger and propagation behavior of thermal runaway. Studies on nickel-rich battery systems have shown that under the same material system, batteries with higher energy density exhibit more severe thermal runaway behavior, including higher peak pressure, greater mass loss, and a wider flammable range of gases [87]. Therefore, when integrating systems, the electrical topology and energy distribution strategies must fully consider the positive correlation between the intrinsic energy density of individual cells and the severity of thermal runaway. Furthermore, the electrical connection method directly affects the propagation behavior and speed of thermal runaway. Parallel electrical topologies introduce electrothermal coupling risks [51]. By providing a low-resistance path for electrical energy transfer, they significantly exacerbate thermal runaway propagation risks [50], resulting in propagation times shorter than those of series or open-circuit structures (Fig. 29e). The root cause is that the voltage collapse of the thermal runaway cell induces adjacent cells to input large currents through the connectors, and the generated Joule heat rapidly triggers thermal runaway in adjacent cells. The thickness and material of the connectors are also crucial; thinner copper connectors can fuse under excessive current, interrupting hazardous electrical energy transfer, while thicker connectors may sustain conductivity, exacerbating the disaster (Fig. 29f). Future electrical topology design must incorporate controllability and interruptibility of electrochemical propagation as important safety design metrics.

During thermal runaway, in addition to traditional risks such as heat, gas, and fire, electrical failure may also be induced, particularly arc faults triggered by ejected particles, further exacerbating thermal runaway propagation and fire risk. Li et al. [52] revealed and validated the electrical failure mechanism of particle-induced arc faults in LIB systems for the first time. Ejected particles act as catalytic media in the electrode gap, reducing the critical breakdown voltage to 0.9–2.3% of that in uniform air. The critical breakdown voltage is positively quadratic with electrode spacing and significantly negatively correlated with particle size. A critical voltage map based on electrode spacing and particle size was constructed (Fig. 29c), providing a quantitative tool for the engineering design and safety assessment of electrical clearances in battery systems.

The pre-venting and subsequent combustion of electrolyte solvents pose new safety challenges to the topological structures and electrical layouts of battery modules. Srinivasan et al. [307] found through in situ FTIR spectroscopy and CFD simulations that trigger cells release large amounts of flammable carbonate solvents through pressure relief valves before reaching thermal runaway. These high-temperature gases rapidly cool in the venting channels and condense on the surfaces of adjacent cells (Fig. 29d). When the trigger cell enters thermal runaway, high-temperature ejecta ignite the condensed solvents on the cell surfaces, and the heat released from combustion is sufficient to trigger thermal runaway in adjacent cells. Therefore, the venting channels of battery modules should avoid direct orientation toward adjacent cells, and condensate capture devices or inert gas purging could be used to block solvent migration and adhesion between cells. Ke et al. [308] developed a serpentine-channel liquid-cooled thermal management system. The lateral diffusion of high-temperature electrolyte through positive electrode metal connectors was identified as the main propagation mechanism, with propagation speeds much higher than those of heat conduction and radiation. Blocking electrolyte jetting with insulating sleeves prevented propagation even without coolant flow. Therefore, electrical connection designs should avoid covering the positive electrode of cells, and the liquid cooling system must achieve a critical flow rate for effective temperature control.

It is worth noting that traditional modules composed of a single chemical system have inherent limitations in thermal safety. As sodium-ion batteries (SIBs) [5, 309–311] have gained widespread attention due to their abundant resources, low cost, and good thermal stability, Zhu et al. [312] constructed a hybrid battery pack combining sodium-ion and ternary lithium (NCM) batteries. Through multi-chemical system synergy [313–318] and spatial layout optimization, active management of thermal runaway propagation was achieved. Sodium-ion batteries, with their later thermal runaway time and lower peak temperature, are excellent thermal stability units in hybrid modules. When strategically placed near heat sources, SIBs effectively absorb heat and delay propagation; placing NCM cells at a distance balances energy density. This “intelligent thermal buffering” effect achieved through spatial layout of multiple chemical systems reveals the profound impact of battery topological structures on system thermal safety, offering new ideas for the design of next-generation hybrid battery systems with high safety and high energy density.

System safety design must consider the full-chain consequences of thermal runaway, including secondary disasters after thermal runaway occurs. Among these, high-temperature, high-velocity jet flows are among the most direct and dangerous threats. Therefore, the design of battery packs and energy storage cabinets must not only satisfy pressure relief requirements but also incorporate directional flow guidance, kinetic energy dissipation, and thermal isolation for jet flows. Explosion-proof ventilation channels with sufficient flow area and impact resistance should be designed based on measured jet velocities. The inclination uncertainty of jet flows should be used to plan battery spacing and the installation angles of fire-resistant barriers. When thermal runaway protection fails and a fire occurs, accurate accident investigation [319] and root-cause analysis are crucial for preventing recurrence of similar events. Traditional investigations rely heavily on experience, and firefighting operations often destroy key evidence. Recent research has begun to establish scientific fire trace analysis methods [7]. Systematic analysis of LFP battery thermal runaway residues has been conducted to construct a “thermal runaway trace-trigger cause” mapping database, providing a unique research dimension for inferring failure mechanisms from disaster outcomes and thereby improving safety design.

Additionally, the release of toxic and flammable gases and submicron particles must be managed. The large amounts of toxic smoke and dust generated during thermal runaway [74] are hazards often overlooked in traditional safety designs. The fine particle size and large specific surface area of smoke and dust make them prone to forming long-suspended aerosols, with significant cytotoxicity to the human respiratory and nervous systems. Therefore, in system-level safety design, in addition to traditional hazards such as flames, gases, and explosions, smoke and dust generation and control must be incorporated into the protection system, including the development of smoke and dust suppression materials and venting and filtration designs oriented toward emission control. Furthermore, instantaneous gas release can generate impact pressure, and system structures should be designed with impact resistance and pressure relief devices.

In summary, system-level safety design must comprehensively consider the coupling effects of multiple physical fields. BMS safety logic is evolving from monitoring–alarming toward monitoring-decision-active intervention, achieving precise protection through dynamic thresholding, hierarchical strategies, and coordinated response. Pressure relief and explosion-proof design should be based on casing rupture mechanisms, optimizing pressure relief valve layout and venting capacity. Electrical connections and topological structures directly affect the propagation path and speed of thermal runaway. Future system-level safety design should evolve toward the combination of active guidance and source suppression, constructing a multi-level safety protection system.

## Summary and Future Perspectives

### Summary

Safety concerns are always the most critical aspect during the use of LIBs. As one of the key causes of accidents, thermal runaway has drawn significant attention. To advance the development of related research, this review presents an in-depth investigation into thermal runaway. Unlike existing reviews that focus on a single dimension, the framework of this review integrates fundamental mechanisms, real-time monitoring, and protective strategies into a complete chain. Rather than merely cataloging materials, the systematically traced for the trajectory revealing the underlying trend toward increasingly smart thermal safety systems. Furthermore, thermal management, design, and protection technologies at both the cell and system levels are comprehensively summarized and analyzed in this review. It is hoped that offering a panoramic theoretical perspective for battery thermal safety research. The main conclusions are as follows:

Regarding TR mechanisms, this paper dissects the triggering conditions and internal chain reactions. TR is primarily induced by thermal, electrical, and mechanical abuse, which often occur simultaneously in practical scenarios. Their destructive effects are nonlinearly superimposed, significantly elevating TR risks. The chain reaction of TR is a multistage coupled process, initiating with the thermal decomposition of the anode solid-electrolyte interphase (SEI), progressing through reactions between the lithiated anode and electrolyte, accelerated by interface side reactions induced by lithium dendrites, and ultimately detonated by lithium oxidation reactions triggered by cathode oxygen release and anode lithium contact. This sequence is modulated by the battery’s chemical system, SOC, aging degree, and external heat flux, exhibiting complex kinetic evolution.

In terms of modeling and prediction methods, this paper reviews the spectrum of modeling technologies, ranging from reaction kinetics, thermal, and electrical models to multiphysics coupled models. Although high-fidelity models can precisely describe the TR process, their computational complexity limits engineering applications. Therefore, model simplification, order reduction, and integration with data-driven methods represent crucial future directions. Data-driven models, particularly machine learning algorithms, offer a new paradigm for managing the inherent high stochasticity and complexity of TR; however, their reliance on high-quality, large-scale training data remains a primary challenge.

Regarding real-time monitoring and early warning, this review notes the limitations of traditional single-threshold warnings based on voltage and temperature, particularly against hidden risks like cumulative thermal damage. Consequently, multi-dimensional signal fusion is an inevitable trend. The combination of embedded internal sensors and external noninvasive monitoring is building a comprehensive internal-to-external perception network. Intelligent diagnostic algorithms—especially those based on deep learning, graph neural networks, and unsupervised learning—can effectively extract early fault features from massive, sparse, and high-dimensional data, facilitating the leap from post-event alarming to pre-event prediction.

At the cell material safety strategy level, this paper reveals the profound evolution of safety design from traditional passive protection to active-response smart materials. Passive protection enhances intrinsic stability through single-crystal design, doping, coating, and interface engineering, thereby delaying TR initiation at the source. Conversely, active response achieves intelligent functions such as thermal shutdown, overcharge protection, and self-healing via temperature, voltage, and stress-responsive materials. This review further summarizes the developmental trajectory of this field, from irreversible to reversible, from macroscopic coatings to the particle level, from functional modifications to intrinsic material properties, and from single responses to multimodal integration. These two strategies complement and synergize with each other, collectively enhancing the intrinsic safety of cells.

In system integration and engineering protection, efficient thermal management and propagation barrier technologies are evolving from single-function materials to multifunctional integration, and from passive protection to active regulation. The synergistic application of enhanced heat dissipation and thermal propagation barriers is critical for blocking cascading TR propagation. At the system level, the safety logic of the BMS is evolving from “monitoring-alarming” to “monitoring-decision-active intervention.” Through dynamic thresholds, hierarchical strategies, and synergistic responses, the BMS is becoming the decision-making brain for system-level safety protection. Furthermore, electrical connections and topological structures directly affect the path and speed of TR propagation; parallel electrical topologies exacerbate propagation risks due to electrothermal coupling. Controllable electrochemical propagation and fuse designs must be prioritized as critical safety design indicators.

### Future Perspectives

**Fig. 30:**
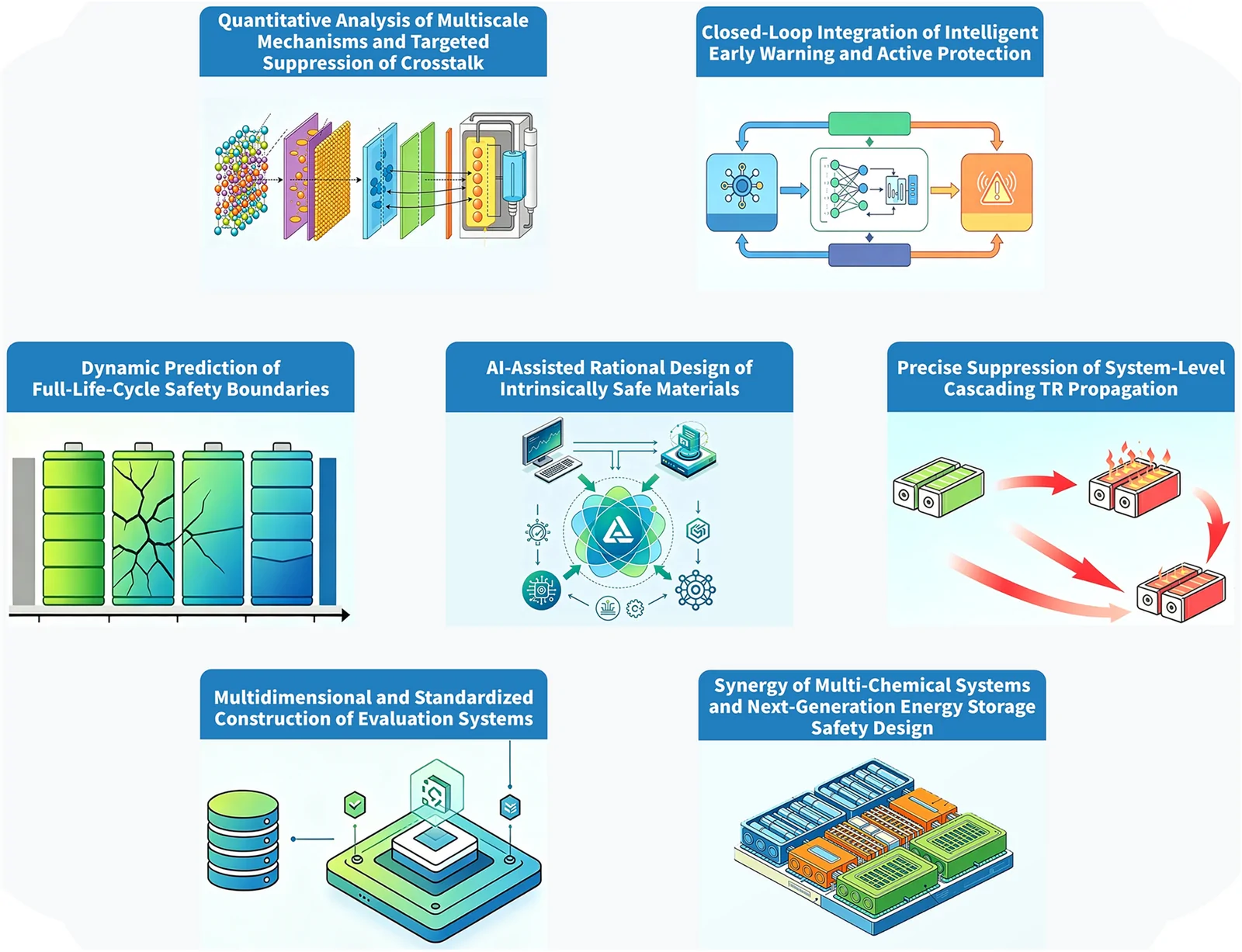
Future perspectives for thermal runaway

Despite the significant progress in the thermal safety of lithium-ion batteries systematically reviewed herein, a series of key scientific questions and engineering challenges remain to be resolved in the face of escalating demands for high energy density and increasingly complex application scenarios. Future research in this field should focus on the following seven core directions (Fig. 30):

1. Quantitative Analysis of Multiscale Mechanisms and Targeted Suppression of Crosstalk: Current understanding of TR chain reactions is mostly limited to qualitative descriptions, lacking quantitative data on kinetic parameters of key reactions and the generation and migration rates of crosstalk species. Future research must develop operando/in situ characterization technologies combined with multiphysics and multiscale simulations to establish quantitative correlation models from the atomic to the system scale. In particular, it is crucial to deeply dissect the physicochemical nature of inter-electrode crosstalk and explore novel pathways to target and suppress detrimental crosstalk reactions via interface engineering, functional separators, or electrolyte additives, thereby severing the accelerated failure network inherent to high-energy batteries at its root.
2. Closed-Loop Integration of Intelligent Early Warning and Active Protection: Existing intelligent early warning systems rely heavily on high-quality multi-dimensional signal inputs, and intervention measures remain relatively limited. Future efforts should further deepen the integration of multi-dimensional signals with machine learning algorithms to achieve ultra-early identification and probabilistic prediction of TR. Simultaneously, there is an urgent need to develop reversible, multimodal active-response mechanisms to construct a “perception-decision-execution” closed-loop intervention system, realizing a leap from passive alarming to active blocking.
3. Dynamic Prediction of Full-Life-Cycle Safety Boundaries: The thermal safety characteristics of batteries are not static but evolve dynamically with the aging process. Existing models have not yet achieved deep coupling between aging mechanisms and TR behaviors. It is imperative to establish “aging-TR” coupled evolution models to systematically evaluate thermal safety degradation laws throughout the full life cycle, accurately identify safety inflection points, and provide scientific safety boundaries for the second-life application of retired batteries.
4. AI-Assisted Rational Design of Intrinsically Safe Materials: Rationally designing intrinsically safe materials at the electronic and atomic structural levels is the ultimate pathway to overcoming TR. Future research should dedicate efforts to developing cathodes with high oxygen release barriers, solid-state electrolytes with high interface stability, and smart nanoporous separators that balance high ionic conductivity with gas-blocking capabilities. The deep intersection of high-throughput computing, the Materials Genome Initiative, and AI technologies will greatly accelerate the targeted screening and optimization of novel intrinsically safe materials.
5. Precise Suppression of System-Level Cascading TR Propagation: TR propagation in large-capacity modules is jointly constrained by material properties, geometric dimensions, thermal boundaries, and electrical topologies. Future work requires the construction of system-level 3D propagation models that fully incorporate separator failure, high-temperature gas-liquid jetting, thermal radiation, convective heat transfer, and electrothermal coupling effects. Based on this, the engineering application of intelligent, adaptive thermal management and barrier technologies should be advanced to achieve targeted isolation of TR and absolute blockage of cascading propagation.
6. Multi-dimensional and Standardized Construction of Evaluation Systems: Given the trade-off between internal TR and external secondary disasters within batteries, safety strategies must be precisely and differentially designed for distinct chemical systems. Future safety evaluation systems must break the boundary of a single “runaway or not” criterion, expanding into a multidimensional, comprehensive assessment framework encompassing heat, gas, fire, toxic smoke, shockwaves, and electrical failure. Meanwhile, the industry urgently needs to collaboratively establish standardized, open-source TR databases and model validation platforms to provide a unified benchmark for fundamental theoretical research and engineering protection standards.
7. Synergy of Multi-Chemical Systems and Next-Generation Energy Storage Safety Design: The rise of novel systems such as sodium-ion batteries and all-solid-state batteries brings entirely new thermal failure mechanisms and safety opportunities. For instance, leveraging the superior thermal stability of sodium-ion batteries to configure them as “thermal buffer” units mixed with high-energy lithium batteries allows for active thermal propagation management via spatial layout optimization. Future research should deepen the investigation into the thermo-electrical coupling mechanisms of multi-chemical hybrid modules, constructing a new paradigm of “material-structure–function” integrated safety design to forge next-generation energy storage systems that possess both high energy density and high intrinsic safety.

In summary, research on the thermal safety of lithium-ion batteries is at a critical juncture, undergoing profound evolution from passive protection to active intelligence, from empirical design to quantitative prediction, and from single-function approaches to system integration. In the future, through the deep integration of interdisciplinary fields such as materials science, artificial intelligence, advanced sensing, and multiphysics simulation, it is expected that the inherent trade-off between energy density and safety will be fundamentally overcome, establishing a solid scientific foundation for the realization of truly safe and efficient next-generation energy storage systems.

## Abbreviations

TR: Thermal runaway
LIB: Lithium-ion battery
SEI: Solid-electrolyte interphase
CEI: Cathode-electrolyte interphase
SOC: State of charge
SOH: State of health
BMS: Battery management system
ISC: Internal short circuit
CID: Current interrupt device
PCM: Phase-change material
CPCM: Composite phase-change material
PTC: Positive temperature coefficient
EC: Ethylene carbonate
DMC: Dimethyl carbonate
DEC: Diethyl carbonate
EMC: Ethyl methyl carbonate
VC: Vinylene carbonate
FEC: Fluoroethylene carbonate
LEL: Lower explosion limit
LFP: Lithium iron phosphate (LiFePO_4_)
NCM: Nickel-cobalt-manganese
NCA: Nickel-cobalt-aluminum
LTO: Lithium titanate (Li_4_Ti_5_O_12_)
LMB: Lithium metal battery
ASSB: All-solid-state battery
LATP: Li_1.3_Al_0.3_Ti_1.7_P_3_O_12_
LPSC: Li_6_PS_5_Cl
LLZTO: Li_6.75_La_2_Zr_1.75_Ta_0.25_O_12_
TRN: Thermal runaway number
TRPN: Thermal runaway propagation number
SADT: Safe storage temperature
FPI: Failure proximity index
ISD^2^: Internal strain derivative
L-FTRC: Large-scale fractional thermal runaway calorimetry
BTMS: Battery thermal management system
PVDF: Polyvinylidene fluoride
P3OT: Poly(3-octylthiophene)
P3BT: Poly(3-butylthiophene)
P3DDT: Poly(3-dodecylthiophene)
CNT: Carbon nanotube
MWCNT: Multi-walled carbon nanotube
FBG: Fiber bragg grating
RTD: Resistance temperature detector
EIS: Electrochemical impedance spectroscopy
ARC: Accelerating rate calorimetry
DSC: Differential scanning calorimetry
TGA: Thermogravimetric analysis
XCT: X-ray computed tomography
EELS: Electron energy loss spectroscopy
XPS: X-ray photoelectron spectroscopy
FTIR: Fourier transform infrared spectroscopy
TOF-SIMS: Time-of-flight secondary ion mass spectrometry
DFT: Density functional theory
MD: Molecular dynamics
LSTM: Long short-term memory
GRU: Gated recurrent unit
CNN: Convolutional neural network
GNN: Graph neural network
SVM: Support vector machine
DBSCAN: Density-based spatial clustering of applications with noise
PCC: Polymer current collector
TCC: Thermal control current collector
MOF: Metal-organic framework
HCF: Hollow carbon fiber
LCST: Lower critical solution temperature
MCNN: Model-constrained neural network
DBD: Dielectric barrier discharge
CFRP: Carbon fiber-reinforced polymer
BTMS: Battery thermal management system
